# Zuogui Pill Ameliorates Glucocorticoid-Induced Osteoporosis through ZNF702P-Based ceRNA Network: Bioinformatics Analysis and Experimental Validation

**DOI:** 10.1155/2022/8020182

**Published:** 2022-08-29

**Authors:** Peng Zhang, Honglin Chen, Qi Shang, Guifeng Chen, Jiahui He, Gengyang Shen, Xiang Yu, Zhida Zhang, Wenhua Zhao, Guangye Zhu, Jinglin Huang, De Liang, Jingjing Tang, Jianchao Cui, Zhixiang Liu, Xiaobing Jiang, Hui Ren

**Affiliations:** ^1^The First Clinical School, Guangzhou University of Chinese Medicine, Guangzhou 510405, China; ^2^The First Affiliated Hospital of Guangzhou University of Chinese Medicine, Guangzhou 510405, China; ^3^Lingnan Medical Research Center of Guangzhou Univercity of Chinese Medicine, Guangzhou 510405, China; ^4^Affiliated Huadu Hospital, Southern Medical University, Guangzhou 510800, China

## Abstract

Glucocorticoid-induced osteoporosis (GIOP) is a musculoskeletal disease with increased fracture risk caused by long-term application of glucocorticoid, but there exist few effective interventions. Zuogui Pill (ZGP) has achieved clinical improvement for GIOP as an ancient classical formula, but its molecular mechanisms remain unclear due to scanty relevant studies. This study aimed to excavate the effective compounds and underlying mechanism of ZGP in treating GIOP and construct relative ceRNA network by using integrated analysis of bioinformatics analysis and experimental validation. Results show that ZNF702P is significantly upregulated in GIOP than normal cases based on gene chip sequencing analysis. Totally, 102 ingredients and 535 targets of ZGP as well as 480 GIOP-related targets were selected, including 122 common targets and 8 intersection targets with the predicted mRNAs. The ceRNA network contains one lncRNA (ZNF702P), 6 miRNAs, and 8 mRNAs. Four hub targets including JUN, CCND1, MAPK1, and MAPK14 were identified in the PPI network. Six ceRNA interaction axes including ZNF702P-hsa-miR-429-JUN, ZNF702P-hsa-miR-17-5p/hsa-miR-20b-5p-CCND1, ZNF702P-hsa-miR-17-5p/hsa-miR-20b-5p-MAPK1, and ZNF702P-hsa-miR-24-3p-MAPK14 were obtained. By means of molecular docking, we found that all the hub targets could be effectively combined with related ingredients. GO enrichment analysis showed 649 biological processes, involving response to estrogen, response to steroid hormone, inflammatory response, macrophage activation, and osteoclast differentiation, and KEGG analysis revealed 102 entries with 36 relative signaling pathways, which mainly contained IL-17 signaling pathway, T cell receptor signaling pathway, FoxO signaling pathway, the PD-L1 expression and PD-1 checkpoint pathway, MAPK signaling pathway, TNF signaling pathway, Estrogen signaling pathway, and Wnt signaling pathway. Our experiments confirmed that ZNF702P exhibited gradually increasing expression levels during osteoclast differentiation of human peripheral blood monocytes (HPBMs) induced by RANKL, while ZGP could inhibit osteoclast differentiation of HPBMs induced by RANKL in a concentration-dependent manner. Therefore, by regulating inflammatory response, osteoclast differentiation, and hormone metabolism, ZGP may treat GIOP by regulating hub target genes, such as JUN, CCND1, MAPK1, and MAPK14, and acting on numerous key pathways, which involve the ZNF702P-based ceRNA network.

## 1. Introduction

As the most common secondary osteoporosis, glucocorticoid-induced osteoporosis (GIOP) is a musculoskeletal disease with destruction of bone microstructure, reduced bone mass and strength, and increased risk of fracture caused by long-term application of glucocorticoid (GC) [1]. Statistics show that millions of adults in the world use long-term GCs for treating diseases such as rheumatoid arthritis and bronchial asthma, while some foods and drugs have excessive amounts of hormones, which make many people take GCs invisibly for a long time [2, 3]. Long-term use of GCs often leads to patients suffering from serious complications such as GIOP and fractures, which not only threaten human health, but also bring heavy economic burden for society and family. Existing drugs including vitamin *D*, calcium, denosumab, teriparatide, and bisphosphonates serve as recommended therapies for the treatment of GIOP [4], but long-term use of them trigger some side effects causing rapid bone loss and increasing the risks of jaw osteonecrosis, atypical femoral fractures, and multiple rebound-related vertebral fractures [5]. The clinical management of GIOP is still debated, so it is indispensable for developing a new drug therapy against GIOP [6].

Recently, substantial achievements have been made against GIOP from the perspective of regulating bone homeostasis [7]. However, the ambiguity of potential targets and the diversity of therapeutic drugs have become the bottle-neck for clinically preventing and treating GIOP. Therefore, it has become a pressing clinical need to study the pathogenesis of GIOP and find potential biomarkers against GIOP.

Research shows that GCs can not only inhibit BMSC and osteoblast proliferation as well as preosteoblast differentiation, and promote apoptosis and autophagy in osteoblasts, but also influence osteoclast differentiation and extend osteoclast lifespan, among which miRNA, autophagy, and apoptosis play a crucial part in mediating bone homeostasis in GIOP [3]. In addition, GCs can indirectly mediate bone homeostasis by regulating sex hormones and neuromuscular system [8]. At the same time, our previous studies have also found that miRNAs and autophagy play a key role in the pathogenesis of GIOP, and further confirmed that the imbalance of bone homeostasis is the basic pathological mechanism of GIOP, and the decline of bone formation runs through the onset and progression of GIOP [9, 10].

Long noncoding RNAs (lncRNAs) are a kind of endogenous noncoding RNAs with a length of more than 200 bp and nonlong open reading frame [11, 12]. Studies have revealed that lncRNAs play a vital role in embryonic development, cell proliferation and differentiation, and organogenesis [13–15], and lncRNAs can regulate epigenetic, transcriptional, and post-transcriptional functions [16]. Some lncRNAs can serve as competing endogenous RNAs (ceRNAs) to absorb miRNAs, and thus participate in the expression regulation of target genes [17]. The ceRNA network is an intrinsic mechanism of RNA interaction and regulation. However, it remains unclear whether this mechanism plays a role in regulating bone homeostasis and whether it is involved in the pathogenesis of GIOP.

Traditional Chinese medicine (TCM) has accumulated a wealth of clinical practice experience in the treatment of osteoporosis-related diseases, which has the advantages of mild efficacy, long-lasting effect, low side effects, and long-term use [18]. Zuogui Pill (ZGP), as an ancient classical formula, has been widely used for clinically treating bone diseases like GIOP and fracture [19, 20]. Some studies have demonstrated that ZGP may prevent GIOP in zebrafish larvae by reversing bone formation/resorption imbalance and activating the TGF-*β*-Smad signal [20]. Additionally, ZGP could upregulate the expression of the vital signal molecules in the Wnt signaling pathway including Wnt1, LRP-5, and *β*-catenin so as to prevent and treat GIOP [19]. Moreover, our previous studies have confirmed that ZGP could prevent and treat GIOP possibly by downregulating DKK1 mRNA expression [21] or upregulating mTORC1 mRNA expression [22], which could be important targets for preventing and treating GIOP [23]. However, there exist few studies to focus on the ceRNA network involved in the mechanism of ZGP against GIOP. Therefore, this present study aimed to investigate differences in vertebral bone tissue RNA expression data between GIOP patients and normal patients, excavate the effective compounds and underlying mechanism of ZGP in treating GIOP, and construct relative ceRNA network by using integrated analysis of gene chip sequencing, network pharmacology, and experimental validation.

## 2. Materials and Methods

### 2.1. Identification of the Differentially Expressed lncRNA in Vertebral Bone Tissue of GIOP Patients Compared to Normal

The total RNA was extracted from vertebral bone tissue of GIOP patients (*n* = 3) and normal patients (*n* = 3) by using Trizol reagents (Invitrogen). The extracted total RNA samples were subjected to agarose electrophoresis and Nanodrop quality inspection and quantification. Oligo magnetic beads were used to enrich the lncRNA, and the KAPA Stranded RNA-Seq Library Prep Kit (Illumina, Aksomics, Shanghai) was used to construct the library. The constructed library was checked by Agilent 2100 Bioanalyzer (Aksomics, Shanghai), and the final quantification of the library was performed by qPCR. According to the quantitative results and the final sequencing data, the sequencing libraries of different samples were mixed into the sequencing process. The screening thresholds to determine the differentially expressed lncRNA were *P* value <0.05 and |log_2_ fold change (FC)| > 1. The present study was approved by the Ethics Committee of the 1st Affiliated Hospital of Guangzhou University of Chinese Medicine (GZUCM) with the approval number ZYYECR[2016]028.

### 2.2. Prediction of Differentially Expressed lncRNA-miRNA Interactions and miRNA-mRNA Interactions

The miRcode database (http://www.mircode.org/) was used to predict lncRNA-miRNA interactions. Then the miRDB database (http://www.mirdb.org/), miRTarBase database (http://mirtarbase.mbc.nctu.edu.tw/php/index.php), and TargetScan database (http://www.targetscan.org/) were used to predict the miRNA-mRNA interactions, and only the interactions included in all three databases were selected.

### 2.3. Obtaining the Bioactive Components and Targets of ZGP Drugs

The bioactive components and targets of ZGP drugs including Di Huang, Shan Yao, Gou Qi Zi, Shan Zhu Yu, Niu Xi, Tu Si Zi, Gui Ban, and Lu Jiao were obtained through BATMAN database(http://bionet.ncpsb.org/batman-tcm/) with “score cutoff” set to 48, “P value cutoff” set to 0.05, and “popular organisms” set as humans [19]. Moreover, the active compounds of ZGP drugs were retrieved through the TCMSP database (http://tcmspw.com/tcmsp.php). Gui Ban and Lu Jiao are not included in the TCMSP database, so the bioactive components of Gui Ban and Lu Jiao reported in literature were searched manually to make a supplementary summary. The above components were screened with oral bioavailability (OB) > 30% and drug likeness (DL) > 0.18 to obtain the eligible compounds and their corresponding targets from the TCMSP database [24]. Finally, the bioactive components and targets obtained from the TCMSP platform and BATMAN database were summarized and deduplicated.

### 2.4. Gene Targets of GIOP

The key word “glucocorticoid-induced osteoporosis” was searched in the GeneCards database (https://www.genecards.org/) [25] with the species set as “Homo sapiens.”

### 2.5. Construction of Drug-Compound-Target Network

Using *R* software (v3.6.1), the targets of GIOP were mapped with the targets of ZGP to obtain the common targets. Then the drugs and compounds corresponding to the common targets were identified and Drug-Compound-Target Network was constructed.

### 2.6. Construction of ceRNA Network

The overlap between the common targets and the predicted mRNAs mentioned in 2.2 was taken as the intersection targets. The intersection targets as mRNA in ceRNA network were selected and lncRNA-miRNA-mRNA interactions were identified to construct the ceRNA network by Cytoscape3.7.2 (http://www.cytoscape.org/).

### 2.7. Protein-Protein Interaction (PPI) Analysis

The STRING database (https://string-db.org/) was retrieved to get the PPI data of the intersection targets. Next, the PPI information of the intersection targets was input into Cytoscape (v3.7.2) software to construct the PPI network and calculate degrees and betweenness centralities of targets in the network through network topology analysis. The targets whose degrees and betweenness centralities were above average were determined to be the hub targets.

### 2.8. Molecular Docking

AutoDock Vina (v1.1.2) software [26] was utilized to carry out molecular docking simulations between hub targets and their corresponding compounds to verify their interaction activity. The Pubchem database (https://pubchem.ncbi.nlm.nih.gov/) was searched for the 3D structure of compounds. AutoDock Tools (v1.5.6) were used to distribute charge and combine nonpolar hydrogen for compounds and convert the results into a PDBQT file. The crystal structures of target proteins from the RCSB PDB website (http://www.rcsb.org/) were downloaded. Then the target protein was separated from its ligand, added polar hydrogen, and distributed charge via AutoDock Tools, which would be subsequently stored as a PDBQT file. AutoDock Tools were also utilized to calculate the center and size of the docking box. Molecular docking simulations among the target proteins and compounds were performed with every affinity calculated. Then PyMol were used to draw and analyze the docking results.

### 2.9. GO Enrichment Analysis and KEGG Pathway Analysis

Gene Ontology (GO) enrichment analysis concerning biological process (BP) via the clusterProfiler package (R3.6.1) was performed and the enrichment results with *P* < 0.05 were selected. Then the 20 representative items closely related to the pathological process of GIOP were presented. Next, we carried out Kyoto Encyclopedia of Genes and Genomes (KEGG) analysis of the intersection targets using the clusterProfiler package (R3.6.1), extracted the significant enrichment results (*P* < 0.05), and plotted pathway-target network using Cytoscape.

### 2.10. Experimental Validation by *In Vitro* Assays

#### 2.10.1. Cells and Reagents

The source of ZGP was obtained from the 1st Affiliated Hospital of GZUCM. Recombinant human TRANCE/TNFSF11/RANKL (6449-TEC) was purchased from R&D Systems (Minneapolis, MN. United States). Recombinant human M-CSF protein (11792-H08Y) was purchased from China Bio (Beijing). The cell counting kit-8 (CCK-8) was purchased from Beyotime, while TRAP working solution was obtained from Sigma Aldrich (St.Louis, MO, USA).

#### 2.10.2. Human Peripheral Blood Monocyte Culture and Assay

The clinical experiments involved in this paper were authorized by the Ethics Committee of the 1st Affiliated Hospital of GZUCM (No. K[2019]129). In the current research, all patients who participated in this trial were provided informed consent at the beginning. Then, 10-mL of external venous blood was drawn from volunteer patients (*n* = 3). The manipulation of human peripheral blood monocytes (HPBMs) was performed as described previously [27]. First, 10-mL of whole blood from patients was put into a 50-mL centrifuge tube, then diluted with 10-mL of PBS and gently mixed. Afterward, we continuously centrifuged the initial blood specimen at 2000 rpm for 20 minutes. When centrifugation was finished, the blood sample was stratified and the leukocyte layer in the center of the sample containing HPBMs was aspirated by pipette and transferred to a single fresh 15 mL centrifuge tube in liquid with 10–15 mL of PBS. Next, the solution was centrifuged at 1500 rpm for 10 min and the supernatant was lifted to precipitate and be the wanted HPBMs. HPBMs were resuspended in 10 mL of medium containing 20 ng/mL hM-CSF protein and subsequently transferred to culture dishes to incubate for 5 days. Then tartrate-resistant acid phosphatase (TRAP) staining was performed to identify osteoclasts, which are TRAP-positive (TRAP^+^) cells with more than 3 nuclei. When the incubation time was reached, HPBMs turned into TRAP^+^ mature osteoclasts after 10 days of cell intervention using 50 ng/mL of hRANKL.

#### 2.10.3. Cell Counting Kit 8 Assay

The HPBMs were treated with a concentration gradient of ZGP for 3, 5, and 7 days after induction of osteoclast development. According to the manufacturer's protocol, we performed a cell counting kit 8 (CCK-8) assay to detect cell proliferation abilities using an optical density (OD) setting of 450 nm in the microplate reader (Varioskan Flash; Thermo Fisher Scientific, Waltham, MA, USA).

#### 2.10.4. RNA Extraction and Real-Time Quantitative Polymerase Chain Reaction (RT-qPCR)

HPBMs were inoculated in 6-well plates at a cell count of 2 × 10^5^ cells. After induction of osteoclast development, 1 mL of Trizol reagent was applied to each well for total RNA extraction from the cells. Subsequently, retrotranscription of 1 *μ*g of total RNA was performed using a cDNA synthesis kit (Takara Inc.Shiga, Japan). 20 *μ*L of SYBR Green qPCR SuperMix (Takara Inc.) was used for detection of *ZNF702P* cDNAs and RT-qPCR machine (Bio-Rad, Hercules, CA, USA). The thermal cycling conditions for the final gene amplification were: 95°C for 30 s, 40 cycles of 95°C for 5s, and a final step of 60°C for 30s. Quantitative analysis was performed using the 2ΔΔCT method for the calculation of the relative expression of each gene. The gene-related detection primers of *ZNF702P* (Forward: ACAAGGCATTCGGGTGTGAT; Reverse:ACCACTGAAGGCTCTGTCAC) were compounded by Shanghai Sangon Biotechnology Co.Ltd (China).

### 2.11. Statistical Analysis

All results were expressed as mean ± standard deviation. Student's *t*-tests were used to compare two separate samples. One-way ANOVA was used for comparison of univariate samples between multiple groups. , *P* value <0.05 indicates statistical significance.

## 3. Results

### 3.1. Differential Expression Analysis of lncRNA in GIOP

**Figure 1:**
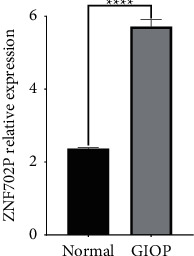
Differential expression analysis of lncRNA in GIOP. Data are displayed as mean ± standard deviation. ^*∗∗∗∗*^*P* < 0.0001.

To explore differential expressed lncRNA in GIOP, vertebral bone tissue of GIOP patients and normal patients was analyzed, showing that ZNF702P was significantly upregulated in GIOP (*P* < 0.0001), which means that ZNF702P could be a potential biomarker to identify GIOP, as shown in Figure 1.

### 3.2. lncRNA-miRNA-mRNA Interactions

Totally, 75 ZNF702P-miRNA interactions and 936 miRNA-mRNA interactions were obtained. After removing duplication, there were 621 mRNAs predicted for subsequent analysis, as shown in Supplementary Table 1.

### 3.3. The Active Compounds and Targets of ZGP

Based on BATMAN database and TCMSP platform, 120 active compounds of ZGP were obtained including 7 compounds from Niu Xi, 3 compounds from Di Huang, 40 compounds from Gou Qi Zi, 4 compounds from Gui Ban, one compound from Lu Jiao, 22 compounds from Shan Yao, 32 compounds from Shan Zhu Yu, and 11 compounds from Tu Si Zi. After the duplicates were removed, 102 bioactive components and 535 targets of ZGP were screened, as shown in Supplementary Table 2.

### 3.4. The Common Targets of ZGP and GIOP

**Figure 2:**
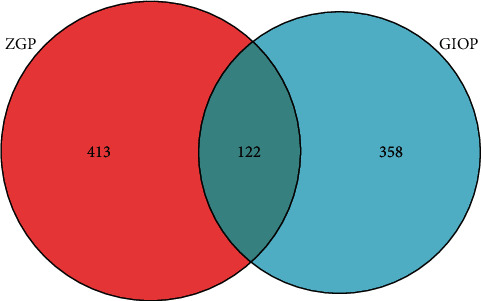
Venn diagram of ZGP-GIOP common targets.

Through the retrieval of GeneCards database, we obtained a total of 480 gene targets of GIOP. After they were mapped with the targets of ZGP, totally 122 common targets were obtained, as demonstrated in Figure 2 and Table 1.

### 3.5. Construction of the Drug-Compound-Target Network

**Figure 3:**
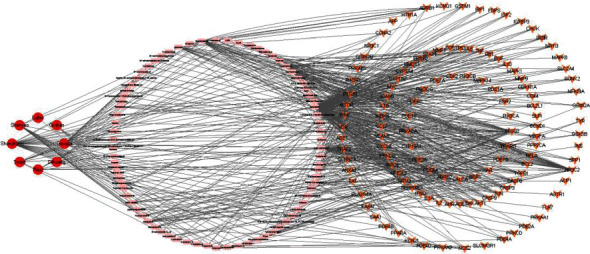
Drug-compound-target network.

The correspondence among the drugs, compounds, and common targets of ZGP in treating GIOP was visualized by Cytoscape, as demonstrated in Figure 3. The network contained 208 nodes and 610 edges, including 8 drug nodes, 78 compound nodes, and 122 target nodes. In Figure 3, the red circle nodes stand for the drugs, the pink triangle nodes stand for active compounds, and the orange V-shape nodes stand for targets. The edges stand for the corresponding relationship among drugs, compounds, and common targets of ZGP in treating GIOP.

### 3.6. Construction of the ceRNA Network

**Figure 4:**
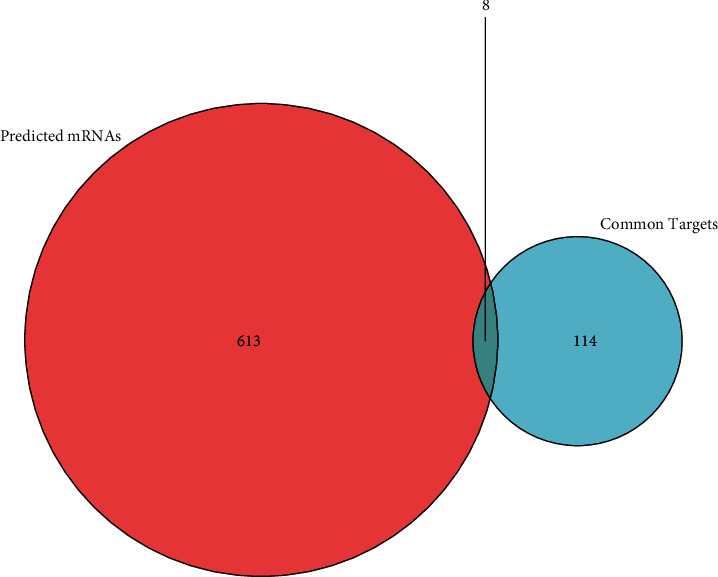
The overlap between the common targets and the predicted mRNAs.

**Figure 5:**
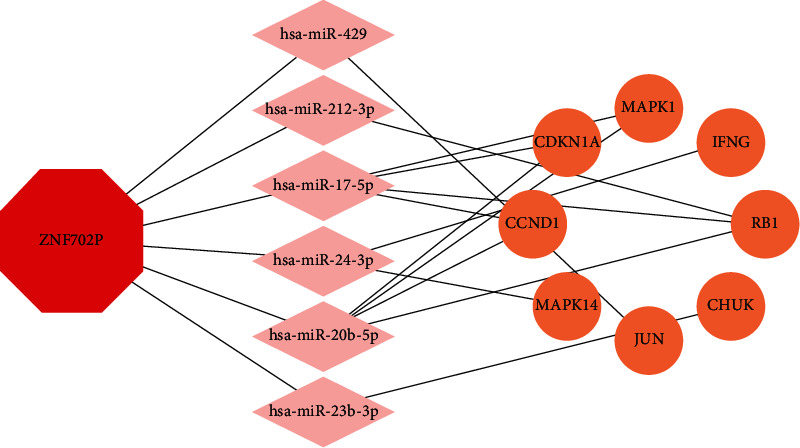
The ceRNA network.

There are 8 intersection targets between the predicted mRNAs and the common targets, as shown in Figure 4. Then we constructed the ceRNA network through ZNF702P-miRNA-mRNA interactions, as shown in Figure 5.

### 3.7. PPI Network Construction and Hub Target Screening

**Figure 6:**
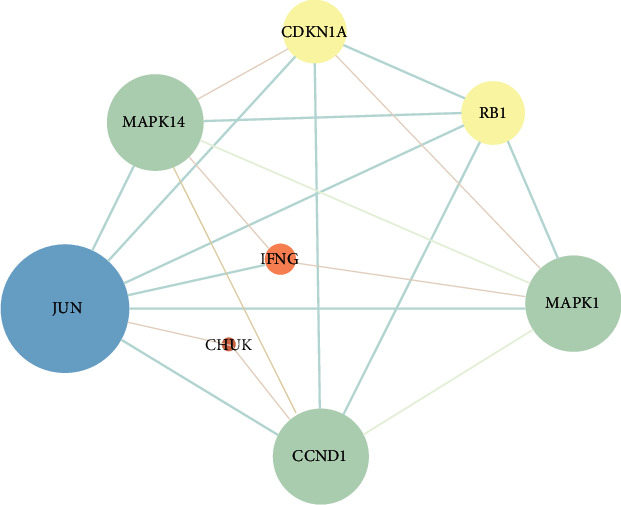
PPI network of intersection targets.

The intersection targets were imported into the STRING database. And then we imported the PPI data into Cytoscape (v 3.7.2) to draw the PPI network in Figure 6. There were 4 targets including JUN, CCND1, MAPK1, and MAPK14 whose degrees and betweenness centralities were above the average, which were predicted as the hub targets. The detailed network topology information was shown in Supplementary Table 3.

### 3.8. Molecular Docking Validation

**Figure 7:**
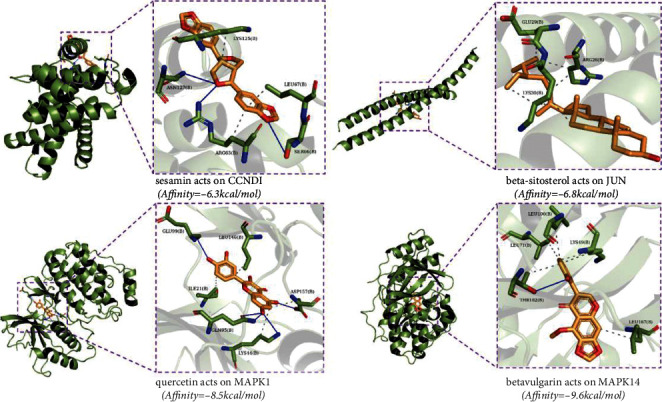
Detailed target-compound interactions with the highest molecular docking affinities.

The 3D structures of the 4 hub genes were obtained through the RCSB PDB database. According to Table 2, all the hub targets demonstrated good and stable binding activity with their bioactive compounds. Then the docking results of all the hub targets with the strongest binding ability were visualized in Figure 7. For example, sesamin combined with CCND1 by forming Pi-alkyl bonds with the residues including Arg-63 and Leu-67 (docking affinity: -6.3 kcal/mol). The docking affinity of beta-sitosterol on JUN was -6.8 kcal/mol. The residues containing Arg-26 and Lys-30 were linked to beta-sitosterol by forming alkyl bonds. The docking affinity of quercetin on MAPK1 was -8.5 kcal/mol. There existed hydrogen bonds provided by the Gln-95, Glu-99, and Asp-157 residues in the link to quercetin. The docking affinity of beta-vulgarin on MAPK14 was -9.6 kcal/mol. The residue Thr-102 formed one hydrogen bond in the interaction with beta-vulgarin. In addition, the residues Leu-100, Leu-71, Lys-49, and Leu-167 provided a powerful electrostatic force for the combination of beta-vulgarin and MAPK14.

### 3.9. GO Enrichment Analysis

**Figure 8:**
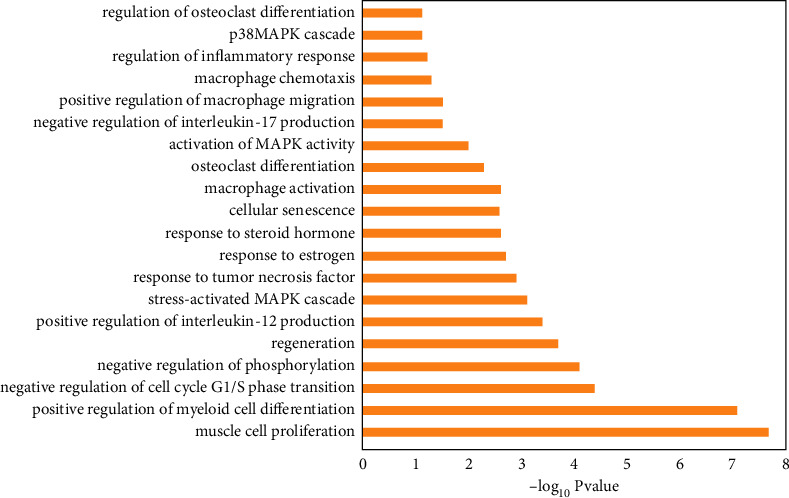
GO, BP enrichment analysis.

Totally 649 items of biological process (BP) were obtained, among which the filtrated 20 items involving anti-GIOP effects of ZGP were closely correlated with cellular senescence, negative regulation of phosphorylation, activation of MAPK activity, response to estrogen, response to steroid hormone, inflammatory response, macrophage activation and osteoclast differentiation, etc. GO,BP enrichment analysis results are shown in Figure 8.

### 3.10. KEGG Pathway Analysis

**Figure 9:**
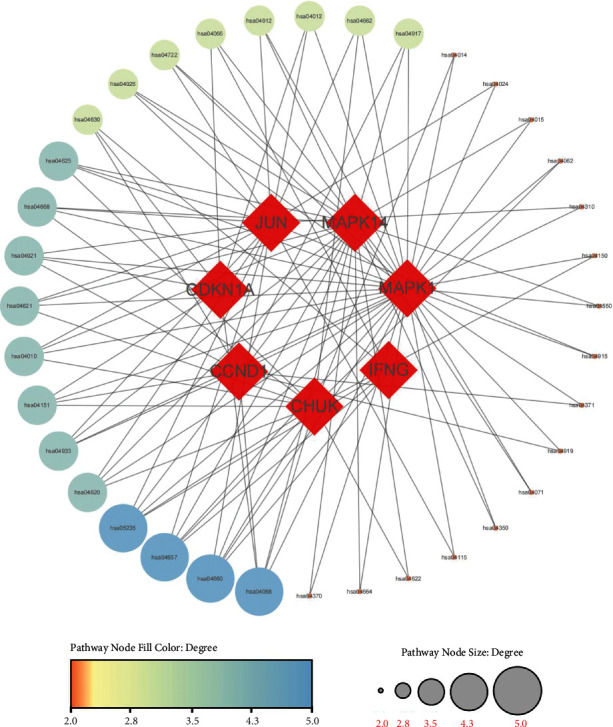
Pathway-target network.

The KEGG pathway enrichment analysis of 8 target genes was conducted using *R* software. We finally got 102 items including 36 key signaling pathways, as described in Table 3. We conducted network visualization via Cytoscape as plotted in Figure 9. According to Figure 9, we have deeply filtered the key signaling pathways, and selected four core signaling pathways, including the IL-17 signaling pathway, T cell receptor signaling pathway, FoxO signaling pathway, PD-L1 expression and PD-1 checkpoint pathway, which may play an important role in the pathophysiology of GIOP.

### 3.11. RT-qPCR Analysis

**Figure 10:**
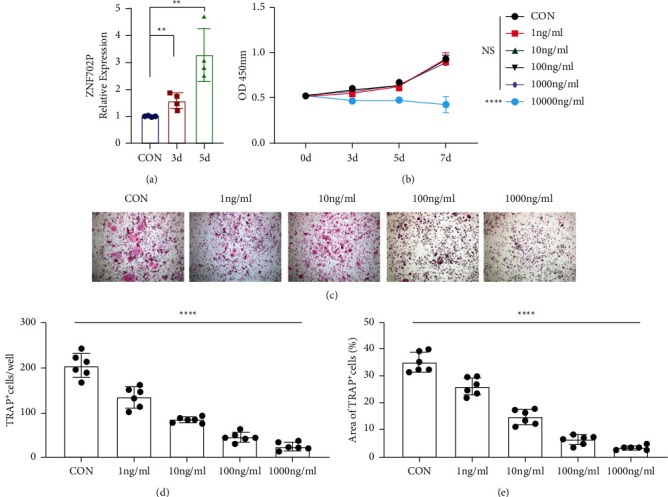
Experimental validation by *in vitro* asays (a) RT-qPCR validated that ZNF702P exhibited gradually increasing expression levels during osteoclast differentiation of HPBMs induced by RANKL. (b) CCK-8 assays with different concentrations of ZGP. (c) TRAP staining analysis showed that ZGP could inhibit osteoclast differentiation of HPBMs induced by RANKL in a concentration-dependent manner. Histograms of the count (d) and area (e) of TRAP^+^ multinucleated cells per well. Data are displayed as mean ± standard deviation. ^*∗∗*^*P* < 0.01; ^*∗∗∗∗*^*P* < 0.0001.

In contrast to the control group, ZNF702P exhibited gradually increasing expression levels during osteoclast differentiation of HPBMs induced by RANKL from 3 days to 5 days (Figure 10(a)). It suggests that the expression level of ZNF702P is positively correlated with osteoclast differentiation.

### 3.12. CCK-8 Analysis

CCK-8 results showed that there was no cytotoxicity to HPBMs when the ZGP concentrations were no higher than 1000 ng/mL with the proliferation of HPBMs neither promoted nor inhibited, which were selected for subsequent experiments (Figure 10(b)).

### 3.13. TRAP Staining Analysis

After HPBMs were induced to osteoclast differentiation, the effects of ZGP were detected during this process (Figure 10(c)). TRAP staining results revealed that the number and area of TRAP^+^ cells decreased rapidly with increasing ZGP concentration, suggesting that ZGP could inhibit osteoclast differentiation (Figures 10(d)–10(e)).

## 4. Discussion

GIOP is a systemic bone disease secondary to glucocorticoid intake, which is the most common secondary osteoporosis [28]. Although numerous protein-coding genes have been identified to be GIOP-related genes [29], these genes could not give a good interpretation to the onset and development of GIOP. Currently, multiple studies have focused on the epigenetic regulation and the roles of lncRNAs in the pathogenesis of osteoporosis [30]. In this study, we constructed the ZNF702P-associated ceRNA network according to the expression profiles of vertebral bone tissues between GIOP patients and healthy controls.

Chinese traditional formula Zuogui Pill has been widely used for clinically treating GIOP for many years as an ancient classical formula [20,31]. Zuogui Pill has been confirmed to exert ameliorative effects on postmenopausal osteoporosis in rat by regulating transduction of coupling signals between osteoblast and osteoclast as well as the differentiation of osteoclasts [19]. ZGP can upregulate the expression of the crucial molecules in the Wnt signaling pathway, including Wnt1, LRP-5, and *β*-catenin, thus preventing and treating GIOP in rats [20]. ZGP can also activate osteogenic differentiation of BMSCs [32].

In our previous studies, we have established GIOP rat model with subcutaneous injection of dexamethasone, and we have confirmed that ZGP treatment could ameliorate GIOP with enhanced volumetric bone mineral density, bone mineral content, trabecular bone volume fraction, trabecular number, and vertebral compressive strength [21, 22]. Moreover, we have found that Gui Ban (*Plastrum Testudinis*), one main drug in Zuogui Pill, may reverse GIOP by targeting OPG, Runx2, and CTSK [33]. To our knowledge, few studies have been reported on the regulation of mRNA expression and protein levels through ZNF702P-based ceRNA network in the treatment of GIOP by ZGP. These complex networks may provide multiple clues to elucidate the pathogenesis of GIOP. Therefore, this study first explored ZNF702P as a potential biomarker, thus exerting therapeutic effect of ZGP on GIOP.

To identify the core of ceRNA networks, we got 4 hub targets including JUN, CCND1, MAPK1, and MAPK14 by PPI analysis. Then AutoDock Vina was used to verify the binding activity among hub targets and their related components with binding energy less than -5.0 kcal/mol, which showed good and stable combination with each other [34]. According to drug-compound-target network and molecular docking results, the active compounds with high values consisted of quercetin, sesamin, beta-sitosterol, and kaempferol. Quercetin is identified as an antiosteoporotic flavonoid, which promotes osteogenesis, antioxidant expression, angiogenesis, osteoclast, and adipocyte apoptosis, while inhibiting RANKL-mediated osteoclastogenesis, osteoblast apoptosis, oxidative stress, and inflammatory response [35]. Sesamin, a member of the lignan family, has estrogenic activity and plays an important role in healing osteoporotic fracture by activating angiogenesis and chondrogenesis [36]. Moreover, existing study reveals that sesamin could promote osteogenesis by upregulating the Wnt/*β*-catenin pathway and inhibit osteoclastogenesis by downregulating the NF-*κ*B pathway, suggesting that it could be a therapeutic medication for osteoporosis treatment [37]. As a major phytosterol in plants, beta-sitosterol has medical benefit of bone strengthening, which was reported to exert protective function on GIOP in rats via the RANKL/OPG pathway [38]. Kaempferol, as a natural anti-inflammatory flavonoid, has been reported to have curative effects on ameliorating GIOP via activating the JNK and the p38-MAPK signaling pathways in dexamethasone-treated MC3T3-E1 cells and improving bone mineralization [39].

The pathophysiology of GIOP is related to inhibiting the proliferation of osteoblasts, promoting the apoptosis of osteoblasts and lengthening the lifespan of osteoclasts, which has received increasing attention [4]. JUN is an important molecule on the MAPK signaling pathway, which is considered as a leading factor mediating RANKL and affecting osteoclastogenesis [40]. Mitigating the downstream levels of c-Jun contributes to inactivating the NF-*κ*B/MAPK signaling pathway and NFATc-1, which suppresses the expression of osteoclast-specific genes and inhibits osteoclastogenesis [41]. CCND1(Cyclin D1) is an important gene to regulate the cell cycle, which is closely linked with bone formation [42]. The glucocorticoid receptor (GR) represses CCND1 via Tcf-*β*-catenin, the transcriptional effector of the Wnt signaling pathway [43]. The downregulation of CCND1 expression could inhibit osteogenic proliferation and differentiation of MC3T3-E1 cells, as MC3T3-E1 cell apoptosis targeting CCND1 might be regulated by the Wnt/*β*-catenin pathway [31]. Therefore, CCND1 may be a potential biomarker against GIOP.

MAPK1 is a key molecule in the inflammatory response of chondrocytes [44]. The inhibition of MAPK1 could enhance apoptosis induced by GC [45]. Moreover, MAPK1 is a regulatory factor on the ERK signaling pathway involved in regulation of osteoblast differentiation which may be a promising target in preventive and therapeutic strategies for GIOP [46]. MAPK14 is one of the four p38 MAPKs, which plays a key role in physical stress or proinflammatory cytokines leading to direct activation of transcription factors [47]. In mammals, phosphorylated-MAPK14 is able to transcriptionally activate Serum Glucocorticoid Kinase 1(SGK1) [48], which is a regulator in osteoclastogenesis and bone homeostasis [49].

Importantly, six key ceRNA interaction axes including ZNF702P-hsa-miR-429-JUN, ZNF702P-hsa-miR-17-5p/hsa-miR-20b-5p-CCND1, ZNF702P-hsa-miR-17-5p/hsa-miR-20b-5p-MAPK1, and ZNF702P-hsa-miR-24-3p-MAPK14 were obtained. Evidence has revealed that overexpression of miR-429 promoted MC3T3-E1 cell differentiation, and enhanced matrix mineralization and alkaline phosphatase activity [50]. Some studies have demonstrated that miR-429 downregulated JUN expression [51]. Research has confirmed that miR-17-5p could suppress osteogenic differentiation and inhibit bone formation [52]. It has been revealed that miR-17-5p upregulated CCND1 expression [53], but downregulated the expression of MAPK1 mRNA [54]. It has been reported that miR-20b-5p exhibited regulatory effect on the Wnt signaling pathway related to osteogenesis [55], while overexpression of miR-20b-5p downregulated CCND1 expression [56]. And some studies also showed that miR-20b-5p was highly linked to MAPK1 [57]. Additionally, miR-24-3p serves as a regulatory factor of Smad5, which exerts important functions on osteogenic differentiation [58]. Also, evidence demonstrated that the overexpression of miR-24-3p upregulated the phosphorylation activity of MAPK14 [59].

Then we conducted GO and KEGG enrichment analyses of the intersection targets and identified not only multiple biological processes correlated with GIOP, including cellular senescence, activation of MAPK activity, response to estrogen, response to steroid hormone, the differentiation of osteoclast, the activation of macrophages, and inflammatory response, but also numerous signaling pathways, including the IL-17 signaling pathway, T cell receptor signaling pathway, FoxO signaling pathway, PD-L1 expression and PD-1 checkpoint pathway, MAPK signaling pathway, TNF signaling pathway, Estrogen signaling pathway, and Wnt signaling pathway. In general, the functional analyses focused on three aspects including regulation of inflammatory response, cell cycle-like osteoclast differentiation, and hormone metabolism, all of which take part in the pathogenesis of GIOP. Notably, osteoclast differentiation is activated and promoted during the progression of GIOP [5]. Our *in vitro* experiments confirmed that ZNF702P exhibited gradually increasing expression levels during osteoclast differentiation of HPBMs induced by RANKL. The expression level of ZNF702P is positively correlated with osteoclast differentiation, which is consistent with the results in Figure 1, indicating that ZNF702P could serve as a biomarker of osteoclastogenesis activation, which plays an important role in GIOP. Moreover, our *in vitro* experiments validated that ZGP could inhibit osteoclast differentiation of HPBMs induced by RANKL in a concentration-dependent manner. It is speculated that ZGP may suppress the expression levels of ZNF702P in HPBMs to inhibit osteoclast differentiation so as to anti-GIOP.

According to the KEGG pathway analysis, there are several signaling pathways worthy to explore in the future researches. For instance, the IL-17 signaling pathway participates in regulating osteoclast differentiation, and the IL-17 signaling pathway can stimulate the synthesis of TNF-*α*, IL-6, and NF-кB, thereby promoting RANKL-induced osteoclast differentiation [60]. Moreover, studies have shown that the inflammatory factor, TNF-*α*, can promote RANKL expression and induce osteoclast formation [61,62]. So, the IL-17 signaling pathway [63] and the TNF signaling pathway [64] are closely correlated with the regulation of osteoclasts by the OPG/RANKL/RANK system. Therefore, the IL-17 and TNF signaling pathways might exert important functions in the process of ZGP treatment against GIOP, which needs further identification. PD-L1 expression and PD-1 checkpoint pathway is closely linked to bone homeostasis, and lack of members in this pathway leads to deterioration of bone structure [65]. The FoxO signaling pathway exerts essential effects on regulating bone cell functions including bone development, remodeling, and homeostasis, which contributes to osteoporosis [66]. The T cell receptor signaling pathway is reported to be associated with bone loss [67]. Modern studies have confirmed that the inhibition of the MAPK signaling pathway can suppress osteoclastogenesis and bone resorption [68]. Based on the KEGG pathway analysis, osteoclast-specific genes including MAPK1, MAPK14, and JUN were enriched in the MAPK signaling pathway. Thus, we speculate that ZGP might regulate the expression of MAPK1, MAPK14, and JUN on MAPK signaling way so as to inhibit osteoclast differentiation and bone resorption, which may be the potential mechanism of ZGP treating GIOP. Notably, the estrogen signaling pathway can combine the Wnt signaling pathway and the protein kinase pathway to exert regulatory functions on osteoblasts' and osteoclasts' proliferation, apoptosis, and differentiation [69]. Moreover, both the MAPK and estrogen signaling pathways have been reported to regulate bone formation and bone mass control [70].

Collectively, our results predicted some potential therapeutic targets and pathways, providing reference for future studies on ZGP treatment against GIOP. However, one limitation of this study is that further *in vivo* and *in vitro* experiments are needed to confirm our findings.

## 5. Conclusion

By regulating inflammatory response, osteoclast differentiation, and hormone metabolism, ZGP may treat GIOP by regulating hub target genes, such as JUN, CCND1, MAPK1, and MAPK14, and acting on numerous key pathways, which involve ZNF702P-based ceRNA network. Our findings firstly offered novel insights into the roles of ZNF702P-based ceRNA interaction axes in the pathogenesis of GIOP and provided potential diagnostic biomarkers. However, the specific mechanism and material basis still need to be further verified *in vivo* and *in vitro*.

## Figures and Tables

**Table 1:** Potential target genes of ZGP in the treatment of GIOP.

Number	Gene	Number	Gene	Number	Gene
1	NR3C2	42	HMOX1	83	TAT
2	NOS2	43	CYP3A4	84	PLA2G4A
3	PTGS1	44	CYP1A2	85	FASN
4	ESR1	45	MYC	86	MTOR
5	AR	46	ICAM1	87	SAA1
6	PPARG	47	IL1B	88	PDE4B
7	PTGS2	48	CCL2	89	PDE4D
8	ESR2	49	VCAM1	90	PDE4A
9	MAPK14	50	CXCL8	91	ANXA1
10	GSK3B	51	PRKCB	92	SLC9A3R1
11	CDK2	52	BIRC5	93	TYR
12	PIK3CG	53	IL2	94	PRKAB1
13	PRKACA	54	NR1I2	95	KCNQ1
14	PDE3A	55	SERPINE1	96	PRKCD
15	ADRB2	56	IFNG	97	PDE2A
16	BCL2	57	IL1A	98	SCNN1A
17	BAX	58	NFE2L2	99	PRKAA1
18	CASP9	59	AHR	100	TLR7
19	JUN	60	SLC2A4	101	KCNJ1
20	CASP3	61	NR1I3	102	AGTR1
21	CASP8	62	INSR	103	ACP1
22	TGFB1	63	PPARA	104	PRKAA2
23	RELA	64	CHUK	105	INS
24	AKT1	65	SPP1	106	CALCA
25	VEGFA	66	RUNX2	107	PDE7B
26	CCND1	67	IGFBP3	108	PIK3CA
27	BCL2L1	68	IGF2	109	PDE1A
28	FOS	69	IRF1	110	PIK3CB
29	CDKN1A	70	GSTM1	111	PDE7A
30	MMP9	71	ADRB1	112	PDE4C
31	MAPK1	72	GRIN2A	113	PDE1C
32	IL10	73	GRIN2B	114	CYP19A1
33	EGF	74	HTR1A	115	PDE8A
34	RB1	75	APP	116	PIK3R1
35	TNF	76	CCNA2	117	PPP2CA
36	IL6	77	NR3C1	118	ACE
37	TP53	78	PIM1	119	SLPI
38	NFKBIA	79	VDR	120	CD44
39	MMP1	80	ASS1	121	IKBKB
40	STAT1	81	TRAF2	122	MAPK8
41	ERBB2	82	FDPS		

**Table 2:** Docking scores of hub targets with their bioactive compounds.

Targets	PDB ID	Compounds	Affinity (kcal/mol)
CCND1	2W9F	Quercetin	−5.7
CCND1	2W9F	Sesamin	−6.3
JUN	1JNM	Beta-sitosterol	−6.8
JUN	1JNM	Quercetin	−5.2
JUN	1JNM	Kaempferol	−5.3
MAPK1	5NHV	Quercetin	−8.5
MAPK14	2GTN	Beta-vulgarin	−9.6
MAPK14	2GTN	Glycitein	−8.7
MAPK14	2GTN	Isorhamnetin	−9.0

**Table 3:** KEGG pathway enrichment analysis.

Id	Signaling pathway	Enriched genes	*P* value
hsa05235	PD-L1 expression and PD-1 checkpoint pathway	CHUK/IFNG/MAPK1/MAPK14/JUN	0.000000008
hsa04657	IL-17 signaling pathway	CHUK/IFNG/MAPK1/MAPK14/JUN	0.000000010
hsa04660	T cell receptor signaling pathway	CHUK/IFNG/MAPK1/MAPK14/JUN	0.000000017
hsa04068	FoxO signaling pathway	CHUK/MAPK1/CDKN1A/CCND1/MAPK14	0.000000055
hsa04933	AGE-RAGE signaling pathway in diabetic complications	MAPK1/CCND1/MAPK14/JUN	0.000001473
hsa04620	Toll-like receptor signaling pathway	CHUK/MAPK1/MAPK14/JUN	0.000001724
hsa04625	C-type lectin receptor signaling pathway	CHUK/MAPK1/MAPK14/JUN	0.000001724
hsa04668	TNF signaling pathway	CHUK/MAPK1/MAPK14/JUN	0.000002322
hsa04921	Oxytocin signaling pathway	MAPK1/CDKN1A/CCND1/JUN	0.000008283
hsa04621	NOD-like receptor signaling pathway	CHUK/MAPK1/MAPK14/JUN	0.000015728
hsa04917	Prolactin signaling pathway	MAPK1/CCND1/MAPK14	0.000033537
hsa04662	B cell receptor signaling pathway	CHUK/MAPK1/JUN	0.000053955
hsa04012	ErbB signaling pathway	MAPK1/CDKN1A/JUN	0.000060091
hsa04912	GnRH signaling pathway	MAPK1/MAPK14/JUN	0.000078655
hsa04010	MAPK signaling pathway	CHUK/MAPK1/MAPK14/JUN	0.000105958
hsa04066	HIF-1 signaling pathway	IFNG/MAPK1/CDKN1A	0.000126302
hsa04722	Neurotrophin signaling pathway	MAPK1/MAPK14/JUN	0.000163970
hsa04926	Relaxin signaling pathway	MAPK1/MAPK14/JUN	0.000208317
hsa04151	PI3K-akt signaling pathway	CHUK/MAPK1/CDKN1A/CCND1	0.000218113
hsa04630	JAK-STAT signaling pathway	IFNG/CDKN1A/CCND1	0.000408207
hsa04370	VEGF signaling pathway	MAPK1/MAPK14	0.001419283
hsa04664	Fc epsilon RI signaling pathway	MAPK1/MAPK14	0.001881216
hsa04622	RIG-I-like receptor signaling pathway	CHUK/MAPK14	0.001992380
hsa04115	p53 signaling pathway	CDKN1A/CCND1	0.002164888
hsa04350	TGF-beta signaling pathway	IFNG/MAPK1	0.003563485
hsa04071	Sphingolipid signaling pathway	MAPK1/MAPK14	0.005653447
hsa04919	Thyroid hormone signaling pathway	MAPK1/CCND1	0.005840102
hsa04371	Apelin signaling pathway	MAPK1/CCND1	0.007434779
hsa04915	Estrogen signaling pathway	MAPK1/JUN	0.007540373
hsa04550	Signaling pathways regulating pluripotency of stem cells	MAPK1/MAPK14	0.008078683
hsa04150	mTOR signaling pathway	CHUK/MAPK1	0.009440243
hsa04310	Wnt signaling pathway	CCND1/JUN	0.010773382
hsa04062	Chemokine signaling pathway	CHUK/MAPK1	0.014239283
hsa04015	Rap1 signaling pathway	MAPK1/MAPK14	0.016890221
hsa04024	cAMP signaling pathway	MAPK1/JUN	0.017818328
hsa04014	Ras signaling pathway	CHUK/MAPK1	0.020399537

## Data Availability

The datasets used and analyzed during the current study are available from the first author on reasonable request.

## Supplementary Materials

Supplementary Materials
*Supplementary Table 1*. LncRNA-miRNA-mRNA interactions. *Supplementary Table 2.* The drugs, active compounds, and targets of ZGP. *Supplementary Table 3*. Detailed network topology information of intersection targets.
