# Supplementary material for: Zuogui Pill Ameliorates Glucocorticoid-Induced Osteoporosis through ZNF702P-Based ceRNA Network: Bioinformatics Analysis and Experimental Validation
Source: Evid Based Complement Alternat Med. 2022 Aug 29;2022:8020182. doi: 10.1155/2022/8020182 (PMC11401717; doi:10.1155/2022/8020182)
Supplement: Supplementary Materials — Supplementary Table 1. LncRNA-miRNA-mRNA interactions. Supplementary Table 2. The drugs, active compounds, and targets of ZGP. Supplementary Table 3. Detailed network topology information of intersection targets. [file 8020182.f1.zip › 8020182.f1/Supplementary Table 1 lncRNA-miRNA-mRNA interactions.docx]

Supplementary Table 1. lncRNA-miRNA-mRNA interactions

| 75 ZNF702P-miRNA interactions | | |
| --- | --- | --- |
| hsa-miR-132 | hsa-miR-427 | hsa-miR-143 |
| hsa-miR-212 | hsa-miR-518a-3p | hsa-miR-1721 |
| hsa-miR-212-3p | hsa-miR-519d | hsa-miR-4770 |
| hsa-miR-7 | hsa-miR-181abcd | hsa-miR-146ac |
| hsa-miR-7ab | hsa-miR-4262 | hsa-miR-146b-5p |
| hsa-miR-9 | hsa-miR-18ab | hsa-miR-150 |
| hsa-miR-9ab | hsa-miR-4735-3p | hsa-miR-5127 |
| hsa-miR-93 | hsa-miR-194 | hsa-miR-153 |
| hsa-miR-93a | hsa-miR-199ab-5p | hsa-miR-17 |
| hsa-miR-105 | hsa-miR-200bc | hsa-miR-17-5p |
| hsa-miR-106a | hsa-miR-429 | hsa-miR-20ab |
| hsa-miR-291a-3p | hsa-miR-548a | hsa-miR-20b-5p |
| hsa-miR-294 | hsa-miR-203 | hsa-miR-106ab |
| hsa-miR-295 | hsa-miR-204 | hsa-miR-24ab |
| hsa-miR-302abcde | hsa-miR-204b | hsa-miR-24-3p |
| hsa-miR-372 | hsa-miR-211 | hsa-miR-29abcd |
| hsa-miR-373 | hsa-miR-216a | hsa-miR-338 |
| hsa-miR-428 | hsa-miR-216b | hsa-miR-338-3p |
| hsa-miR-519a | hsa-miR-216b-5p | hsa-miR-33ab |
| hsa-miR-520be | hsa-miR-218 | hsa-miR-33-5p |
| hsa-miR-520acd-3p | hsa-miR-218a | hsa-miR-425 |
| hsa-miR-1378 | hsa-miR-223 | hsa-miR-425-5p |
| hsa-miR-1420ac | hsa-miR-23abc | hsa-miR-489 |
| hsa-miR-141 | hsa-miR-23b-3p | hsa-miR-129-5p |
| hsa-miR-200a | hsa-miR-24 | hsa-miR-129ab-5p |

| 936 miRNA-mRNA interactions | | | | | |
| --- | --- | --- | --- | --- | --- |
| miRNA | Gene | miRDB | miRTarBase | TargetScan | Sum |
| hsa-miR-17-5p | MAP3K3 | 1 | 1 | 1 | 3 |
| hsa-miR-23b-3p | HMGB2 | 1 | 1 | 1 | 3 |
| hsa-miR-20b-5p | PFKP | 1 | 1 | 1 | 3 |
| hsa-miR-17-5p | HMBOX1 | 1 | 1 | 1 | 3 |
| hsa-miR-20b-5p | PIP4K2A | 1 | 1 | 1 | 3 |
| hsa-miR-17-5p | GNB5 | 1 | 1 | 1 | 3 |
| hsa-miR-20b-5p | TMEM123 | 1 | 1 | 1 | 3 |
| hsa-miR-429 | CAB39 | 1 | 1 | 1 | 3 |
| hsa-miR-17-5p | RAPGEF4 | 1 | 1 | 1 | 3 |
| hsa-miR-129-5p | UNC5D | 1 | 1 | 1 | 3 |
| hsa-miR-24-3p | SSR1 | 1 | 1 | 1 | 3 |
| hsa-miR-23b-3p | MCFD2 | 1 | 1 | 1 | 3 |
| hsa-miR-20b-5p | PXK | 1 | 1 | 1 | 3 |
| hsa-miR-20b-5p | FCHO2 | 1 | 1 | 1 | 3 |
| hsa-miR-23b-3p | PTEN | 1 | 1 | 1 | 3 |
| hsa-miR-20b-5p | MTMR3 | 1 | 1 | 1 | 3 |
| hsa-miR-20b-5p | FAM129A | 1 | 1 | 1 | 3 |
| hsa-miR-20b-5p | CERCAM | 1 | 1 | 1 | 3 |
| hsa-miR-23b-3p | LBR | 1 | 1 | 1 | 3 |
| hsa-miR-17-5p | TMEM138 | 1 | 1 | 1 | 3 |
| hsa-miR-17-5p | MASTL | 1 | 1 | 1 | 3 |
| hsa-miR-129-5p | SORBS2 | 1 | 1 | 1 | 3 |
| hsa-miR-20b-5p | BAMBI | 1 | 1 | 1 | 3 |
| hsa-miR-17-5p | RAB5B | 1 | 1 | 1 | 3 |
| hsa-miR-17-5p | ABHD2 | 1 | 1 | 1 | 3 |
| hsa-miR-24-3p | C15orf39 | 1 | 1 | 1 | 3 |
| hsa-miR-216b-5p | AKIP1 | 1 | 1 | 1 | 3 |
| hsa-miR-24-3p | BCL7A | 1 | 1 | 1 | 3 |
| hsa-miR-17-5p | NRIP3 | 1 | 1 | 1 | 3 |
| hsa-miR-129-5p | GNAQ | 1 | 1 | 1 | 3 |
| hsa-miR-24-3p | AMOTL2 | 1 | 1 | 1 | 3 |
| hsa-miR-20b-5p | SAMD12 | 1 | 1 | 1 | 3 |
| hsa-miR-20b-5p | LAPTM4A | 1 | 1 | 1 | 3 |
| hsa-miR-20b-5p | ATAD2 | 1 | 1 | 1 | 3 |
| hsa-miR-17-5p | MLXIP | 1 | 1 | 1 | 3 |
| hsa-miR-17-5p | GINS4 | 1 | 1 | 1 | 3 |
| hsa-miR-23b-3p | TNPO1 | 1 | 1 | 1 | 3 |
| hsa-miR-212-3p | ASF1A | 1 | 1 | 1 | 3 |
| hsa-miR-17-5p | CEP57 | 1 | 1 | 1 | 3 |
| hsa-miR-429 | ZMAT3 | 1 | 1 | 1 | 3 |
| hsa-miR-17-5p | E2F1 | 1 | 1 | 1 | 3 |
| hsa-miR-17-5p | ZFYVE9 | 1 | 1 | 1 | 3 |
| hsa-miR-17-5p | NPAT | 1 | 1 | 1 | 3 |
| hsa-miR-17-5p | UBE2Q2 | 1 | 1 | 1 | 3 |
| hsa-miR-17-5p | SLAIN2 | 1 | 1 | 1 | 3 |
| hsa-miR-17-5p | PPP1R3B | 1 | 1 | 1 | 3 |
| hsa-miR-17-5p | MIDN | 1 | 1 | 1 | 3 |
| hsa-miR-24-3p | AVL9 | 1 | 1 | 1 | 3 |
| hsa-miR-20b-5p | PPP1R15B | 1 | 1 | 1 | 3 |
| hsa-miR-23b-3p | CHUK | 1 | 1 | 1 | 3 |
| hsa-miR-129-5p | FNIP2 | 1 | 1 | 1 | 3 |
| hsa-miR-17-5p | LYSMD3 | 1 | 1 | 1 | 3 |
| hsa-miR-17-5p | ULK1 | 1 | 1 | 1 | 3 |
| hsa-miR-23b-3p | C8orf58 | 1 | 1 | 1 | 3 |
| hsa-miR-20b-5p | HMGB3 | 1 | 1 | 1 | 3 |
| hsa-miR-212-3p | PHF20L1 | 1 | 1 | 1 | 3 |
| hsa-miR-24-3p | TAOK1 | 1 | 1 | 1 | 3 |
| hsa-miR-20b-5p | CMPK1 | 1 | 1 | 1 | 3 |
| hsa-miR-129-5p | GALNT1 | 1 | 1 | 1 | 3 |
| hsa-miR-20b-5p | TRIP10 | 1 | 1 | 1 | 3 |
| hsa-miR-20b-5p | RHOC | 1 | 1 | 1 | 3 |
| hsa-miR-429 | SEC24A | 1 | 1 | 1 | 3 |
| hsa-miR-20b-5p | CHIC1 | 1 | 1 | 1 | 3 |
| hsa-miR-17-5p | ITGB8 | 1 | 1 | 1 | 3 |
| hsa-miR-20b-5p | GIGYF1 | 1 | 1 | 1 | 3 |
| hsa-miR-17-5p | MSMO1 | 1 | 1 | 1 | 3 |
| hsa-miR-129-5p | RBMXL1 | 1 | 1 | 1 | 3 |
| hsa-miR-20b-5p | CNOT6L | 1 | 1 | 1 | 3 |
| hsa-miR-17-5p | NRBP1 | 1 | 1 | 1 | 3 |
| hsa-miR-429 | GPATCH8 | 1 | 1 | 1 | 3 |
| hsa-miR-17-5p | TMEM123 | 1 | 1 | 1 | 3 |
| hsa-miR-17-5p | DENND5B | 1 | 1 | 1 | 3 |
| hsa-miR-17-5p | ANKRD52 | 1 | 1 | 1 | 3 |
| hsa-miR-425-5p | OCRL | 1 | 1 | 1 | 3 |
| hsa-miR-17-5p | KLF10 | 1 | 1 | 1 | 3 |
| hsa-miR-23b-3p | ANKRD17 | 1 | 1 | 1 | 3 |
| hsa-miR-429 | NRBP1 | 1 | 1 | 1 | 3 |
| hsa-miR-23b-3p | QSER1 | 1 | 1 | 1 | 3 |
| hsa-miR-24-3p | TRPM6 | 1 | 1 | 1 | 3 |
| hsa-miR-17-5p | RB1 | 1 | 1 | 1 | 3 |
| hsa-miR-17-5p | FAM129A | 1 | 1 | 1 | 3 |
| hsa-miR-17-5p | ARHGAP1 | 1 | 1 | 1 | 3 |
| hsa-miR-338-3p | PFAS | 1 | 1 | 1 | 3 |
| hsa-miR-20b-5p | DNAJB9 | 1 | 1 | 1 | 3 |
| hsa-miR-17-5p | RRAGD | 1 | 1 | 1 | 3 |
| hsa-miR-20b-5p | TSG101 | 1 | 1 | 1 | 3 |
| hsa-miR-20b-5p | GNB5 | 1 | 1 | 1 | 3 |
| hsa-miR-23b-3p | ZCCHC2 | 1 | 1 | 1 | 3 |
| hsa-miR-17-5p | HMGB3 | 1 | 1 | 1 | 3 |
| hsa-miR-20b-5p | CCSER2 | 1 | 1 | 1 | 3 |
| hsa-miR-23b-3p | ZEB1 | 1 | 1 | 1 | 3 |
| hsa-miR-17-5p | STK11 | 1 | 1 | 1 | 3 |
| hsa-miR-20b-5p | OXR1 | 1 | 1 | 1 | 3 |
| hsa-miR-17-5p | CFL2 | 1 | 1 | 1 | 3 |
| hsa-miR-17-5p | MMP2 | 1 | 1 | 1 | 3 |
| hsa-miR-429 | PRRG4 | 1 | 1 | 1 | 3 |
| hsa-miR-24-3p | PTGFRN | 1 | 1 | 1 | 3 |
| hsa-miR-20b-5p | TP53INP1 | 1 | 1 | 1 | 3 |
| hsa-miR-23b-3p | BTLA | 1 | 1 | 1 | 3 |
| hsa-miR-17-5p | HAUS8 | 1 | 1 | 1 | 3 |
| hsa-miR-20b-5p | UBE2Q2 | 1 | 1 | 1 | 3 |
| hsa-miR-17-5p | PDLIM5 | 1 | 1 | 1 | 3 |
| hsa-miR-17-5p | GNS | 1 | 1 | 1 | 3 |
| hsa-miR-17-5p | TXNIP | 1 | 1 | 1 | 3 |
| hsa-miR-20b-5p | EIF5A2 | 1 | 1 | 1 | 3 |
| hsa-miR-24-3p | CRIPT | 1 | 1 | 1 | 3 |
| hsa-miR-429 | ZFPM2 | 1 | 1 | 1 | 3 |
| hsa-miR-429 | CAMSAP2 | 1 | 1 | 1 | 3 |
| hsa-miR-20b-5p | BRMS1L | 1 | 1 | 1 | 3 |
| hsa-miR-20b-5p | NRIP3 | 1 | 1 | 1 | 3 |
| hsa-miR-20b-5p | SPOPL | 1 | 1 | 1 | 3 |
| hsa-miR-20b-5p | ANKRD12 | 1 | 1 | 1 | 3 |
| hsa-miR-20b-5p | ABCA1 | 1 | 1 | 1 | 3 |
| hsa-miR-17-5p | TBC1D15 | 1 | 1 | 1 | 3 |
| hsa-miR-17-5p | OXR1 | 1 | 1 | 1 | 3 |
| hsa-miR-17-5p | CEP97 | 1 | 1 | 1 | 3 |
| hsa-miR-17-5p | EIF4H | 1 | 1 | 1 | 3 |
| hsa-miR-146b-5p | IRAK1 | 1 | 1 | 1 | 3 |
| hsa-miR-17-5p | LASP1 | 1 | 1 | 1 | 3 |
| hsa-miR-17-5p | NFAT5 | 1 | 1 | 1 | 3 |
| hsa-miR-17-5p | USP3 | 1 | 1 | 1 | 3 |
| hsa-miR-23b-3p | STAT5B | 1 | 1 | 1 | 3 |
| hsa-miR-129-5p | ADD3 | 1 | 1 | 1 | 3 |
| hsa-miR-17-5p | PPP6R3 | 1 | 1 | 1 | 3 |
| hsa-miR-17-5p | IFNAR1 | 1 | 1 | 1 | 3 |
| hsa-miR-17-5p | KIAA0922 | 1 | 1 | 1 | 3 |
| hsa-miR-129-5p | DIS3 | 1 | 1 | 1 | 3 |
| hsa-miR-17-5p | KIAA0513 | 1 | 1 | 1 | 3 |
| hsa-miR-17-5p | TNKS2 | 1 | 1 | 1 | 3 |
| hsa-miR-17-5p | SCAMP5 | 1 | 1 | 1 | 3 |
| hsa-miR-17-5p | U2SURP | 1 | 1 | 1 | 3 |
| hsa-miR-338-3p | PREX2 | 1 | 1 | 1 | 3 |
| hsa-miR-23b-3p | RPRD2 | 1 | 1 | 1 | 3 |
| hsa-miR-24-3p | PER2 | 1 | 1 | 1 | 3 |
| hsa-miR-17-5p | ZNF800 | 1 | 1 | 1 | 3 |
| hsa-miR-216b-5p | TPM3 | 1 | 1 | 1 | 3 |
| hsa-miR-24-3p | NDST1 | 1 | 1 | 1 | 3 |
| hsa-miR-20b-5p | PTPN4 | 1 | 1 | 1 | 3 |
| hsa-miR-129-5p | SBNO1 | 1 | 1 | 1 | 3 |
| hsa-miR-24-3p | ADD1 | 1 | 1 | 1 | 3 |
| hsa-miR-17-5p | ANKRD50 | 1 | 1 | 1 | 3 |
| hsa-miR-24-3p | IFNG | 1 | 1 | 1 | 3 |
| hsa-miR-429 | DNAJB6 | 1 | 1 | 1 | 3 |
| hsa-miR-17-5p | RORA | 1 | 1 | 1 | 3 |
| hsa-miR-17-5p | SIK1 | 1 | 1 | 1 | 3 |
| hsa-miR-216b-5p | MCM4 | 1 | 1 | 1 | 3 |
| hsa-miR-17-5p | FBXO28 | 1 | 1 | 1 | 3 |
| hsa-miR-17-5p | AKAP11 | 1 | 1 | 1 | 3 |
| hsa-miR-17-5p | SAMD12 | 1 | 1 | 1 | 3 |
| hsa-miR-20b-5p | SLAIN2 | 1 | 1 | 1 | 3 |
| hsa-miR-129-5p | KLHL5 | 1 | 1 | 1 | 3 |
| hsa-miR-20b-5p | CLOCK | 1 | 1 | 1 | 3 |
| hsa-miR-17-5p | STAT3 | 1 | 1 | 1 | 3 |
| hsa-miR-20b-5p | CENPQ | 1 | 1 | 1 | 3 |
| hsa-miR-20b-5p | DUSP2 | 1 | 1 | 1 | 3 |
| hsa-miR-17-5p | FCHO2 | 1 | 1 | 1 | 3 |
| hsa-miR-20b-5p | PIP4K2C | 1 | 1 | 1 | 3 |
| hsa-miR-20b-5p | PTGFRN | 1 | 1 | 1 | 3 |
| hsa-miR-17-5p | LIMA1 | 1 | 1 | 1 | 3 |
| hsa-miR-20b-5p | FBXL5 | 1 | 1 | 1 | 3 |
| hsa-miR-24-3p | ZNF217 | 1 | 1 | 1 | 3 |
| hsa-miR-17-5p | GOLGA1 | 1 | 1 | 1 | 3 |
| hsa-miR-17-5p | ZBTB4 | 1 | 1 | 1 | 3 |
| hsa-miR-20b-5p | NAGK | 1 | 1 | 1 | 3 |
| hsa-miR-17-5p | IRAK4 | 1 | 1 | 1 | 3 |
| hsa-miR-17-5p | CCSER2 | 1 | 1 | 1 | 3 |
| hsa-miR-20b-5p | TANC1 | 1 | 1 | 1 | 3 |
| hsa-miR-20b-5p | MASTL | 1 | 1 | 1 | 3 |
| hsa-miR-129-5p | RBM47 | 1 | 1 | 1 | 3 |
| hsa-miR-216b-5p | ZDHHC9 | 1 | 1 | 1 | 3 |
| hsa-miR-20b-5p | LIMK1 | 1 | 1 | 1 | 3 |
| hsa-miR-17-5p | SEMA4B | 1 | 1 | 1 | 3 |
| hsa-miR-17-5p | SMAD5 | 1 | 1 | 1 | 3 |
| hsa-miR-17-5p | KIAA1147 | 1 | 1 | 1 | 3 |
| hsa-miR-20b-5p | FAM210A | 1 | 1 | 1 | 3 |
| hsa-miR-17-5p | OCRL | 1 | 1 | 1 | 3 |
| hsa-miR-20b-5p | EPHA4 | 1 | 1 | 1 | 3 |
| hsa-miR-20b-5p | CRK | 1 | 1 | 1 | 3 |
| hsa-miR-20b-5p | LPGAT1 | 1 | 1 | 1 | 3 |
| hsa-miR-429 | NUFIP2 | 1 | 1 | 1 | 3 |
| hsa-miR-20b-5p | ITCH | 1 | 1 | 1 | 3 |
| hsa-miR-17-5p | TXLNA | 1 | 1 | 1 | 3 |
| hsa-miR-17-5p | CENPQ | 1 | 1 | 1 | 3 |
| hsa-miR-17-5p | CIT | 1 | 1 | 1 | 3 |
| hsa-miR-212-3p | RB1 | 1 | 1 | 1 | 3 |
| hsa-miR-20b-5p | KLHL28 | 1 | 1 | 1 | 3 |
| hsa-miR-20b-5p | ARAP2 | 1 | 1 | 1 | 3 |
| hsa-miR-20b-5p | SLC22A23 | 1 | 1 | 1 | 3 |
| hsa-miR-17-5p | FBXO48 | 1 | 1 | 1 | 3 |
| hsa-miR-17-5p | SUCO | 1 | 1 | 1 | 3 |
| hsa-miR-129-5p | KAT6B | 1 | 1 | 1 | 3 |
| hsa-miR-129-5p | ACSL4 | 1 | 1 | 1 | 3 |
| hsa-miR-20b-5p | BTG3 | 1 | 1 | 1 | 3 |
| hsa-miR-129-5p | GABBR2 | 1 | 1 | 1 | 3 |
| hsa-miR-17-5p | RBL1 | 1 | 1 | 1 | 3 |
| hsa-miR-129-5p | APC | 1 | 1 | 1 | 3 |
| hsa-miR-17-5p | PPP6C | 1 | 1 | 1 | 3 |
| hsa-miR-129-5p | RNF165 | 1 | 1 | 1 | 3 |
| hsa-miR-20b-5p | ATG14 | 1 | 1 | 1 | 3 |
| hsa-miR-17-5p | CAMTA1 | 1 | 1 | 1 | 3 |
| hsa-miR-17-5p | MFSD8 | 1 | 1 | 1 | 3 |
| hsa-miR-20b-5p | ABHD2 | 1 | 1 | 1 | 3 |
| hsa-miR-24-3p | HIC2 | 1 | 1 | 1 | 3 |
| hsa-miR-17-5p | TMEM127 | 1 | 1 | 1 | 3 |
| hsa-miR-17-5p | RUNX3 | 1 | 1 | 1 | 3 |
| hsa-miR-20b-5p | EIF4H | 1 | 1 | 1 | 3 |
| hsa-miR-17-5p | PLEKHM1 | 1 | 1 | 1 | 3 |
| hsa-miR-23b-3p | ETNK1 | 1 | 1 | 1 | 3 |
| hsa-miR-24-3p | AP5M1 | 1 | 1 | 1 | 3 |
| hsa-miR-20b-5p | RPA2 | 1 | 1 | 1 | 3 |
| hsa-miR-429 | WASF3 | 1 | 1 | 1 | 3 |
| hsa-miR-17-5p | MKNK2 | 1 | 1 | 1 | 3 |
| hsa-miR-17-5p | PTP4A1 | 1 | 1 | 1 | 3 |
| hsa-miR-24-3p | POGZ | 1 | 1 | 1 | 3 |
| hsa-miR-17-5p | BTN3A1 | 1 | 1 | 1 | 3 |
| hsa-miR-20b-5p | MCC | 1 | 1 | 1 | 3 |
| hsa-miR-24-3p | BCL2L11 | 1 | 1 | 1 | 3 |
| hsa-miR-20b-5p | KIAA0922 | 1 | 1 | 1 | 3 |
| hsa-miR-129-5p | USP6NL | 1 | 1 | 1 | 3 |
| hsa-miR-429 | NCOA2 | 1 | 1 | 1 | 3 |
| hsa-miR-17-5p | MCL1 | 1 | 1 | 1 | 3 |
| hsa-miR-20b-5p | PPP1R3B | 1 | 1 | 1 | 3 |
| hsa-miR-20b-5p | CCL1 | 1 | 1 | 1 | 3 |
| hsa-miR-129-5p | REEP1 | 1 | 1 | 1 | 3 |
| hsa-miR-17-5p | MAPK9 | 1 | 1 | 1 | 3 |
| hsa-miR-20b-5p | ELAVL2 | 1 | 1 | 1 | 3 |
| hsa-miR-20b-5p | WAC | 1 | 1 | 1 | 3 |
| hsa-miR-23b-3p | GHITM | 1 | 1 | 1 | 3 |
| hsa-miR-17-5p | ENPP5 | 1 | 1 | 1 | 3 |
| hsa-miR-429 | PSD3 | 1 | 1 | 1 | 3 |
| hsa-miR-20b-5p | C7orf43 | 1 | 1 | 1 | 3 |
| hsa-miR-425-5p | AFF4 | 1 | 1 | 1 | 3 |
| hsa-miR-24-3p | MATR3 | 1 | 1 | 1 | 3 |
| hsa-miR-425-5p | BCOR | 1 | 1 | 1 | 3 |
| hsa-miR-24-3p | POLR3D | 1 | 1 | 1 | 3 |
| hsa-miR-24-3p | FURIN | 1 | 1 | 1 | 3 |
| hsa-miR-24-3p | C17orf49 | 1 | 1 | 1 | 3 |
| hsa-miR-17-5p | SLK | 1 | 1 | 1 | 3 |
| hsa-miR-23b-3p | RRAS2 | 1 | 1 | 1 | 3 |
| hsa-miR-20b-5p | PHF6 | 1 | 1 | 1 | 3 |
| hsa-miR-216b-5p | PPP2CB | 1 | 1 | 1 | 3 |
| hsa-miR-24-3p | MBD6 | 1 | 1 | 1 | 3 |
| hsa-miR-20b-5p | CRY2 | 1 | 1 | 1 | 3 |
| hsa-miR-429 | PDPK1 | 1 | 1 | 1 | 3 |
| hsa-miR-24-3p | TSC22D2 | 1 | 1 | 1 | 3 |
| hsa-miR-17-5p | PLS1 | 1 | 1 | 1 | 3 |
| hsa-miR-17-5p | PIP4K2C | 1 | 1 | 1 | 3 |
| hsa-miR-17-5p | OSTM1 | 1 | 1 | 1 | 3 |
| hsa-miR-20b-5p | HAS2 | 1 | 1 | 1 | 3 |
| hsa-miR-20b-5p | STK17B | 1 | 1 | 1 | 3 |
| hsa-miR-20b-5p | CEP97 | 1 | 1 | 1 | 3 |
| hsa-miR-24-3p | PIM2 | 1 | 1 | 1 | 3 |
| hsa-miR-20b-5p | PLXNA1 | 1 | 1 | 1 | 3 |
| hsa-miR-17-5p | FRS2 | 1 | 1 | 1 | 3 |
| hsa-miR-24-3p | PTPN9 | 1 | 1 | 1 | 3 |
| hsa-miR-20b-5p | FOXJ3 | 1 | 1 | 1 | 3 |
| hsa-miR-17-5p | MAP3K9 | 1 | 1 | 1 | 3 |
| hsa-miR-17-5p | ORMDL3 | 1 | 1 | 1 | 3 |
| hsa-miR-20b-5p | PANK3 | 1 | 1 | 1 | 3 |
| hsa-miR-20b-5p | POLQ | 1 | 1 | 1 | 3 |
| hsa-miR-17-5p | ZBTB18 | 1 | 1 | 1 | 3 |
| hsa-miR-23b-3p | PPP1CB | 1 | 1 | 1 | 3 |
| hsa-miR-20b-5p | MAPK1 | 1 | 1 | 1 | 3 |
| hsa-miR-17-5p | SNTB2 | 1 | 1 | 1 | 3 |
| hsa-miR-20b-5p | RORA | 1 | 1 | 1 | 3 |
| hsa-miR-17-5p | BMPR2 | 1 | 1 | 1 | 3 |
| hsa-miR-17-5p | TMEM64 | 1 | 1 | 1 | 3 |
| hsa-miR-20b-5p | USP3 | 1 | 1 | 1 | 3 |
| hsa-miR-129-5p | TERF2 | 1 | 1 | 1 | 3 |
| hsa-miR-17-5p | CAPN15 | 1 | 1 | 1 | 3 |
| hsa-miR-20b-5p | FBXO21 | 1 | 1 | 1 | 3 |
| hsa-miR-17-5p | TMEM242 | 1 | 1 | 1 | 3 |
| hsa-miR-17-5p | C7orf43 | 1 | 1 | 1 | 3 |
| hsa-miR-129-5p | RYBP | 1 | 1 | 1 | 3 |
| hsa-miR-17-5p | ATAD2 | 1 | 1 | 1 | 3 |
| hsa-miR-20b-5p | FYCO1 | 1 | 1 | 1 | 3 |
| hsa-miR-20b-5p | SCAMP5 | 1 | 1 | 1 | 3 |
| hsa-miR-24-3p | RNF11 | 1 | 1 | 1 | 3 |
| hsa-miR-17-5p | RCCD1 | 1 | 1 | 1 | 3 |
| hsa-miR-20b-5p | PPP6R3 | 1 | 1 | 1 | 3 |
| hsa-miR-24-3p | CD34 | 1 | 1 | 1 | 3 |
| hsa-miR-17-5p | ZNF202 | 1 | 1 | 1 | 3 |
| hsa-miR-24-3p | TMEM173 | 1 | 1 | 1 | 3 |
| hsa-miR-24-3p | ACVR1B | 1 | 1 | 1 | 3 |
| hsa-miR-17-5p | RPA2 | 1 | 1 | 1 | 3 |
| hsa-miR-17-5p | NKIRAS1 | 1 | 1 | 1 | 3 |
| hsa-miR-20b-5p | GNS | 1 | 1 | 1 | 3 |
| hsa-miR-24-3p | GBA2 | 1 | 1 | 1 | 3 |
| hsa-miR-23b-3p | ATXN7L3B | 1 | 1 | 1 | 3 |
| hsa-miR-24-3p | MLEC | 1 | 1 | 1 | 3 |
| hsa-miR-17-5p | TSG101 | 1 | 1 | 1 | 3 |
| hsa-miR-17-5p | UBXN2A | 1 | 1 | 1 | 3 |
| hsa-miR-17-5p | KLF3 | 1 | 1 | 1 | 3 |
| hsa-miR-17-5p | PARD6B | 1 | 1 | 1 | 3 |
| hsa-miR-17-5p | RLIM | 1 | 1 | 1 | 3 |
| hsa-miR-17-5p | SRSF2 | 1 | 1 | 1 | 3 |
| hsa-miR-129-5p | LIMS1 | 1 | 1 | 1 | 3 |
| hsa-miR-20b-5p | RUFY2 | 1 | 1 | 1 | 3 |
| hsa-miR-17-5p | FOXJ2 | 1 | 1 | 1 | 3 |
| hsa-miR-24-3p | LIMD1 | 1 | 1 | 1 | 3 |
| hsa-miR-425-5p | EOGT | 1 | 1 | 1 | 3 |
| hsa-miR-17-5p | BBX | 1 | 1 | 1 | 3 |
| hsa-miR-17-5p | NCOA3 | 1 | 1 | 1 | 3 |
| hsa-miR-17-5p | WAC | 1 | 1 | 1 | 3 |
| hsa-miR-20b-5p | SMOC1 | 1 | 1 | 1 | 3 |
| hsa-miR-20b-5p | ATG16L1 | 1 | 1 | 1 | 3 |
| hsa-miR-17-5p | FAM57A | 1 | 1 | 1 | 3 |
| hsa-miR-20b-5p | GOLGA1 | 1 | 1 | 1 | 3 |
| hsa-miR-17-5p | SOCS6 | 1 | 1 | 1 | 3 |
| hsa-miR-17-5p | GPAM | 1 | 1 | 1 | 3 |
| hsa-miR-17-5p | UBE3C | 1 | 1 | 1 | 3 |
| hsa-miR-17-5p | RAB22A | 1 | 1 | 1 | 3 |
| hsa-miR-20b-5p | BTBD7 | 1 | 1 | 1 | 3 |
| hsa-miR-20b-5p | ANKRD33B | 1 | 1 | 1 | 3 |
| hsa-miR-17-5p | CHAF1A | 1 | 1 | 1 | 3 |
| hsa-miR-17-5p | YOD1 | 1 | 1 | 1 | 3 |
| hsa-miR-17-5p | REEP3 | 1 | 1 | 1 | 3 |
| hsa-miR-17-5p | TMBIM6 | 1 | 1 | 1 | 3 |
| hsa-miR-20b-5p | NUP35 | 1 | 1 | 1 | 3 |
| hsa-miR-17-5p | EIF4G2 | 1 | 1 | 1 | 3 |
| hsa-miR-23b-3p | NLGN4X | 1 | 1 | 1 | 3 |
| hsa-miR-20b-5p | CFL2 | 1 | 1 | 1 | 3 |
| hsa-miR-212-3p | ARID2 | 1 | 1 | 1 | 3 |
| hsa-miR-17-5p | DYNC1LI2 | 1 | 1 | 1 | 3 |
| hsa-miR-20b-5p | ZFYVE26 | 1 | 1 | 1 | 3 |
| hsa-miR-129-5p | SLBP | 1 | 1 | 1 | 3 |
| hsa-miR-129-5p | SEMA6A | 1 | 1 | 1 | 3 |
| hsa-miR-17-5p | ANKFY1 | 1 | 1 | 1 | 3 |
| hsa-miR-17-5p | PKD2 | 1 | 1 | 1 | 3 |
| hsa-miR-20b-5p | HBP1 | 1 | 1 | 1 | 3 |
| hsa-miR-17-5p | POLR3G | 1 | 1 | 1 | 3 |
| hsa-miR-17-5p | MAP3K8 | 1 | 1 | 1 | 3 |
| hsa-miR-24-3p | MEN1 | 1 | 1 | 1 | 3 |
| hsa-miR-20b-5p | ANKH | 1 | 1 | 1 | 3 |
| hsa-miR-20b-5p | MINK1 | 1 | 1 | 1 | 3 |
| hsa-miR-20b-5p | TXNIP | 1 | 1 | 1 | 3 |
| hsa-miR-17-5p | ERAP1 | 1 | 1 | 1 | 3 |
| hsa-miR-20b-5p | PARD6B | 1 | 1 | 1 | 3 |
| hsa-miR-20b-5p | MKNK2 | 1 | 1 | 1 | 3 |
| hsa-miR-20b-5p | WDR37 | 1 | 1 | 1 | 3 |
| hsa-miR-17-5p | EIF5A2 | 1 | 1 | 1 | 3 |
| hsa-miR-20b-5p | PLEKHO2 | 1 | 1 | 1 | 3 |
| hsa-miR-429 | RASSF8 | 1 | 1 | 1 | 3 |
| hsa-miR-20b-5p | KIAA0513 | 1 | 1 | 1 | 3 |
| hsa-miR-17-5p | SERF1B | 1 | 1 | 1 | 3 |
| hsa-miR-24-3p | EMP2 | 1 | 1 | 1 | 3 |
| hsa-miR-17-5p | MINK1 | 1 | 1 | 1 | 3 |
| hsa-miR-20b-5p | HIF1A | 1 | 1 | 1 | 3 |
| hsa-miR-24-3p | PLIN3 | 1 | 1 | 1 | 3 |
| hsa-miR-17-5p | SHOC2 | 1 | 1 | 1 | 3 |
| hsa-miR-20b-5p | FOXQ1 | 1 | 1 | 1 | 3 |
| hsa-miR-20b-5p | RAB5B | 1 | 1 | 1 | 3 |
| hsa-miR-20b-5p | SLK | 1 | 1 | 1 | 3 |
| hsa-miR-17-5p | SKIL | 1 | 1 | 1 | 3 |
| hsa-miR-20b-5p | KLF10 | 1 | 1 | 1 | 3 |
| hsa-miR-20b-5p | GID4 | 1 | 1 | 1 | 3 |
| hsa-miR-17-5p | TGFBR2 | 1 | 1 | 1 | 3 |
| hsa-miR-17-5p | SQSTM1 | 1 | 1 | 1 | 3 |
| hsa-miR-17-5p | CCDC71L | 1 | 1 | 1 | 3 |
| hsa-miR-20b-5p | TADA2B | 1 | 1 | 1 | 3 |
| hsa-miR-20b-5p | CREB1 | 1 | 1 | 1 | 3 |
| hsa-miR-20b-5p | TMEM127 | 1 | 1 | 1 | 3 |
| hsa-miR-429 | CASP2 | 1 | 1 | 1 | 3 |
| hsa-miR-20b-5p | ANKIB1 | 1 | 1 | 1 | 3 |
| hsa-miR-20b-5p | UBXN2A | 1 | 1 | 1 | 3 |
| hsa-miR-20b-5p | STAT3 | 1 | 1 | 1 | 3 |
| hsa-miR-20b-5p | CDKN1A | 1 | 1 | 1 | 3 |
| hsa-miR-20b-5p | KMT2B | 1 | 1 | 1 | 3 |
| hsa-miR-24-3p | DDN | 1 | 1 | 1 | 3 |
| hsa-miR-129-5p | SOX4 | 1 | 1 | 1 | 3 |
| hsa-miR-17-5p | BTG3 | 1 | 1 | 1 | 3 |
| hsa-miR-129-5p | FMR1 | 1 | 1 | 1 | 3 |
| hsa-miR-20b-5p | RAB11FIP1 | 1 | 1 | 1 | 3 |
| hsa-miR-20b-5p | CNOT7 | 1 | 1 | 1 | 3 |
| hsa-miR-24-3p | DND1 | 1 | 1 | 1 | 3 |
| hsa-miR-20b-5p | FAM117B | 1 | 1 | 1 | 3 |
| hsa-miR-17-5p | ZNFX1 | 1 | 1 | 1 | 3 |
| hsa-miR-17-5p | DNAJB9 | 1 | 1 | 1 | 3 |
| hsa-miR-20b-5p | SIK1 | 1 | 1 | 1 | 3 |
| hsa-miR-17-5p | KIF23 | 1 | 1 | 1 | 3 |
| hsa-miR-129-5p | SESN3 | 1 | 1 | 1 | 3 |
| hsa-miR-20b-5p | ENPP5 | 1 | 1 | 1 | 3 |
| hsa-miR-17-5p | FBXO21 | 1 | 1 | 1 | 3 |
| hsa-miR-24-3p | HKR1 | 1 | 1 | 1 | 3 |
| hsa-miR-24-3p | FBLIM1 | 1 | 1 | 1 | 3 |
| hsa-miR-212-3p | AMD1 | 1 | 1 | 1 | 3 |
| hsa-miR-24-3p | ZXDA | 1 | 1 | 1 | 3 |
| hsa-miR-20b-5p | PLS1 | 1 | 1 | 1 | 3 |
| hsa-miR-24-3p | NCOA5 | 1 | 1 | 1 | 3 |
| hsa-miR-129-5p | AKAP10 | 1 | 1 | 1 | 3 |
| hsa-miR-17-5p | RNF145 | 1 | 1 | 1 | 3 |
| hsa-miR-212-3p | AGO1 | 1 | 1 | 1 | 3 |
| hsa-miR-17-5p | EGLN3 | 1 | 1 | 1 | 3 |
| hsa-miR-20b-5p | HMBOX1 | 1 | 1 | 1 | 3 |
| hsa-miR-20b-5p | ZBTB9 | 1 | 1 | 1 | 3 |
| hsa-miR-24-3p | RNF2 | 1 | 1 | 1 | 3 |
| hsa-miR-425-5p | RNF168 | 1 | 1 | 1 | 3 |
| hsa-miR-146b-5p | HNRNPD | 1 | 1 | 1 | 3 |
| hsa-miR-17-5p | POLQ | 1 | 1 | 1 | 3 |
| hsa-miR-17-5p | TADA2B | 1 | 1 | 1 | 3 |
| hsa-miR-17-5p | DNAJC27 | 1 | 1 | 1 | 3 |
| hsa-miR-20b-5p | PRR14L | 1 | 1 | 1 | 3 |
| hsa-miR-20b-5p | FEM1C | 1 | 1 | 1 | 3 |
| hsa-miR-129-5p | DUSP10 | 1 | 1 | 1 | 3 |
| hsa-miR-20b-5p | UXS1 | 1 | 1 | 1 | 3 |
| hsa-miR-17-5p | NHLRC3 | 1 | 1 | 1 | 3 |
| hsa-miR-17-5p | MTMR3 | 1 | 1 | 1 | 3 |
| hsa-miR-429 | PMAIP1 | 1 | 1 | 1 | 3 |
| hsa-miR-17-5p | TBC1D2 | 1 | 1 | 1 | 3 |
| hsa-miR-24-3p | FSCN1 | 1 | 1 | 1 | 3 |
| hsa-miR-20b-5p | ZC3H12C | 1 | 1 | 1 | 3 |
| hsa-miR-17-5p | ARID4B | 1 | 1 | 1 | 3 |
| hsa-miR-20b-5p | ZFYVE9 | 1 | 1 | 1 | 3 |
| hsa-miR-425-5p | LCOR | 1 | 1 | 1 | 3 |
| hsa-miR-17-5p | TWF1 | 1 | 1 | 1 | 3 |
| hsa-miR-17-5p | GAB1 | 1 | 1 | 1 | 3 |
| hsa-miR-216b-5p | TM9SF3 | 1 | 1 | 1 | 3 |
| hsa-miR-338-3p | SGTB | 1 | 1 | 1 | 3 |
| hsa-miR-20b-5p | CAPRIN2 | 1 | 1 | 1 | 3 |
| hsa-miR-20b-5p | OSTM1 | 1 | 1 | 1 | 3 |
| hsa-miR-17-5p | MAP3K12 | 1 | 1 | 1 | 3 |
| hsa-miR-20b-5p | CHAF1A | 1 | 1 | 1 | 3 |
| hsa-miR-24-3p | RAP2C | 1 | 1 | 1 | 3 |
| hsa-miR-17-5p | PTPN4 | 1 | 1 | 1 | 3 |
| hsa-miR-212-3p | PRDM15 | 1 | 1 | 1 | 3 |
| hsa-miR-17-5p | SSX2IP | 1 | 1 | 1 | 3 |
| hsa-miR-17-5p | MAPRE3 | 1 | 1 | 1 | 3 |
| hsa-miR-17-5p | CRK | 1 | 1 | 1 | 3 |
| hsa-miR-23b-3p | POM121C | 1 | 1 | 1 | 3 |
| hsa-miR-17-5p | VPS26A | 1 | 1 | 1 | 3 |
| hsa-miR-23b-3p | PTK2B | 1 | 1 | 1 | 3 |
| hsa-miR-17-5p | SCAMP2 | 1 | 1 | 1 | 3 |
| hsa-miR-17-5p | ZNF107 | 1 | 1 | 1 | 3 |
| hsa-miR-17-5p | CYBRD1 | 1 | 1 | 1 | 3 |
| hsa-miR-129-5p | EBF1 | 1 | 1 | 1 | 3 |
| hsa-miR-429 | MAPK7 | 1 | 1 | 1 | 3 |
| hsa-miR-20b-5p | REST | 1 | 1 | 1 | 3 |
| hsa-miR-17-5p | TNFAIP1 | 1 | 1 | 1 | 3 |
| hsa-miR-20b-5p | JAK1 | 1 | 1 | 1 | 3 |
| hsa-miR-20b-5p | BBX | 1 | 1 | 1 | 3 |
| hsa-miR-20b-5p | BMPR2 | 1 | 1 | 1 | 3 |
| hsa-miR-17-5p | PIP4K2A | 1 | 1 | 1 | 3 |
| hsa-miR-20b-5p | NKIRAS1 | 1 | 1 | 1 | 3 |
| hsa-miR-17-5p | ITCH | 1 | 1 | 1 | 3 |
| hsa-miR-20b-5p | FAM102A | 1 | 1 | 1 | 3 |
| hsa-miR-129-5p | CDK6 | 1 | 1 | 1 | 3 |
| hsa-miR-20b-5p | TWF1 | 1 | 1 | 1 | 3 |
| hsa-miR-17-5p | ZBTB7A | 1 | 1 | 1 | 3 |
| hsa-miR-425-5p | SLC16A1 | 1 | 1 | 1 | 3 |
| hsa-miR-20b-5p | NHLRC3 | 1 | 1 | 1 | 3 |
| hsa-miR-129-5p | C1S | 1 | 1 | 1 | 3 |
| hsa-miR-17-5p | NPAS2 | 1 | 1 | 1 | 3 |
| hsa-miR-20b-5p | ATL3 | 1 | 1 | 1 | 3 |
| hsa-miR-212-3p | BMPER | 1 | 1 | 1 | 3 |
| hsa-miR-17-5p | FAXC | 1 | 1 | 1 | 3 |
| hsa-miR-20b-5p | AKTIP | 1 | 1 | 1 | 3 |
| hsa-miR-24-3p | FGFR3 | 1 | 1 | 1 | 3 |
| hsa-miR-17-5p | RHOC | 1 | 1 | 1 | 3 |
| hsa-miR-20b-5p | GBF1 | 1 | 1 | 1 | 3 |
| hsa-miR-17-5p | BNIP2 | 1 | 1 | 1 | 3 |
| hsa-miR-20b-5p | ANKRD13C | 1 | 1 | 1 | 3 |
| hsa-miR-20b-5p | NR2C2 | 1 | 1 | 1 | 3 |
| hsa-miR-17-5p | PKNOX1 | 1 | 1 | 1 | 3 |
| hsa-miR-20b-5p | MAP3K3 | 1 | 1 | 1 | 3 |
| hsa-miR-17-5p | RBL2 | 1 | 1 | 1 | 3 |
| hsa-miR-20b-5p | GAB1 | 1 | 1 | 1 | 3 |
| hsa-miR-24-3p | MAP3K9 | 1 | 1 | 1 | 3 |
| hsa-miR-17-5p | ZNF532 | 1 | 1 | 1 | 3 |
| hsa-miR-17-5p | CHIC1 | 1 | 1 | 1 | 3 |
| hsa-miR-17-5p | SALL3 | 1 | 1 | 1 | 3 |
| hsa-miR-17-5p | RBBP7 | 1 | 1 | 1 | 3 |
| hsa-miR-17-5p | MORF4L1 | 1 | 1 | 1 | 3 |
| hsa-miR-20b-5p | USP32 | 1 | 1 | 1 | 3 |
| hsa-miR-129-5p | PRDM1 | 1 | 1 | 1 | 3 |
| hsa-miR-17-5p | ARAP2 | 1 | 1 | 1 | 3 |
| hsa-miR-23b-3p | SDHD | 1 | 1 | 1 | 3 |
| hsa-miR-20b-5p | DENND5B | 1 | 1 | 1 | 3 |
| hsa-miR-17-5p | SACS | 1 | 1 | 1 | 3 |
| hsa-miR-20b-5p | PTPDC1 | 1 | 1 | 1 | 3 |
| hsa-miR-20b-5p | SHOC2 | 1 | 1 | 1 | 3 |
| hsa-miR-20b-5p | CIT | 1 | 1 | 1 | 3 |
| hsa-miR-20b-5p | TMBIM6 | 1 | 1 | 1 | 3 |
| hsa-miR-24-3p | DVL3 | 1 | 1 | 1 | 3 |
| hsa-miR-17-5p | SESN3 | 1 | 1 | 1 | 3 |
| hsa-miR-17-5p | TOPORS | 1 | 1 | 1 | 3 |
| hsa-miR-23b-3p | FNIP1 | 1 | 1 | 1 | 3 |
| hsa-miR-17-5p | ANKRD33B | 1 | 1 | 1 | 3 |
| hsa-miR-129-5p | NWD1 | 1 | 1 | 1 | 3 |
| hsa-miR-17-5p | SSH2 | 1 | 1 | 1 | 3 |
| hsa-miR-17-5p | TANC1 | 1 | 1 | 1 | 3 |
| hsa-miR-17-5p | KMT2A | 1 | 1 | 1 | 3 |
| hsa-miR-129-5p | EXPH5 | 1 | 1 | 1 | 3 |
| hsa-miR-429 | PARD6B | 1 | 1 | 1 | 3 |
| hsa-miR-20b-5p | SALL3 | 1 | 1 | 1 | 3 |
| hsa-miR-17-5p | CPOX | 1 | 1 | 1 | 3 |
| hsa-miR-17-5p | KLHL28 | 1 | 1 | 1 | 3 |
| hsa-miR-17-5p | PGM2L1 | 1 | 1 | 1 | 3 |
| hsa-miR-425-5p | ARIH1 | 1 | 1 | 1 | 3 |
| hsa-miR-24-3p | STRADB | 1 | 1 | 1 | 3 |
| hsa-miR-23b-3p | TMED7 | 1 | 1 | 1 | 3 |
| hsa-miR-20b-5p | E2F5 | 1 | 1 | 1 | 3 |
| hsa-miR-20b-5p | CYBRD1 | 1 | 1 | 1 | 3 |
| hsa-miR-429 | ZEB1 | 1 | 1 | 1 | 3 |
| hsa-miR-24-3p | SCML1 | 1 | 1 | 1 | 3 |
| hsa-miR-20b-5p | TNFRSF21 | 1 | 1 | 1 | 3 |
| hsa-miR-129-5p | CALM1 | 1 | 1 | 1 | 3 |
| hsa-miR-20b-5p | EEA1 | 1 | 1 | 1 | 3 |
| hsa-miR-17-5p | PPP1R15B | 1 | 1 | 1 | 3 |
| hsa-miR-17-5p | RUNDC1 | 1 | 1 | 1 | 3 |
| hsa-miR-17-5p | NUP35 | 1 | 1 | 1 | 3 |
| hsa-miR-129-5p | STON2 | 1 | 1 | 1 | 3 |
| hsa-miR-17-5p | MAP3K2 | 1 | 1 | 1 | 3 |
| hsa-miR-129-5p | HMGB1 | 1 | 1 | 1 | 3 |
| hsa-miR-17-5p | FEM1C | 1 | 1 | 1 | 3 |
| hsa-miR-17-5p | CTSA | 1 | 1 | 1 | 3 |
| hsa-miR-17-5p | FAM46C | 1 | 1 | 1 | 3 |
| hsa-miR-20b-5p | CCDC71L | 1 | 1 | 1 | 3 |
| hsa-miR-20b-5p | KIF23 | 1 | 1 | 1 | 3 |
| hsa-miR-20b-5p | FNBP1L | 1 | 1 | 1 | 3 |
| hsa-miR-216b-5p | C11orf57 | 1 | 1 | 1 | 3 |
| hsa-miR-17-5p | TRIP10 | 1 | 1 | 1 | 3 |
| hsa-miR-20b-5p | ULK1 | 1 | 1 | 1 | 3 |
| hsa-miR-20b-5p | EGLN3 | 1 | 1 | 1 | 3 |
| hsa-miR-20b-5p | NRBP1 | 1 | 1 | 1 | 3 |
| hsa-miR-425-5p | RAB31 | 1 | 1 | 1 | 3 |
| hsa-miR-23b-3p | PNRC2 | 1 | 1 | 1 | 3 |
| hsa-miR-20b-5p | SLC16A9 | 1 | 1 | 1 | 3 |
| hsa-miR-17-5p | FOXJ3 | 1 | 1 | 1 | 3 |
| hsa-miR-17-5p | BTBD7 | 1 | 1 | 1 | 3 |
| hsa-miR-20b-5p | USP28 | 1 | 1 | 1 | 3 |
| hsa-miR-23b-3p | FUT4 | 1 | 1 | 1 | 3 |
| hsa-miR-20b-5p | KLF3 | 1 | 1 | 1 | 3 |
| hsa-miR-20b-5p | TMEM64 | 1 | 1 | 1 | 3 |
| hsa-miR-17-5p | RABEP1 | 1 | 1 | 1 | 3 |
| hsa-miR-20b-5p | ARHGAP35 | 1 | 1 | 1 | 3 |
| hsa-miR-20b-5p | NIN | 1 | 1 | 1 | 3 |
| hsa-miR-17-5p | NACC2 | 1 | 1 | 1 | 3 |
| hsa-miR-23b-3p | UQCRFS1 | 1 | 1 | 1 | 3 |
| hsa-miR-20b-5p | TMEM245 | 1 | 1 | 1 | 3 |
| hsa-miR-20b-5p | ZNFX1 | 1 | 1 | 1 | 3 |
| hsa-miR-17-5p | VPS13C | 1 | 1 | 1 | 3 |
| hsa-miR-20b-5p | SACS | 1 | 1 | 1 | 3 |
| hsa-miR-20b-5p | NFAT5 | 1 | 1 | 1 | 3 |
| hsa-miR-23b-3p | FBN2 | 1 | 1 | 1 | 3 |
| hsa-miR-20b-5p | YOD1 | 1 | 1 | 1 | 3 |
| hsa-miR-425-5p | BEX4 | 1 | 1 | 1 | 3 |
| hsa-miR-17-5p | DDX5 | 1 | 1 | 1 | 3 |
| hsa-miR-20b-5p | TRIM37 | 1 | 1 | 1 | 3 |
| hsa-miR-20b-5p | ARHGAP12 | 1 | 1 | 1 | 3 |
| hsa-miR-24-3p | YRDC | 1 | 1 | 1 | 3 |
| hsa-miR-20b-5p | FBXO48 | 1 | 1 | 1 | 3 |
| hsa-miR-17-5p | FAM210A | 1 | 1 | 1 | 3 |
| hsa-miR-24-3p | DYRK2 | 1 | 1 | 1 | 3 |
| hsa-miR-17-5p | KAT2B | 1 | 1 | 1 | 3 |
| hsa-miR-20b-5p | NABP1 | 1 | 1 | 1 | 3 |
| hsa-miR-20b-5p | ARHGAP1 | 1 | 1 | 1 | 3 |
| hsa-miR-17-5p | RPF2 | 1 | 1 | 1 | 3 |
| hsa-miR-17-5p | PDZD11 | 1 | 1 | 1 | 3 |
| hsa-miR-20b-5p | NETO2 | 1 | 1 | 1 | 3 |
| hsa-miR-20b-5p | CEP170 | 1 | 1 | 1 | 3 |
| hsa-miR-212-3p | SOD2 | 1 | 1 | 1 | 3 |
| hsa-miR-17-5p | C9orf40 | 1 | 1 | 1 | 3 |
| hsa-miR-146b-5p | TRAF6 | 1 | 1 | 1 | 3 |
| hsa-miR-17-5p | FAM102A | 1 | 1 | 1 | 3 |
| hsa-miR-216b-5p | COL4A4 | 1 | 1 | 1 | 3 |
| hsa-miR-429 | BAP1 | 1 | 1 | 1 | 3 |
| hsa-miR-17-5p | NIPA1 | 1 | 1 | 1 | 3 |
| hsa-miR-17-5p | CMPK1 | 1 | 1 | 1 | 3 |
| hsa-miR-20b-5p | DDHD1 | 1 | 1 | 1 | 3 |
| hsa-miR-20b-5p | CCND1 | 1 | 1 | 1 | 3 |
| hsa-miR-17-5p | NR2C2 | 1 | 1 | 1 | 3 |
| hsa-miR-17-5p | CERCAM | 1 | 1 | 1 | 3 |
| hsa-miR-17-5p | CAPRIN2 | 1 | 1 | 1 | 3 |
| hsa-miR-212-3p | CCDC169 | 1 | 1 | 1 | 3 |
| hsa-miR-20b-5p | EFCAB14 | 1 | 1 | 1 | 3 |
| hsa-miR-212-3p | FXR1 | 1 | 1 | 1 | 3 |
| hsa-miR-17-5p | CDKN1A | 1 | 1 | 1 | 3 |
| hsa-miR-429 | SHCBP1 | 1 | 1 | 1 | 3 |
| hsa-miR-20b-5p | DPYSL2 | 1 | 1 | 1 | 3 |
| hsa-miR-17-5p | AGO1 | 1 | 1 | 1 | 3 |
| hsa-miR-24-3p | YOD1 | 1 | 1 | 1 | 3 |
| hsa-miR-212-3p | PEA15 | 1 | 1 | 1 | 3 |
| hsa-miR-24-3p | DNAJB12 | 1 | 1 | 1 | 3 |
| hsa-miR-17-5p | RRM2 | 1 | 1 | 1 | 3 |
| hsa-miR-20b-5p | SMAD5 | 1 | 1 | 1 | 3 |
| hsa-miR-20b-5p | RUNX3 | 1 | 1 | 1 | 3 |
| hsa-miR-23b-3p | SOCS6 | 1 | 1 | 1 | 3 |
| hsa-miR-17-5p | CNOT7 | 1 | 1 | 1 | 3 |
| hsa-miR-17-5p | SIKE1 | 1 | 1 | 1 | 3 |
| hsa-miR-23b-3p | GJA1 | 1 | 1 | 1 | 3 |
| hsa-miR-24-3p | PDXK | 1 | 1 | 1 | 3 |
| hsa-miR-20b-5p | CEP57 | 1 | 1 | 1 | 3 |
| hsa-miR-24-3p | SPIN4 | 1 | 1 | 1 | 3 |
| hsa-miR-425-5p | ATP5G3 | 1 | 1 | 1 | 3 |
| hsa-miR-425-5p | BHLHB9 | 1 | 1 | 1 | 3 |
| hsa-miR-24-3p | MAPK14 | 1 | 1 | 1 | 3 |
| hsa-miR-17-5p | KATNAL1 | 1 | 1 | 1 | 3 |
| hsa-miR-20b-5p | SEMA7A | 1 | 1 | 1 | 3 |
| hsa-miR-17-5p | PANK3 | 1 | 1 | 1 | 3 |
| hsa-miR-24-3p | TOP1 | 1 | 1 | 1 | 3 |
| hsa-miR-17-5p | C14orf28 | 1 | 1 | 1 | 3 |
| hsa-miR-17-5p | LAPTM4A | 1 | 1 | 1 | 3 |
| hsa-miR-20b-5p | SEPT2 | 1 | 1 | 1 | 3 |
| hsa-miR-17-5p | PAFAH1B1 | 1 | 1 | 1 | 3 |
| hsa-miR-20b-5p | PPP3R1 | 1 | 1 | 1 | 3 |
| hsa-miR-17-5p | CNOT6L | 1 | 1 | 1 | 3 |
| hsa-miR-17-5p | TFAM | 1 | 1 | 1 | 3 |
| hsa-miR-20b-5p | RB1 | 1 | 1 | 1 | 3 |
| hsa-miR-17-5p | CEP170 | 1 | 1 | 1 | 3 |
| hsa-miR-429 | JUN | 1 | 1 | 1 | 3 |
| hsa-miR-20b-5p | ACSL4 | 1 | 1 | 1 | 3 |
| hsa-miR-23b-3p | NUFIP2 | 1 | 1 | 1 | 3 |
| hsa-miR-20b-5p | NCOA3 | 1 | 1 | 1 | 3 |
| hsa-miR-20b-5p | HAUS8 | 1 | 1 | 1 | 3 |
| hsa-miR-129-5p | CBX4 | 1 | 1 | 1 | 3 |
| hsa-miR-20b-5p | LASP1 | 1 | 1 | 1 | 3 |
| hsa-miR-17-5p | ANKRD13C | 1 | 1 | 1 | 3 |
| hsa-miR-20b-5p | SGTB | 1 | 1 | 1 | 3 |
| hsa-miR-23b-3p | MET | 1 | 1 | 1 | 3 |
| hsa-miR-24-3p | VCPIP1 | 1 | 1 | 1 | 3 |
| hsa-miR-24-3p | PAK4 | 1 | 1 | 1 | 3 |
| hsa-miR-23b-3p | ZMYM2 | 1 | 1 | 1 | 3 |
| hsa-miR-20b-5p | LDLR | 1 | 1 | 1 | 3 |
| hsa-miR-17-5p | PXK | 1 | 1 | 1 | 3 |
| hsa-miR-17-5p | RUFY2 | 1 | 1 | 1 | 3 |
| hsa-miR-23b-3p | PDIA6 | 1 | 1 | 1 | 3 |
| hsa-miR-17-5p | FBXO31 | 1 | 1 | 1 | 3 |
| hsa-miR-24-3p | KLHDC3 | 1 | 1 | 1 | 3 |
| hsa-miR-429 | TPD52L1 | 1 | 1 | 1 | 3 |
| hsa-miR-17-5p | CNOT4 | 1 | 1 | 1 | 3 |
| hsa-miR-17-5p | TP53INP1 | 1 | 1 | 1 | 3 |
| hsa-miR-20b-5p | LIMA1 | 1 | 1 | 1 | 3 |
| hsa-miR-338-3p | ZWINT | 1 | 1 | 1 | 3 |
| hsa-miR-17-5p | ATG14 | 1 | 1 | 1 | 3 |
| hsa-miR-338-3p | MRPS23 | 1 | 1 | 1 | 3 |
| hsa-miR-338-3p | ARHGEF28 | 1 | 1 | 1 | 3 |
| hsa-miR-17-5p | ANKH | 1 | 1 | 1 | 3 |
| hsa-miR-17-5p | GIGYF1 | 1 | 1 | 1 | 3 |
| hsa-miR-17-5p | DNM1L | 1 | 1 | 1 | 3 |
| hsa-miR-20b-5p | SPRED1 | 1 | 1 | 1 | 3 |
| hsa-miR-17-5p | SERF1A | 1 | 1 | 1 | 3 |
| hsa-miR-425-5p | FOXJ3 | 1 | 1 | 1 | 3 |
| hsa-miR-24-3p | MT1E | 1 | 1 | 1 | 3 |
| hsa-miR-20b-5p | NPAT | 1 | 1 | 1 | 3 |
| hsa-miR-17-5p | PTPDC1 | 1 | 1 | 1 | 3 |
| hsa-miR-17-5p | FNBP1L | 1 | 1 | 1 | 3 |
| hsa-miR-17-5p | WDR37 | 1 | 1 | 1 | 3 |
| hsa-miR-338-3p | ORC4 | 1 | 1 | 1 | 3 |
| hsa-miR-20b-5p | PGM2L1 | 1 | 1 | 1 | 3 |
| hsa-miR-146b-5p | ZNRF3 | 1 | 1 | 1 | 3 |
| hsa-miR-20b-5p | PAFAH1B1 | 1 | 1 | 1 | 3 |
| hsa-miR-17-5p | CHD9 | 1 | 1 | 1 | 3 |
| hsa-miR-17-5p | PHTF2 | 1 | 1 | 1 | 3 |
| hsa-miR-429 | TBK1 | 1 | 1 | 1 | 3 |
| hsa-miR-17-5p | WEE1 | 1 | 1 | 1 | 3 |
| hsa-miR-17-5p | KMT2B | 1 | 1 | 1 | 3 |
| hsa-miR-216b-5p | CCDC65 | 1 | 1 | 1 | 3 |
| hsa-miR-20b-5p | MTF1 | 1 | 1 | 1 | 3 |
| hsa-miR-17-5p | RAP2C | 1 | 1 | 1 | 3 |
| hsa-miR-129-5p | KANK4 | 1 | 1 | 1 | 3 |
| hsa-miR-17-5p | GOLGA2 | 1 | 1 | 1 | 3 |
| hsa-miR-17-5p | UXS1 | 1 | 1 | 1 | 3 |
| hsa-miR-17-5p | RGMB | 1 | 1 | 1 | 3 |
| hsa-miR-20b-5p | MAP3K2 | 1 | 1 | 1 | 3 |
| hsa-miR-20b-5p | KLHL20 | 1 | 1 | 1 | 3 |
| hsa-miR-17-5p | PRR14L | 1 | 1 | 1 | 3 |
| hsa-miR-20b-5p | E2F2 | 1 | 1 | 1 | 3 |
| hsa-miR-429 | KIF13A | 1 | 1 | 1 | 3 |
| hsa-miR-129-5p | EI24 | 1 | 1 | 1 | 3 |
| hsa-miR-20b-5p | RBM20 | 1 | 1 | 1 | 3 |
| hsa-miR-20b-5p | DNAJC27 | 1 | 1 | 1 | 3 |
| hsa-miR-23b-3p | CA2 | 1 | 1 | 1 | 3 |
| hsa-miR-23b-3p | TAB3 | 1 | 1 | 1 | 3 |
| hsa-miR-17-5p | MCC | 1 | 1 | 1 | 3 |
| hsa-miR-20b-5p | RAP2C | 1 | 1 | 1 | 3 |
| hsa-miR-17-5p | TMEM167A | 1 | 1 | 1 | 3 |
| hsa-miR-17-5p | DUSP2 | 1 | 1 | 1 | 3 |
| hsa-miR-24-3p | CMTM4 | 1 | 1 | 1 | 3 |
| hsa-miR-20b-5p | ZBTB4 | 1 | 1 | 1 | 3 |
| hsa-miR-17-5p | EFCAB14 | 1 | 1 | 1 | 3 |
| hsa-miR-17-5p | ANKIB1 | 1 | 1 | 1 | 3 |
| hsa-miR-17-5p | DPYSL2 | 1 | 1 | 1 | 3 |
| hsa-miR-17-5p | ADARB1 | 1 | 1 | 1 | 3 |
| hsa-miR-17-5p | ARHGAP35 | 1 | 1 | 1 | 3 |
| hsa-miR-20b-5p | RAPGEF4 | 1 | 1 | 1 | 3 |
| hsa-miR-20b-5p | RPS6KA5 | 1 | 1 | 1 | 3 |
| hsa-miR-20b-5p | MAPRE3 | 1 | 1 | 1 | 3 |
| hsa-miR-20b-5p | BNIP2 | 1 | 1 | 1 | 3 |
| hsa-miR-20b-5p | NACC2 | 1 | 1 | 1 | 3 |
| hsa-miR-17-5p | SGMS1 | 1 | 1 | 1 | 3 |
| hsa-miR-20b-5p | REEP3 | 1 | 1 | 1 | 3 |
| hsa-miR-20b-5p | RLIM | 1 | 1 | 1 | 3 |
| hsa-miR-17-5p | EZH1 | 1 | 1 | 1 | 3 |
| hsa-miR-429 | TUBB2A | 1 | 1 | 1 | 3 |
| hsa-miR-17-5p | BRMS1L | 1 | 1 | 1 | 3 |
| hsa-miR-17-5p | E2F2 | 1 | 1 | 1 | 3 |
| hsa-miR-17-5p | PLXNA1 | 1 | 1 | 1 | 3 |
| hsa-miR-17-5p | LIMK1 | 1 | 1 | 1 | 3 |
| hsa-miR-17-5p | FBXL5 | 1 | 1 | 1 | 3 |
| hsa-miR-17-5p | MAPK1 | 1 | 1 | 1 | 3 |
| hsa-miR-20b-5p | ELK4 | 1 | 1 | 1 | 3 |
| hsa-miR-17-5p | ACSL4 | 1 | 1 | 1 | 3 |
| hsa-miR-20b-5p | DYNC1LI2 | 1 | 1 | 1 | 3 |
| hsa-miR-17-5p | NPAS3 | 1 | 1 | 1 | 3 |
| hsa-miR-17-5p | JAK1 | 1 | 1 | 1 | 3 |
| hsa-miR-17-5p | ATG16L1 | 1 | 1 | 1 | 3 |
| hsa-miR-17-5p | BMP8B | 1 | 1 | 1 | 3 |
| hsa-miR-17-5p | HSPA8 | 1 | 1 | 1 | 3 |
| hsa-miR-129-5p | ETV6 | 1 | 1 | 1 | 3 |
| hsa-miR-20b-5p | ZNF800 | 1 | 1 | 1 | 3 |
| hsa-miR-425-5p | PNMAL1 | 1 | 1 | 1 | 3 |
| hsa-miR-20b-5p | FAM126B | 1 | 1 | 1 | 3 |
| hsa-miR-216b-5p | ZNF566 | 1 | 1 | 1 | 3 |
| hsa-miR-24-3p | MXI1 | 1 | 1 | 1 | 3 |
| hsa-miR-20b-5p | FAM46C | 1 | 1 | 1 | 3 |
| hsa-miR-129-5p | CLOCK | 1 | 1 | 1 | 3 |
| hsa-miR-20b-5p | BICD2 | 1 | 1 | 1 | 3 |
| hsa-miR-216b-5p | KLF12 | 1 | 1 | 1 | 3 |
| hsa-miR-20b-5p | ANKRD50 | 1 | 1 | 1 | 3 |
| hsa-miR-338-3p | ACVR1 | 1 | 1 | 1 | 3 |
| hsa-miR-20b-5p | PKNOX1 | 1 | 1 | 1 | 3 |
| hsa-miR-24-3p | ABHD2 | 1 | 1 | 1 | 3 |
| hsa-miR-17-5p | FRMD6 | 1 | 1 | 1 | 3 |
| hsa-miR-17-5p | SLC16A9 | 1 | 1 | 1 | 3 |
| hsa-miR-17-5p | KLHL20 | 1 | 1 | 1 | 3 |
| hsa-miR-216b-5p | SMAD1 | 1 | 1 | 1 | 3 |
| hsa-miR-212-3p | WT1 | 1 | 1 | 1 | 3 |
| hsa-miR-20b-5p | ZNF532 | 1 | 1 | 1 | 3 |
| hsa-miR-425-5p | THRB | 1 | 1 | 1 | 3 |
| hsa-miR-20b-5p | RAB10 | 1 | 1 | 1 | 3 |
| hsa-miR-212-3p | BRWD1 | 1 | 1 | 1 | 3 |
| hsa-miR-129-5p | DNAJC15 | 1 | 1 | 1 | 3 |
| hsa-miR-17-5p | CLOCK | 1 | 1 | 1 | 3 |
| hsa-miR-24-3p | MAGI1 | 1 | 1 | 1 | 3 |
| hsa-miR-429 | ERMP1 | 1 | 1 | 1 | 3 |
| hsa-miR-24-3p | ZXDB | 1 | 1 | 1 | 3 |
| hsa-miR-20b-5p | FBXO31 | 1 | 1 | 1 | 3 |
| hsa-miR-17-5p | EPS15L1 | 1 | 1 | 1 | 3 |
| hsa-miR-17-5p | LDLR | 1 | 1 | 1 | 3 |
| hsa-miR-425-5p | NRAS | 1 | 1 | 1 | 3 |
| hsa-miR-20b-5p | SNTB2 | 1 | 1 | 1 | 3 |
| hsa-miR-20b-5p | U2SURP | 1 | 1 | 1 | 3 |
| hsa-miR-17-5p | RAB10 | 1 | 1 | 1 | 3 |
| hsa-miR-17-5p | ATL3 | 1 | 1 | 1 | 3 |
| hsa-miR-17-5p | NETO2 | 1 | 1 | 1 | 3 |
| hsa-miR-20b-5p | FOXJ2 | 1 | 1 | 1 | 3 |
| hsa-miR-24-3p | PTPRF | 1 | 1 | 1 | 3 |
| hsa-miR-24-3p | INMT | 1 | 1 | 1 | 3 |
| hsa-miR-20b-5p | MIDN | 1 | 1 | 1 | 3 |
| hsa-miR-24-3p | KCNK2 | 1 | 1 | 1 | 3 |
| hsa-miR-20b-5p | FRMD6 | 1 | 1 | 1 | 3 |
| hsa-miR-24-3p | UBE2K | 1 | 1 | 1 | 3 |
| hsa-miR-17-5p | ZNF417 | 1 | 1 | 1 | 3 |
| hsa-miR-24-3p | TOR2A | 1 | 1 | 1 | 3 |
| hsa-miR-17-5p | CRY2 | 1 | 1 | 1 | 3 |
| hsa-miR-212-3p | TJAP1 | 1 | 1 | 1 | 3 |
| hsa-miR-20b-5p | TOPORS | 1 | 1 | 1 | 3 |
| hsa-miR-20b-5p | ARID4B | 1 | 1 | 1 | 3 |
| hsa-miR-20b-5p | RABEP1 | 1 | 1 | 1 | 3 |
| hsa-miR-146b-5p | MMP16 | 1 | 1 | 1 | 3 |
| hsa-miR-20b-5p | RAB22A | 1 | 1 | 1 | 3 |
| hsa-miR-425-5p | SPPL2A | 1 | 1 | 1 | 3 |
| hsa-miR-425-5p | MAP3K5 | 1 | 1 | 1 | 3 |
| hsa-miR-24-3p | MIDN | 1 | 1 | 1 | 3 |
| hsa-miR-17-5p | ETV1 | 1 | 1 | 1 | 3 |
| hsa-miR-129-5p | HNRNPA3 | 1 | 1 | 1 | 3 |
| hsa-miR-17-5p | SOX4 | 1 | 1 | 1 | 3 |
| hsa-miR-20b-5p | CAPN15 | 1 | 1 | 1 | 3 |
| hsa-miR-20b-5p | HSPA8 | 1 | 1 | 1 | 3 |
| hsa-miR-216b-5p | FZD5 | 1 | 1 | 1 | 3 |
| hsa-miR-20b-5p | UNK | 1 | 1 | 1 | 3 |
| hsa-miR-20b-5p | PITPNA | 1 | 1 | 1 | 3 |
| hsa-miR-20b-5p | SEMA4B | 1 | 1 | 1 | 3 |
| hsa-miR-20b-5p | MCL1 | 1 | 1 | 1 | 3 |
| hsa-miR-20b-5p | TNFAIP1 | 1 | 1 | 1 | 3 |
| hsa-miR-17-5p | NABP1 | 1 | 1 | 1 | 3 |
| hsa-miR-17-5p | ANKRD12 | 1 | 1 | 1 | 3 |
| hsa-miR-20b-5p | KATNAL1 | 1 | 1 | 1 | 3 |
| hsa-miR-129-5p | LRRC2 | 1 | 1 | 1 | 3 |
| hsa-miR-17-5p | FAM126B | 1 | 1 | 1 | 3 |
| hsa-miR-20b-5p | PPP6C | 1 | 1 | 1 | 3 |
| hsa-miR-20b-5p | RGMB | 1 | 1 | 1 | 3 |
| hsa-miR-20b-5p | RRM2 | 1 | 1 | 1 | 3 |
| hsa-miR-338-3p | NRP1 | 1 | 1 | 1 | 3 |
| hsa-miR-24-3p | INSIG1 | 1 | 1 | 1 | 3 |
| hsa-miR-17-5p | LPGAT1 | 1 | 1 | 1 | 3 |
| hsa-miR-129-5p | CBX6 | 1 | 1 | 1 | 3 |
| hsa-miR-17-5p | SEMA7A | 1 | 1 | 1 | 3 |
| hsa-miR-17-5p | CASP2 | 1 | 1 | 1 | 3 |
| hsa-miR-17-5p | USP32 | 1 | 1 | 1 | 3 |
| hsa-miR-429 | CELF1 | 1 | 1 | 1 | 3 |
| hsa-miR-17-5p | TRIM37 | 1 | 1 | 1 | 3 |
| hsa-miR-20b-5p | ZBTB7A | 1 | 1 | 1 | 3 |
| hsa-miR-17-5p | SPRED1 | 1 | 1 | 1 | 3 |
| hsa-miR-17-5p | FYCO1 | 1 | 1 | 1 | 3 |
| hsa-miR-20b-5p | EZH1 | 1 | 1 | 1 | 3 |
| hsa-miR-17-5p | KIAA1191 | 1 | 1 | 1 | 3 |
| hsa-miR-20b-5p | STX6 | 1 | 1 | 1 | 3 |
| hsa-miR-24-3p | MARCKSL1 | 1 | 1 | 1 | 3 |
| hsa-miR-17-5p | RBM12B | 1 | 1 | 1 | 3 |
| hsa-miR-23b-3p | BRWD1 | 1 | 1 | 1 | 3 |
| hsa-miR-20b-5p | E2F1 | 1 | 1 | 1 | 3 |
| hsa-miR-17-5p | HAS2 | 1 | 1 | 1 | 3 |
| hsa-miR-17-5p | EPHA4 | 1 | 1 | 1 | 3 |
| hsa-miR-20b-5p | SSX2IP | 1 | 1 | 1 | 3 |
| hsa-miR-17-5p | SMOC1 | 1 | 1 | 1 | 3 |
| hsa-miR-23b-3p | TNFAIP3 | 1 | 1 | 1 | 3 |
| hsa-miR-20b-5p | MORF4L1 | 1 | 1 | 1 | 3 |
| hsa-miR-20b-5p | C14orf28 | 1 | 1 | 1 | 3 |
| hsa-miR-20b-5p | SCAMP2 | 1 | 1 | 1 | 3 |
| hsa-miR-17-5p | ZBTB9 | 1 | 1 | 1 | 3 |
| hsa-miR-20b-5p | ORMDL3 | 1 | 1 | 1 | 3 |
| hsa-miR-425-5p | MAP2K6 | 1 | 1 | 1 | 3 |
| hsa-miR-129-5p | ABCC5 | 1 | 1 | 1 | 3 |
| hsa-miR-20b-5p | KPNA2 | 1 | 1 | 1 | 3 |
| hsa-miR-24-3p | VGLL3 | 1 | 1 | 1 | 3 |
| hsa-miR-17-5p | AKTIP | 1 | 1 | 1 | 3 |
| hsa-miR-20b-5p | CLIP4 | 1 | 1 | 1 | 3 |
| hsa-miR-23b-3p | SEMA6D | 1 | 1 | 1 | 3 |
| hsa-miR-129-5p | ZNF25 | 1 | 1 | 1 | 3 |
| hsa-miR-20b-5p | FJX1 | 1 | 1 | 1 | 3 |
| hsa-miR-425-5p | DICER1 | 1 | 1 | 1 | 3 |
| hsa-miR-20b-5p | VPS26A | 1 | 1 | 1 | 3 |
| hsa-miR-17-5p | PTGFRN | 1 | 1 | 1 | 3 |
| hsa-miR-17-5p | FAM117B | 1 | 1 | 1 | 3 |
| hsa-miR-17-5p | NIN | 1 | 1 | 1 | 3 |
| hsa-miR-17-5p | TMEM245 | 1 | 1 | 1 | 3 |
| hsa-miR-17-5p | DDHD1 | 1 | 1 | 1 | 3 |
| hsa-miR-429 | DENND5B | 1 | 1 | 1 | 3 |
| hsa-miR-20b-5p | PHTF2 | 1 | 1 | 1 | 3 |
| hsa-miR-17-5p | TET3 | 1 | 1 | 1 | 3 |
| hsa-miR-17-5p | CADM2 | 1 | 1 | 1 | 3 |
| hsa-miR-17-5p | SPOPL | 1 | 1 | 1 | 3 |
| hsa-miR-17-5p | GBF1 | 1 | 1 | 1 | 3 |
| hsa-miR-17-5p | NAGK | 1 | 1 | 1 | 3 |
| hsa-miR-17-5p | CREB1 | 1 | 1 | 1 | 3 |
| hsa-miR-129-5p | SPRY4 | 1 | 1 | 1 | 3 |
| hsa-miR-425-5p | CREBZF | 1 | 1 | 1 | 3 |
| hsa-miR-23b-3p | MARCKS | 1 | 1 | 1 | 3 |
| hsa-miR-24-3p | SESN1 | 1 | 1 | 1 | 3 |
| hsa-miR-129-5p | RSBN1 | 1 | 1 | 1 | 3 |
| hsa-miR-17-5p | ZNF280B | 1 | 1 | 1 | 3 |
| hsa-miR-129-5p | HBS1L | 1 | 1 | 1 | 3 |
| hsa-miR-17-5p | SLC22A23 | 1 | 1 | 1 | 3 |
| hsa-miR-17-5p | ZFYVE26 | 1 | 1 | 1 | 3 |
| hsa-miR-20b-5p | ZNF280B | 1 | 1 | 1 | 3 |
| hsa-miR-20b-5p | VPS13C | 1 | 1 | 1 | 3 |
| hsa-miR-20b-5p | LYSMD3 | 1 | 1 | 1 | 3 |
| hsa-miR-23b-3p | TNRC6A | 1 | 1 | 1 | 3 |
| hsa-miR-24-3p | C1orf106 | 1 | 1 | 1 | 3 |
| hsa-miR-17-5p | UNK | 1 | 1 | 1 | 3 |
| hsa-miR-20b-5p | CHD9 | 1 | 1 | 1 | 3 |
| hsa-miR-338-3p | NOVA1 | 1 | 1 | 1 | 3 |
| hsa-miR-24-3p | TMEM209 | 1 | 1 | 1 | 3 |
| hsa-miR-17-5p | EEA1 | 1 | 1 | 1 | 3 |
| hsa-miR-20b-5p | CRIM1 | 1 | 1 | 1 | 3 |
| hsa-miR-20b-5p | FRS2 | 1 | 1 | 1 | 3 |
| hsa-miR-17-5p | FJX1 | 1 | 1 | 1 | 3 |
| hsa-miR-24-3p | CNNM3 | 1 | 1 | 1 | 3 |
| hsa-miR-17-5p | HIP1 | 1 | 1 | 1 | 3 |
| hsa-miR-17-5p | ARHGAP12 | 1 | 1 | 1 | 3 |
| hsa-miR-20b-5p | ZBTB18 | 1 | 1 | 1 | 3 |
| hsa-miR-216b-5p | SOCS6 | 1 | 1 | 1 | 3 |
| hsa-miR-17-5p | REST | 1 | 1 | 1 | 3 |
| hsa-miR-17-5p | TNFRSF21 | 1 | 1 | 1 | 3 |
| hsa-miR-20b-5p | ZNF202 | 1 | 1 | 1 | 3 |
| hsa-miR-20b-5p | TXLNA | 1 | 1 | 1 | 3 |
| hsa-miR-23b-3p | CELF1 | 1 | 1 | 1 | 3 |
| hsa-miR-129-5p | BMPR2 | 1 | 1 | 1 | 3 |
| hsa-miR-17-5p | ZC3H12C | 1 | 1 | 1 | 3 |
| hsa-miR-20b-5p | SUCO | 1 | 1 | 1 | 3 |
| hsa-miR-17-5p | HBP1 | 1 | 1 | 1 | 3 |
| hsa-miR-129-5p | DAB2 | 1 | 1 | 1 | 3 |
| hsa-miR-17-5p | RAB11FIP1 | 1 | 1 | 1 | 3 |
| hsa-miR-23b-3p | RBPMS2 | 1 | 1 | 1 | 3 |
| hsa-miR-24-3p | SCML2 | 1 | 1 | 1 | 3 |
| hsa-miR-20b-5p | SQSTM1 | 1 | 1 | 1 | 3 |
| hsa-miR-429 | SEC23A | 1 | 1 | 1 | 3 |
| hsa-miR-20b-5p | KIAA1147 | 1 | 1 | 1 | 3 |
| hsa-miR-17-5p | MTF1 | 1 | 1 | 1 | 3 |
| hsa-miR-17-5p | HIF1A | 1 | 1 | 1 | 3 |
| hsa-miR-17-5p | PITPNA | 1 | 1 | 1 | 3 |
| hsa-miR-17-5p | ELAVL2 | 1 | 1 | 1 | 3 |
| hsa-miR-17-5p | CLIP4 | 1 | 1 | 1 | 3 |
| hsa-miR-20b-5p | RRAGD | 1 | 1 | 1 | 3 |
| hsa-miR-20b-5p | CADM2 | 1 | 1 | 1 | 3 |
| hsa-miR-23b-3p | PRR14L | 1 | 1 | 1 | 3 |
| hsa-miR-17-5p | PHF6 | 1 | 1 | 1 | 3 |
| hsa-miR-23b-3p | CNN2 | 1 | 1 | 1 | 3 |
| hsa-miR-23b-3p | SESN2 | 1 | 1 | 1 | 3 |
| hsa-miR-24-3p | ADPGK | 1 | 1 | 1 | 3 |
| hsa-miR-20b-5p | F3 | 1 | 1 | 1 | 3 |
| hsa-miR-17-5p | ABCA1 | 1 | 1 | 1 | 3 |
| hsa-miR-20b-5p | POLR3G | 1 | 1 | 1 | 3 |
| hsa-miR-17-5p | LCOR | 1 | 1 | 1 | 3 |
| hsa-miR-20b-5p | ANKRD52 | 1 | 1 | 1 | 3 |
| hsa-miR-17-5p | SGTB | 1 | 1 | 1 | 3 |
| hsa-miR-216b-5p | ARL6IP1 | 1 | 1 | 1 | 3 |
| hsa-miR-17-5p | F3 | 1 | 1 | 1 | 3 |
| hsa-miR-129-5p | PAPD5 | 1 | 1 | 1 | 3 |
| hsa-miR-20b-5p | TET3 | 1 | 1 | 1 | 3 |
| hsa-miR-17-5p | E2F5 | 1 | 1 | 1 | 3 |
| hsa-miR-17-5p | USP28 | 1 | 1 | 1 | 3 |
| hsa-miR-20b-5p | TGFBR2 | 1 | 1 | 1 | 3 |
| hsa-miR-17-5p | FOXQ1 | 1 | 1 | 1 | 3 |
| hsa-miR-17-5p | DCBLD2 | 1 | 1 | 1 | 3 |
| hsa-miR-17-5p | STX6 | 1 | 1 | 1 | 3 |
| hsa-miR-429 | CCNT2 | 1 | 1 | 1 | 3 |
| hsa-miR-20b-5p | RBBP7 | 1 | 1 | 1 | 3 |
| hsa-miR-216b-5p | DNAJB9 | 1 | 1 | 1 | 3 |
| hsa-miR-17-5p | NAPEPLD | 1 | 1 | 1 | 3 |
| hsa-miR-425-5p | SYNCRIP | 1 | 1 | 1 | 3 |
| hsa-miR-20b-5p | RUNDC1 | 1 | 1 | 1 | 3 |
| hsa-miR-17-5p | PFKP | 1 | 1 | 1 | 3 |
| hsa-miR-20b-5p | TNKS2 | 1 | 1 | 1 | 3 |
| hsa-miR-20b-5p | DDX5 | 1 | 1 | 1 | 3 |
| hsa-miR-20b-5p | OCRL | 1 | 1 | 1 | 3 |
| hsa-miR-23b-3p | MMGT1 | 1 | 1 | 1 | 3 |
| hsa-miR-17-5p | SEPT2 | 1 | 1 | 1 | 3 |
| hsa-miR-17-5p | RPS6KA5 | 1 | 1 | 1 | 3 |
| hsa-miR-20b-5p | TMEM167A | 1 | 1 | 1 | 3 |
| hsa-miR-17-5p | PLEKHO2 | 1 | 1 | 1 | 3 |
| hsa-miR-17-5p | GID4 | 1 | 1 | 1 | 3 |
| hsa-miR-20b-5p | AGO1 | 1 | 1 | 1 | 3 |
| hsa-miR-17-5p | KPNA2 | 1 | 1 | 1 | 3 |
| hsa-miR-20b-5p | SESN3 | 1 | 1 | 1 | 3 |
| hsa-miR-17-5p | CCND1 | 1 | 1 | 1 | 3 |
| hsa-miR-338-3p | ZDHHC18 | 1 | 1 | 1 | 3 |
| hsa-miR-17-5p | BICD2 | 1 | 1 | 1 | 3 |
| hsa-miR-24-3p | CCDC58 | 1 | 1 | 1 | 3 |
| hsa-miR-17-5p | ELK4 | 1 | 1 | 1 | 3 |
| hsa-miR-17-5p | STK17B | 1 | 1 | 1 | 3 |
| hsa-miR-17-5p | PPP3R1 | 1 | 1 | 1 | 3 |
| hsa-miR-20b-5p | SOX4 | 1 | 1 | 1 | 3 |
| hsa-miR-129-5p | CNTLN | 1 | 1 | 1 | 3 |
| hsa-miR-24-3p | H2AFX | 1 | 1 | 1 | 3 |

| 621 mRNAs after deduplication | | | | | |
| --- | --- | --- | --- | --- | --- |
| MAP3K3 | ANKRD12 | PSD3 | TGFBR2 | SCML1 | TAB3 |
| HMGB2 | ABCA1 | C7orf43 | SQSTM1 | TNFRSF21 | TMEM167A |
| PFKP | TBC1D15 | AFF4 | CCDC71L | CALM1 | CMTM4 |
| HMBOX1 | CEP97 | MATR3 | TADA2B | EEA1 | ADARB1 |
| PIP4K2A | EIF4H | BCOR | CREB1 | RUNDC1 | RPS6KA5 |
| GNB5 | IRAK1 | POLR3D | CASP2 | STON2 | SGMS1 |
| TMEM123 | LASP1 | FURIN | ANKIB1 | MAP3K2 | EZH1 |
| CAB39 | NFAT5 | C17orf49 | CDKN1A | HMGB1 | TUBB2A |
| RAPGEF4 | USP3 | SLK | KMT2B | CTSA | ELK4 |
| UNC5D | STAT5B | RRAS2 | DDN | FAM46C | NPAS3 |
| SSR1 | ADD3 | PHF6 | SOX4 | FNBP1L | BMP8B |
| MCFD2 | PPP6R3 | PPP2CB | FMR1 | C11orf57 | HSPA8 |
| PXK | IFNAR1 | MBD6 | RAB11FIP1 | RAB31 | ETV6 |
| FCHO2 | KIAA0922 | CRY2 | CNOT7 | PNRC2 | PNMAL1 |
| PTEN | DIS3 | PDPK1 | DND1 | SLC16A9 | FAM126B |
| MTMR3 | KIAA0513 | TSC22D2 | FAM117B | USP28 | ZNF566 |
| FAM129A | TNKS2 | PLS1 | ZNFX1 | FUT4 | MXI1 |
| CERCAM | SCAMP5 | OSTM1 | KIF23 | RABEP1 | BICD2 |
| LBR | U2SURP | HAS2 | SESN3 | ARHGAP35 | KLF12 |
| TMEM138 | PREX2 | STK17B | HKR1 | NIN | ACVR1 |
| MASTL | RPRD2 | PIM2 | FBLIM1 | NACC2 | FRMD6 |
| SORBS2 | PER2 | PLXNA1 | AMD1 | UQCRFS1 | SMAD1 |
| BAMBI | ZNF800 | FRS2 | ZXDA | TMEM245 | WT1 |
| RAB5B | TPM3 | PTPN9 | NCOA5 | VPS13C | THRB |
| ABHD2 | NDST1 | FOXJ3 | AKAP10 | FBN2 | RAB10 |
| C15orf39 | PTPN4 | MAP3K9 | RNF145 | BEX4 | BRWD1 |
| AKIP1 | SBNO1 | ORMDL3 | AGO1 | DDX5 | DNAJC15 |
| BCL7A | ADD1 | PANK3 | EGLN3 | TRIM37 | MAGI1 |
| NRIP3 | ANKRD50 | POLQ | ZBTB9 | ARHGAP12 | ERMP1 |
| GNAQ | IFNG | ZBTB18 | RNF2 | YRDC | ZXDB |
| AMOTL2 | DNAJB6 | PPP1CB | RNF168 | DYRK2 | EPS15L1 |
| SAMD12 | RORA | MAPK1 | HNRNPD | KAT2B | NRAS |
| LAPTM4A | SIK1 | SNTB2 | DNAJC27 | NABP1 | PTPRF |
| ATAD2 | MCM4 | BMPR2 | PRR14L | RPF2 | INMT |
| MLXIP | FBXO28 | TMEM64 | FEM1C | PDZD11 | KCNK2 |
| GINS4 | AKAP11 | TERF2 | DUSP10 | NETO2 | UBE2K |
| TNPO1 | KLHL5 | CAPN15 | UXS1 | CEP170 | ZNF417 |
| ASF1A | CLOCK | FBXO21 | NHLRC3 | SOD2 | TOR2A |
| CEP57 | STAT3 | TMEM242 | PMAIP1 | C9orf40 | TJAP1 |
| ZMAT3 | CENPQ | RYBP | TBC1D2 | TRAF6 | MMP16 |
| E2F1 | DUSP2 | FYCO1 | FSCN1 | COL4A4 | SPPL2A |
| ZFYVE9 | PIP4K2C | RNF11 | ZC3H12C | BAP1 | MAP3K5 |
| NPAT | LIMA1 | RCCD1 | ARID4B | NIPA1 | ETV1 |
| UBE2Q2 | FBXL5 | CD34 | LCOR | DDHD1 | HNRNPA3 |
| SLAIN2 | ZNF217 | ZNF202 | TWF1 | CCND1 | FZD5 |
| PPP1R3B | GOLGA1 | TMEM173 | GAB1 | CCDC169 | UNK |
| MIDN | ZBTB4 | ACVR1B | TM9SF3 | EFCAB14 | PITPNA |
| AVL9 | NAGK | NKIRAS1 | SGTB | FXR1 | LRRC2 |
| PPP1R15B | IRAK4 | GBA2 | CAPRIN2 | SHCBP1 | NRP1 |
| CHUK | TANC1 | ATXN7L3B | MAP3K12 | DPYSL2 | INSIG1 |
| FNIP2 | RBM47 | MLEC | RAP2C | PEA15 | CBX6 |
| LYSMD3 | ZDHHC9 | UBXN2A | PRDM15 | DNAJB12 | CELF1 |
| ULK1 | LIMK1 | KLF3 | SSX2IP | RRM2 | KIAA1191 |
| C8orf58 | SEMA4B | PARD6B | MAPRE3 | SIKE1 | STX6 |
| HMGB3 | SMAD5 | RLIM | POM121C | GJA1 | MARCKSL1 |
| PHF20L1 | KIAA1147 | SRSF2 | VPS26A | PDXK | RBM12B |
| TAOK1 | FAM210A | LIMS1 | PTK2B | SPIN4 | TNFAIP3 |
| CMPK1 | EPHA4 | RUFY2 | SCAMP2 | ATP5G3 | MAP2K6 |
| GALNT1 | CRK | FOXJ2 | ZNF107 | BHLHB9 | ABCC5 |
| TRIP10 | LPGAT1 | LIMD1 | CYBRD1 | MAPK14 | KPNA2 |
| RHOC | NUFIP2 | EOGT | EBF1 | KATNAL1 | VGLL3 |
| SEC24A | ITCH | BBX | MAPK7 | SEMA7A | CLIP4 |
| CHIC1 | TXLNA | NCOA3 | REST | TOP1 | SEMA6D |
| ITGB8 | CIT | SMOC1 | TNFAIP1 | C14orf28 | ZNF25 |
| GIGYF1 | KLHL28 | ATG16L1 | JAK1 | SEPT2 | FJX1 |
| MSMO1 | ARAP2 | FAM57A | FAM102A | PAFAH1B1 | DICER1 |
| RBMXL1 | SLC22A23 | SOCS6 | CDK6 | PPP3R1 | TET3 |
| CNOT6L | FBXO48 | GPAM | ZBTB7A | TFAM | CADM2 |
| NRBP1 | SUCO | UBE3C | SLC16A1 | JUN | SPRY4 |
| GPATCH8 | KAT6B | RAB22A | C1S | CBX4 | CREBZF |
| DENND5B | ACSL4 | BTBD7 | NPAS2 | MET | MARCKS |
| ANKRD52 | BTG3 | ANKRD33B | ATL3 | VCPIP1 | SESN1 |
| OCRL | GABBR2 | CHAF1A | BMPER | PAK4 | RSBN1 |
| KLF10 | RBL1 | YOD1 | FAXC | ZMYM2 | ZNF280B |
| ANKRD17 | APC | REEP3 | AKTIP | LDLR | HBS1L |
| QSER1 | PPP6C | TMBIM6 | FGFR3 | PDIA6 | TNRC6A |
| TRPM6 | RNF165 | NUP35 | GBF1 | FBXO31 | C1orf106 |
| RB1 | ATG14 | EIF4G2 | BNIP2 | KLHDC3 | NOVA1 |
| ARHGAP1 | CAMTA1 | NLGN4X | ANKRD13C | TPD52L1 | TMEM209 |
| PFAS | MFSD8 | ARID2 | NR2C2 | CNOT4 | CRIM1 |
| DNAJB9 | HIC2 | DYNC1LI2 | PKNOX1 | ZWINT | CNNM3 |
| RRAGD | TMEM127 | ZFYVE26 | RBL2 | MRPS23 | HIP1 |
| TSG101 | RUNX3 | SLBP | ZNF532 | ARHGEF28 | DAB2 |
| ZCCHC2 | PLEKHM1 | SEMA6A | SALL3 | DNM1L | RBPMS2 |
| CCSER2 | ETNK1 | ANKFY1 | RBBP7 | SPRED1 | SCML2 |
| ZEB1 | AP5M1 | PKD2 | MORF4L1 | SERF1A | SEC23A |
| STK11 | RPA2 | HBP1 | USP32 | MT1E | CNN2 |
| OXR1 | WASF3 | POLR3G | PRDM1 | ORC4 | SESN2 |
| CFL2 | MKNK2 | MAP3K8 | SDHD | ZNRF3 | ADPGK |
| MMP2 | PTP4A1 | MEN1 | SACS | CHD9 | F3 |
| PRRG4 | POGZ | ANKH | PTPDC1 | PHTF2 | ARL6IP1 |
| PTGFRN | BTN3A1 | MINK1 | DVL3 | TBK1 | PAPD5 |
| TP53INP1 | MCC | ERAP1 | TOPORS | WEE1 | DCBLD2 |
| BTLA | BCL2L11 | WDR37 | FNIP1 | CCDC65 | CCNT2 |
| HAUS8 | USP6NL | PLEKHO2 | NWD1 | MTF1 | NAPEPLD |
| PDLIM5 | NCOA2 | RASSF8 | SSH2 | KANK4 | SYNCRIP |
| GNS | MCL1 | SERF1B | KMT2A | GOLGA2 | MMGT1 |
| TXNIP | CCL1 | EMP2 | EXPH5 | RGMB | ZDHHC18 |
| EIF5A2 | REEP1 | HIF1A | CPOX | KLHL20 | CCDC58 |
| CRIPT | MAPK9 | PLIN3 | PGM2L1 | E2F2 | CNTLN |
| ZFPM2 | ELAVL2 | SHOC2 | ARIH1 | KIF13A | H2AFX |
| CAMSAP2 | WAC | FOXQ1 | STRADB | EI24 |  |
| BRMS1L | GHITM | SKIL | TMED7 | RBM20 |  |
| SPOPL | ENPP5 | GID4 | E2F5 | CA2 |  |
