# Supplementary material for: Zuogui Pill Ameliorates Glucocorticoid-Induced Osteoporosis through ZNF702P-Based ceRNA Network: Bioinformatics Analysis and Experimental Validation
Source: Evid Based Complement Alternat Med. 2022 Aug 29;2022:8020182. doi: 10.1155/2022/8020182 (PMC11401717; doi:10.1155/2022/8020182)
Supplement: Supplementary Materials — Supplementary Table 1. LncRNA-miRNA-mRNA interactions. Supplementary Table 2. The drugs, active compounds, and targets of ZGP. Supplementary Table 3. Detailed network topology information of intersection targets. [file 8020182.f1.zip › 8020182.f1/Supplementary Table 2 The drugs, active compounds and targets of ZGP.docx]

Supplementary Table 2. The drugs, active compounds and targets of ZGP

| Drug | Compound | Target |
| --- | --- | --- |
| Niu Xi | Rubrosterone | NR3C2 |
| Niu Xi | Betavulgarin | NOS2 |
| Niu Xi | Betavulgarin | PTGS1 |
| Niu Xi | Betavulgarin | F2 |
| Niu Xi | Betavulgarin | KCNH2 |
| Niu Xi | Betavulgarin | ESR1 |
| Niu Xi | Betavulgarin | AR |
| Niu Xi | Betavulgarin | SCN5A |
| Niu Xi | Betavulgarin | PPARG |
| Niu Xi | Betavulgarin | PTGS2 |
| Niu Xi | Betavulgarin | CA2 |
| Niu Xi | Betavulgarin | F7 |
| Niu Xi | Betavulgarin | RXRA |
| Niu Xi | Betavulgarin | ESR2 |
| Niu Xi | Betavulgarin | DPP4 |
| Niu Xi | Betavulgarin | MAPK14 |
| Niu Xi | Betavulgarin | GSK3B |
| Niu Xi | Betavulgarin | HSP90AB1 |
| Niu Xi | Betavulgarin | CDK2 |
| Niu Xi | Betavulgarin | PIK3CG |
| Niu Xi | Betavulgarin | CHEK1 |
| Niu Xi | Betavulgarin | IGHG1 |
| Niu Xi | Betavulgarin | PRSS1 |
| Niu Xi | Betavulgarin | KCNMA1 |
| Niu Xi | Betavulgarin | CALM1 |
| Niu Xi | beta-sitosterol | PGR |
| Niu Xi | beta-sitosterol | NCOA2 |
| Niu Xi | beta-sitosterol | PTGS1 |
| Niu Xi | beta-sitosterol | PTGS2 |
| Niu Xi | beta-sitosterol | HSP90AB1 |
| Niu Xi | beta-sitosterol | PIK3CG |
| Niu Xi | beta-sitosterol | KCNH2 |
| Niu Xi | beta-sitosterol | PRKACA |
| Niu Xi | beta-sitosterol | DRD5 |
| Niu Xi | beta-sitosterol | CHRM3 |
| Niu Xi | beta-sitosterol | CHRM1 |
| Niu Xi | beta-sitosterol | SCN5A |
| Niu Xi | beta-sitosterol | GABRA2 |
| Niu Xi | beta-sitosterol | CHRM4 |
| Niu Xi | beta-sitosterol | PDE3A |
| Niu Xi | beta-sitosterol | HTR2A |
| Niu Xi | beta-sitosterol | GABRA3 |
| Niu Xi | beta-sitosterol | ADRA1A |
| Niu Xi | beta-sitosterol | CHRM2 |
| Niu Xi | beta-sitosterol | ADRA1B |
| Niu Xi | beta-sitosterol | ADRB2 |
| Niu Xi | beta-sitosterol | CHRNA2 |
| Niu Xi | beta-sitosterol | SLC6A4 |
| Niu Xi | beta-sitosterol | OPRM1 |
| Niu Xi | beta-sitosterol | GABRA1 |
| Niu Xi | beta-sitosterol | CHRNA7 |
| Niu Xi | beta-sitosterol | BCL2 |
| Niu Xi | beta-sitosterol | BAX |
| Niu Xi | beta-sitosterol | CASP9 |
| Niu Xi | beta-sitosterol | JUN |
| Niu Xi | beta-sitosterol | CASP3 |
| Niu Xi | beta-sitosterol | CASP8 |
| Niu Xi | beta-sitosterol | PRKCA |
| Niu Xi | beta-sitosterol | TGFB1 |
| Niu Xi | beta-sitosterol | PON1 |
| Niu Xi | beta-sitosterol | MAP2 |
| Niu Xi | quercetin | PTGS1 |
| Niu Xi | quercetin | AR |
| Niu Xi | quercetin | PPARG |
| Niu Xi | quercetin | PTGS2 |
| Niu Xi | quercetin | HSP90AB1 |
| Niu Xi | quercetin | PIK3CG |
| Niu Xi | quercetin | NCOA2 |
| Niu Xi | quercetin | DPP4 |
| Niu Xi | quercetin | AKR1B10 |
| Niu Xi | quercetin | PRSS1 |
| Niu Xi | quercetin | TOP2A |
| Niu Xi | quercetin | F2 |
| Niu Xi | quercetin | KCNH2 |
| Niu Xi | quercetin | SCN5A |
| Niu Xi | quercetin | F10 |
| Niu Xi | quercetin | ADRB2 |
| Niu Xi | quercetin | MMP3 |
| Niu Xi | quercetin | PRKACA |
| Niu Xi | quercetin | F7 |
| Niu Xi | quercetin | NOS3 |
| Niu Xi | quercetin | RXRA |
| Niu Xi | quercetin | ACHE |
| Niu Xi | quercetin | GABRA1 |
| Niu Xi | quercetin | MAOB |
| Niu Xi | quercetin | RELA |
| Niu Xi | quercetin | EGFR |
| Niu Xi | quercetin | AKT1 |
| Niu Xi | quercetin | VEGFA |
| Niu Xi | quercetin | CCND1 |
| Niu Xi | quercetin | BCL2 |
| Niu Xi | quercetin | BCL2L1 |
| Niu Xi | quercetin | FOS |
| Niu Xi | quercetin | CDKN1A |
| Niu Xi | quercetin | EIF6 |
| Niu Xi | quercetin | BAX |
| Niu Xi | quercetin | CASP9 |
| Niu Xi | quercetin | PLAU |
| Niu Xi | quercetin | MMP2 |
| Niu Xi | quercetin | MMP9 |
| Niu Xi | quercetin | MAPK1 |
| Niu Xi | quercetin | IL10 |
| Niu Xi | quercetin | EGF |
| Niu Xi | quercetin | RB1 |
| Niu Xi | quercetin | TNF |
| Niu Xi | quercetin | JUN |
| Niu Xi | quercetin | IL6 |
| Niu Xi | quercetin | TP53 |
| Niu Xi | quercetin | AHSA1 |
| Niu Xi | quercetin | CASP3 |
| Niu Xi | quercetin | ELK1 |
| Niu Xi | quercetin | NFKBIA |
| Niu Xi | quercetin | POR |
| Niu Xi | quercetin | ODC1 |
| Niu Xi | quercetin | XDH |
| Niu Xi | quercetin | CASP8 |
| Niu Xi | quercetin | TOP1 |
| Niu Xi | quercetin | RAF1 |
| Niu Xi | quercetin | SOD1 |
| Niu Xi | quercetin | PRKCA |
| Niu Xi | quercetin | MMP1 |
| Niu Xi | quercetin | HIF1A |
| Niu Xi | quercetin | STAT1 |
| Niu Xi | quercetin | RUNX1T1 |
| Niu Xi | quercetin | CDK1 |
| Niu Xi | quercetin | HSPA5 |
| Niu Xi | quercetin | ERBB2 |
| Niu Xi | quercetin | ACACA |
| Niu Xi | quercetin | HMOX1 |
| Niu Xi | quercetin | CYP3A4 |
| Niu Xi | quercetin | CYP1A2 |
| Niu Xi | quercetin | CAV1 |
| Niu Xi | quercetin | MYC |
| Niu Xi | quercetin | F3 |
| Niu Xi | quercetin | GJA1 |
| Niu Xi | quercetin | CYP1A1 |
| Niu Xi | quercetin | ICAM1 |
| Niu Xi | quercetin | IL1B |
| Niu Xi | quercetin | CCL2 |
| Niu Xi | quercetin | SELE |
| Niu Xi | quercetin | VCAM1 |
| Niu Xi | quercetin | PTGER3 |
| Niu Xi | quercetin | CXCL8 |
| Niu Xi | quercetin | PRKCB |
| Niu Xi | quercetin | BIRC5 |
| Niu Xi | quercetin | DUOX2 |
| Niu Xi | quercetin | HSPB1 |
| Niu Xi | quercetin | TGFB1 |
| Niu Xi | quercetin | SULT1E1 |
| Niu Xi | quercetin | MGAM |
| Niu Xi | quercetin | IL2 |
| Niu Xi | quercetin | NR1I2 |
| Niu Xi | quercetin | CYP1B1 |
| Niu Xi | quercetin | CCNB1 |
| Niu Xi | quercetin | PLAT |
| Niu Xi | quercetin | THBD |
| Niu Xi | quercetin | SERPINE1 |
| Niu Xi | quercetin | COL19A1 |
| Niu Xi | quercetin | IFNG |
| Niu Xi | quercetin | ALOX5 |
| Niu Xi | quercetin | PTEN |
| Niu Xi | quercetin | IL1A |
| Niu Xi | quercetin | MPO |
| Niu Xi | quercetin | NCF1 |
| Niu Xi | quercetin | ABCG1 |
| Niu Xi | quercetin | HAS2 |
| Niu Xi | quercetin | GSTP1 |
| Niu Xi | quercetin | NFE2L2 |
| Niu Xi | quercetin | NQO1 |
| Niu Xi | quercetin | TNKS |
| Niu Xi | quercetin | AHR |
| Niu Xi | quercetin | PSMD3 |
| Niu Xi | quercetin | SLC2A4 |
| Niu Xi | quercetin | COL3A1 |
| Niu Xi | quercetin | CXCL11 |
| Niu Xi | quercetin | CXCL2 |
| Niu Xi | quercetin | DCAF5 |
| Niu Xi | quercetin | NR1I3 |
| Niu Xi | quercetin | CHEK2 |
| Niu Xi | quercetin | INSR |
| Niu Xi | quercetin | CLDN4 |
| Niu Xi | quercetin | PPARA |
| Niu Xi | quercetin | PPARD |
| Niu Xi | quercetin | HSF1 |
| Niu Xi | quercetin | CRP |
| Niu Xi | quercetin | CXCL10 |
| Niu Xi | quercetin | CHUK |
| Niu Xi | quercetin | SPP1 |
| Niu Xi | quercetin | RUNX2 |
| Niu Xi | quercetin | RASSF1 |
| Niu Xi | quercetin | E2F1 |
| Niu Xi | quercetin | E2F2 |
| Niu Xi | quercetin | ACP3 |
| Niu Xi | quercetin | CTSD |
| Niu Xi | quercetin | IGFBP3 |
| Niu Xi | quercetin | IGF2 |
| Niu Xi | quercetin | CD40LG |
| Niu Xi | quercetin | IRF1 |
| Niu Xi | quercetin | ERBB3 |
| Niu Xi | quercetin | PON1 |
| Niu Xi | quercetin | DIO1 |
| Niu Xi | quercetin | PCOLCE |
| Niu Xi | quercetin | NPEPPS |
| Niu Xi | quercetin | HK2 |
| Niu Xi | quercetin | NKX3-1 |
| Niu Xi | quercetin | RASA1 |
| Niu Xi | quercetin | GSTM1 |
| Niu Xi | quercetin | GSTM2 |
| Niu Xi | Inokosterone | AR |
| Niu Xi | Ecdysterone | AR |
| Niu Xi | Cyasterone | PGR |
| Niu Xi | Cyasterone | AR |
| Niu Xi | Cyasterone | NR3C2 |
| Dihuang | sitosterol | PGR |
| Dihuang | sitosterol | NCOA2 |
| Dihuang | sitosterol | NR3C2 |
| Dihuang | Stigmasterol | PGR |
| Dihuang | Stigmasterol | NR3C2 |
| Dihuang | Stigmasterol | NCOA2 |
| Dihuang | Stigmasterol | ADH1C |
| Dihuang | Stigmasterol | IGHG1 |
| Dihuang | Stigmasterol | RXRA |
| Dihuang | Stigmasterol | NCOA1 |
| Dihuang | Stigmasterol | PTGS1 |
| Dihuang | Stigmasterol | PTGS2 |
| Dihuang | Stigmasterol | ADRA2A |
| Dihuang | Stigmasterol | SLC6A2 |
| Dihuang | Stigmasterol | SLC6A3 |
| Dihuang | Stigmasterol | ADRB2 |
| Dihuang | Stigmasterol | AKR1B10 |
| Dihuang | Stigmasterol | PLAU |
| Dihuang | Stigmasterol | LTA4H |
| Dihuang | Stigmasterol | MAOB |
| Dihuang | Stigmasterol | MAOA |
| Dihuang | Stigmasterol | PRKACA |
| Dihuang | Stigmasterol | CTRB1 |
| Dihuang | Stigmasterol | CHRM3 |
| Dihuang | Stigmasterol | CHRM1 |
| Dihuang | Stigmasterol | ADRB1 |
| Dihuang | Stigmasterol | SCN5A |
| Dihuang | Stigmasterol | HTR2A |
| Dihuang | Stigmasterol | ADRA1A |
| Dihuang | Stigmasterol | GABRA3 |
| Dihuang | Stigmasterol | CHRM2 |
| Dihuang | Stigmasterol | ADRA1B |
| Dihuang | Stigmasterol | GABRA1 |
| Dihuang | Stigmasterol | CHRNA7 |
| Dihuang | Gamma-Aminobutyric Acid | CACNA2D1 |
| Dihuang | Gamma-Aminobutyric Acid | PLAT |
| Dihuang | Gamma-Aminobutyric Acid | CACNA1A |
| Dihuang | Gamma-Aminobutyric Acid | GRIN3B |
| Dihuang | Gamma-Aminobutyric Acid | CACNA2D2 |
| Dihuang | Gamma-Aminobutyric Acid | GRIN2A |
| Dihuang | Gamma-Aminobutyric Acid | PLG |
| Dihuang | Gamma-Aminobutyric Acid | CACNA1B |
| Dihuang | Gamma-Aminobutyric Acid | GRIN2C |
| Dihuang | Gamma-Aminobutyric Acid | GRIN2B |
| Dihuang | Gamma-Aminobutyric Acid | GRIN3A |
| Dihuang | Gamma-Aminobutyric Acid | GRIN2D |
| Dihuang | Gamma-Aminobutyric Acid | ADORA1 |
| Dihuang | Gamma-Aminobutyric Acid | GRIN1 |
| Dihuang | Gamma-Aminobutyric Acid | DLL1 |
| Dihuang | Gamma-Aminobutyric Acid | RIPK1 |
| Dihuang | Gamma-Aminobutyric Acid | ASCL1 |
| Dihuang | Gamma-Aminobutyric Acid | EPHX2 |
| Dihuang | Gamma-Aminobutyric Acid | ADA |
| Dihuang | Gamma-Aminobutyric Acid | HAP1 |
| Gouqizi | quercetin | PSMD3 |
| Gouqizi | atropine | HTR1A |
| Gouqizi | atropine | HTR1B |
| Gouqizi | beta-sitosterol | HTR2A |
| Gouqizi | Stigmasterol | HTR2A |
| Gouqizi | atropine | HTR2A |
| Gouqizi | atropine | HTR2C |
| Gouqizi | quercetin | MMP2 |
| Gouqizi | quercetin | HSPA5 |
| Gouqizi | quercetin | ACHE |
| Gouqizi | quercetin | ACACA |
| Gouqizi | quercetin | AHSA1 |
| Gouqizi | Stigmasterol | ADH1C |
| Gouqizi | Sitosterol alpha1 | ADH1C |
| Gouqizi | Stigmasterol | AKR1B10 |
| Gouqizi | quercetin | AKR1B10 |
| Gouqizi | beta-sitosterol | ADRA1A |
| Gouqizi | Stigmasterol | ADRA1A |
| Gouqizi | atropine | ADRA1A |
| Gouqizi | beta-sitosterol | ADRA1B |
| Gouqizi | Stigmasterol | ADRA1B |
| Gouqizi | atropine | ADRA1B |
| Gouqizi | Stigmasterol | ADRA2A |
| Gouqizi | atropine | ADRA2A |
| Gouqizi | atropine | ADRA2B |
| Gouqizi | atropine | ADRA2C |
| Gouqizi | Stigmasterol | MAOA |
| Gouqizi | Stigmasterol | MAOB |
| Gouqizi | quercetin | MAOB |
| Gouqizi | glycitein | APP |
| Gouqizi | glycitein | AR |
| Gouqizi | quercetin | AR |
| Gouqizi | beta-sitosterol | BAX |
| Gouqizi | quercetin | BAX |
| Gouqizi | beta-sitosterol | BCL2 |
| Gouqizi | quercetin | BCL2 |
| Gouqizi | quercetin | ALOX5 |
| Gouqizi | quercetin | AHR |
| Gouqizi | quercetin | ABCG1 |
| Gouqizi | quercetin | BIRC5 |
| Gouqizi | quercetin | BCL2L1 |
| Gouqizi | Stigmasterol | ADRB1 |
| Gouqizi | atropine | ADRB1 |
| Gouqizi | beta-sitosterol | ADRB2 |
| Gouqizi | Stigmasterol | ADRB2 |
| Gouqizi | atropine | ADRB2 |
| Gouqizi | quercetin | ADRB2 |
| Gouqizi | glycitein | CALM1 |
| Gouqizi | 7-O-Methylluteolin-6-C-beta-glucoside_qt | CALM1 |
| Gouqizi | beta-sitosterol | CASP3 |
| Gouqizi | quercetin | CASP3 |
| Gouqizi | beta-sitosterol | CASP8 |
| Gouqizi | quercetin | CASP8 |
| Gouqizi | beta-sitosterol | CASP9 |
| Gouqizi | quercetin | CASP9 |
| Gouqizi | quercetin | CTSD |
| Gouqizi | quercetin | CAV1 |
| Gouqizi | quercetin | CCL2 |
| Gouqizi | quercetin | CD40LG |
| Gouqizi | quercetin | CDK1 |
| Gouqizi | glycitein | CDK2 |
| Gouqizi | quercetin | TP53 |
| Gouqizi | beta-sitosterol | PDE3A |
| Gouqizi | glycitein | PDE3A |
| Gouqizi | Stigmasterol | CTRB1 |
| Gouqizi | quercetin | CLDN4 |
| Gouqizi | quercetin | F7 |
| Gouqizi | quercetin | F10 |
| Gouqizi | quercetin | COL19A1 |
| Gouqizi | quercetin | COL3A1 |
| Gouqizi | glycitein | MMP13 |
| Gouqizi | quercetin | CRP |
| Gouqizi | quercetin | CXCL10 |
| Gouqizi | quercetin | CXCL11 |
| Gouqizi | quercetin | CXCL2 |
| Gouqizi | glycitein | CCNA2 |
| Gouqizi | quercetin | CDKN1A |
| Gouqizi | quercetin | CYP1A1 |
| Gouqizi | quercetin | CYP1A2 |
| Gouqizi | quercetin | CYP1B1 |
| Gouqizi | quercetin | CYP3A4 |
| Gouqizi | Atropine | DRD5 |
| Gouqizi | atropine | DRD2 |
| Gouqizi | quercetin | DCAF5 |
| Gouqizi | atropine | OPRD1 |
| Gouqizi | quercetin | DPP4 |
| Gouqizi | quercetin | TOP2A |
| Gouqizi | quercetin | TOP1 |
| Gouqizi | 7-O-Methylluteolin-6-C-beta-glucoside_qt | TOP2A |
| Gouqizi | Physcion-8-O-beta-D-gentiobioside | TOP2A |
| Gouqizi | beta-sitosterol | DRD5 |
| Gouqizi | quercetin | DUOX2 |
| Gouqizi | quercetin | EGFR |
| Gouqizi | quercetin | SELE |
| Gouqizi | glycitein | ESR1 |
| Gouqizi | glycitein | ESR2 |
| Gouqizi | quercetin | SULT1E1 |
| Gouqizi | quercetin | ELK1 |
| Gouqizi | quercetin | EIF6 |
| Gouqizi | quercetin | CCND1 |
| Gouqizi | quercetin | CCNB1 |
| Gouqizi | beta-sitosterol | GABRA1 |
| Gouqizi | Stigmasterol | GABRA1 |
| Gouqizi | Sitosterol alpha1 | GABRA1 |
| Gouqizi | atropine | GABRA1 |
| Gouqizi | quercetin | GABRA1 |
| Gouqizi | beta-sitosterol | GABRA2 |
| Gouqizi | beta-sitosterol | GABRA3 |
| Gouqizi | Stigmasterol | GABRA3 |
| Gouqizi | quercetin | GJA1 |
| Gouqizi | 6-Fluoroindole-7-Dehydrocholesterol | NR3C1 |
| Gouqizi | quercetin | GSTM1 |
| Gouqizi | quercetin | GSTM2 |
| Gouqizi | quercetin | GSTP1 |
| Gouqizi | glycitein | GSK3B |
| Gouqizi | quercetin | HSF1 |
| Gouqizi | quercetin | HSPB1 |
| Gouqizi | beta-sitosterol | HSP90AB1 |
| Gouqizi | cyanin | HSP90AB1 |
| Gouqizi | glycitein | HSP90AB1 |
| Gouqizi | 7-O-Methylluteolin-6-C-beta-glucoside_qt | HSP90AB1 |
| Gouqizi | quercetin | HSP90AB1 |
| Gouqizi | quercetin | HMOX1 |
| Gouqizi | quercetin | HK2 |
| Gouqizi | atropine | HRH1 |
| Gouqizi | quercetin | NKX3-1 |
| Gouqizi | quercetin | HAS2 |
| Gouqizi | quercetin | HIF1A |
| Gouqizi | Stigmasterol | IGHG1 |
| Gouqizi | quercetin | CHUK |
| Gouqizi | quercetin | INSR |
| Gouqizi | quercetin | IGF2 |
| Gouqizi | quercetin | IGFBP3 |
| Gouqizi | quercetin | ICAM1 |
| Gouqizi | quercetin | IFNG |
| Gouqizi | quercetin | IRF1 |
| Gouqizi | quercetin | IL1A |
| Gouqizi | quercetin | IL1B |
| Gouqizi | quercetin | IL10 |
| Gouqizi | quercetin | IL2 |
| Gouqizi | quercetin | IL6 |
| Gouqizi | quercetin | CXCL8 |
| Gouqizi | quercetin | MMP1 |
| Gouqizi | Stigmasterol | LTA4H |
| Gouqizi | quercetin | MGAM |
| Gouqizi | quercetin | MMP9 |
| Gouqizi | beta-sitosterol | MAP2 |
| Gouqizi | Stigmasterol | NR3C2 |
| Gouqizi | CLR | NR3C2 |
| Gouqizi | Sitosterol alpha1 | NR3C2 |
| Gouqizi | LAN | NR3C2 |
| Gouqizi | Cycloartenol | NR3C2 |
| Gouqizi | 24-methylidenelophenol | NR3C2 |
| Gouqizi | 24-ethylcholesta-5,22-dienol | NR3C2 |
| Gouqizi | Fucosterol | NR3C2 |
| Gouqizi | 31-norlanosterol | NR3C2 |
| Gouqizi | 4alpha,24-dimethylcholesta-7,24-dienol | NR3C2 |
| Gouqizi | 6-Fluoroindole-7-Dehydrocholesterol | NR3C2 |
| Gouqizi | lanost-8-en-3beta-ol | NR3C2 |
| Gouqizi | lanost-8-enol | NR3C2 |
| Gouqizi | Obtusifoliol | NR3C2 |
| Gouqizi | quercetin | MAPK1 |
| Gouqizi | glycitein | MAPK14 |
| Gouqizi | beta-sitosterol | PRKACA |
| Gouqizi | Stigmasterol | PRKACA |
| Gouqizi | glycitein | PRKACA |
| Gouqizi | quercetin | PRKACA |
| Gouqizi | beta-sitosterol | CHRM1 |
| Gouqizi | Stigmasterol | CHRM1 |
| Gouqizi | atropine | CHRM1 |
| Gouqizi | beta-sitosterol | CHRM2 |
| Gouqizi | Stigmasterol | CHRM2 |
| Gouqizi | atropine | CHRM2 |
| Gouqizi | beta-sitosterol | CHRM3 |
| Gouqizi | Stigmasterol | CHRM3 |
| Gouqizi | atropine | CHRM3 |
| Gouqizi | beta-sitosterol | CHRM4 |
| Gouqizi | atropine | CHRM4 |
| Gouqizi | atropine | CHRM5 |
| Gouqizi | beta-sitosterol | OPRM1 |
| Gouqizi | atropine | OPRM1 |
| Gouqizi | quercetin | MYC |
| Gouqizi | quercetin | MPO |
| Gouqizi | quercetin | NQO1 |
| Gouqizi | quercetin | POR |
| Gouqizi | beta-sitosterol | CHRNA7 |
| Gouqizi | Stigmasterol | CHRNA7 |
| Gouqizi | beta-sitosterol | CHRNA2 |
| Gouqizi | glycitein | MMP8 |
| Gouqizi | quercetin | NCF1 |
| Gouqizi | quercetin | NFKBIA |
| Gouqizi | quercetin | NOS3 |
| Gouqizi | glycitein | NOS2 |
| Gouqizi | quercetin | NFE2L2 |
| Gouqizi | Stigmasterol | NCOA1 |
| Gouqizi | glycitein | NCOA1 |
| Gouqizi | beta-sitosterol | NCOA2 |
| Gouqizi | Stigmasterol | NCOA2 |
| Gouqizi | CLR | NCOA2 |
| Gouqizi | Mandenol | NCOA2 |
| Gouqizi | Ethyl linolenate | NCOA2 |
| Gouqizi | LAN | NCOA2 |
| Gouqizi | 24-methylidenelophenol | NCOA2 |
| Gouqizi | daucosterol_qt | NCOA2 |
| Gouqizi | 14b-pregnane | NCOA2 |
| Gouqizi | Fucosterol | NCOA2 |
| Gouqizi | 31-norlanosterol | NCOA2 |
| Gouqizi | 4alpha,24-dimethylcholesta-7,24-dienol | NCOA2 |
| Gouqizi | 7-O-Methylluteolin-6-C-beta-glucoside_qt | NCOA2 |
| Gouqizi | (E,E)-1-ethyl octadeca-3,13-dienoate | NCOA2 |
| Gouqizi | Obtusifoliol | NCOA2 |
| Gouqizi | quercetin | NCOA2 |
| Gouqizi | quercetin | NR1I2 |
| Gouqizi | quercetin | NR1I3 |
| Gouqizi | quercetin | ODC1 |
| Gouqizi | quercetin | SPP1 |
| Gouqizi | glycitein | PPARG |
| Gouqizi | quercetin | PPARG |
| Gouqizi | quercetin | PPARA |
| Gouqizi | quercetin | PPARD |
| Gouqizi | quercetin | PTEN |
| Gouqizi | beta-sitosterol | PIK3CG |
| Gouqizi | quercetin | PIK3CG |
| Gouqizi | quercetin | SERPINE1 |
| Gouqizi | quercetin | TNKS |
| Gouqizi | beta-sitosterol | KCNH2 |
| Gouqizi | quercetin | KCNH2 |
| Gouqizi | quercetin | PCOLCE |
| Gouqizi | quercetin | EGF |
| Gouqizi | beta-sitosterol | PGR |
| Gouqizi | Stigmasterol | PGR |
| Gouqizi | CLR | PGR |
| Gouqizi | Sitosterol alpha1 | PGR |
| Gouqizi | LAN | PGR |
| Gouqizi | campesterol | PGR |
| Gouqizi | 24-methylidenelophenol | PGR |
| Gouqizi | daucosterol_qt | PGR |
| Gouqizi | 14b-pregnane | PGR |
| Gouqizi | 24-ethylcholest-22-enol | PGR |
| Gouqizi | 24-ethylcholesta-5,22-dienol | PGR |
| Gouqizi | 24-methyl-31-norlanost-9(11)-enol | PGR |
| Gouqizi | 24-methylenelanost-8-enol | PGR |
| Gouqizi | Fucosterol | PGR |
| Gouqizi | 31-norlanost-9(11)-enol | PGR |
| Gouqizi | 31-norlanosterol | PGR |
| Gouqizi | 4,24-methyllophenol | PGR |
| Gouqizi | Lophenol | PGR |
| Gouqizi | 4alpha,14alpha,24-trimethylcholesta-8,24-dienol | PGR |
| Gouqizi | 4alpha,24-dimethylcholesta-7,24-dienol | PGR |
| Gouqizi | 4alpha-methyl-24-ethylcholesta-7,24-dienol | PGR |
| Gouqizi | 6-Fluoroindole-7-Dehydrocholesterol | PGR |
| Gouqizi | lanost-8-en-3beta-ol | PGR |
| Gouqizi | lanost-8-enol | PGR |
| Gouqizi | Obtusifoliol | PGR |
| Gouqizi | quercetin | PTGER3 |
| Gouqizi | beta-sitosterol | PTGS1 |
| Gouqizi | Stigmasterol | PTGS1 |
| Gouqizi | Mandenol | PTGS1 |
| Gouqizi | Ethyl linolenate | PTGS1 |
| Gouqizi | glycitein | PTGS1 |
| Gouqizi | quercetin | PTGS1 |
| Gouqizi | beta-sitosterol | PTGS2 |
| Gouqizi | Stigmasterol | PTGS2 |
| Gouqizi | Sitosterol alpha1 | PTGS2 |
| Gouqizi | Mandenol | PTGS2 |
| Gouqizi | cyanin | PTGS2 |
| Gouqizi | glycitein | PTGS2 |
| Gouqizi | 14b-pregnane | PTGS2 |
| Gouqizi | 7-O-Methylluteolin-6-C-beta-glucoside_qt | PTGS2 |
| Gouqizi | quercetin | PTGS2 |
| Gouqizi | quercetin | ACP3 |
| Gouqizi | quercetin | RUNX1T1 |
| Gouqizi | beta-sitosterol | PRKCA |
| Gouqizi | quercetin | PRKCA |
| Gouqizi | quercetin | PRKCB |
| Gouqizi | quercetin | FOS |
| Gouqizi | glycitein | PIM1 |
| Gouqizi | quercetin | NPEPPS |
| Gouqizi | quercetin | AKT1 |
| Gouqizi | quercetin | RAF1 |
| Gouqizi | quercetin | RASSF1 |
| Gouqizi | quercetin | RASA1 |
| Gouqizi | quercetin | ERBB2 |
| Gouqizi | quercetin | ERBB3 |
| Gouqizi | quercetin | RB1 |
| Gouqizi | Stigmasterol | RXRA |
| Gouqizi | glycitein | RXRA |
| Gouqizi | quercetin | RXRA |
| Gouqizi | quercetin | RUNX2 |
| Gouqizi | glycitein | CHEK1 |
| Gouqizi | quercetin | CHEK2 |
| Gouqizi | beta-sitosterol | PON1 |
| Gouqizi | quercetin | PON1 |
| Gouqizi | quercetin | STAT1 |
| Gouqizi | beta-sitosterol | SCN5A |
| Gouqizi | Stigmasterol | SCN5A |
| Gouqizi | Atropine | SCN5A |
| Gouqizi | quercetin | SCN5A |
| Gouqizi | Stigmasterol | SLC6A3 |
| Gouqizi | atropine | SLC6A3 |
| Gouqizi | Stigmasterol | SLC6A2 |
| Gouqizi | atropine | SLC6A2 |
| Gouqizi | beta-sitosterol | SLC6A4 |
| Gouqizi | atropine | SLC6A4 |
| Gouqizi | quercetin | SLC2A4 |
| Gouqizi | quercetin | MMP3 |
| Gouqizi | quercetin | SOD1 |
| Gouqizi | quercetin | F2 |
| Gouqizi | quercetin | THBD |
| Gouqizi | quercetin | F3 |
| Gouqizi | quercetin | PLAT |
| Gouqizi | beta-sitosterol | JUN |
| Gouqizi | quercetin | JUN |
| Gouqizi | quercetin | E2F1 |
| Gouqizi | quercetin | E2F2 |
| Gouqizi | quercetin | RELA |
| Gouqizi | beta-sitosterol | TGFB1 |
| Gouqizi | quercetin | TGFB1 |
| Gouqizi | glycitein | PRSS1 |
| Gouqizi | quercetin | PRSS1 |
| Gouqizi | quercetin | TNF |
| Gouqizi | quercetin | DIO1 |
| Gouqizi | Stigmasterol | PLAU |
| Gouqizi | quercetin | PLAU |
| Gouqizi | quercetin | VCAM1 |
| Gouqizi | quercetin | VEGFA |
| Gouqizi | quercetin | XDH |
| Gouqizi | (24r)-4alpha-Methyl-24-Ethylcholesta-7,25-Dien-3beta-Yl Acetate | AR |
| Gouqizi | 31-Norcyclolaudenol | VDR |
| Gouqizi | 31-Norcyclolaudenol | CYP27B1 |
| Gouqizi | 31-Norcyclolaudenol | GC |
| Gouqizi | 31-Norcyclolaudenol | SNW1 |
| Gouqizi | Cycloartanol | TRPV3 |
| Gouqizi | Cycloartanol | OPRK1 |
| Gouqizi | Cycloartanol | TRPM8 |
| Gouqizi | Cycloartanol | TRPA1 |
| Gouqizi | Cycloartanol | KCNK4 |
| Gouqizi | Lupeol | VDR |
| Gouqizi | Lupeol | CYP27B1 |
| Gouqizi | Lupeol | GC |
| Gouqizi | Lupeol | SNW1 |
| Gouqizi | Vitamin B1 | SLC19A2 |
| Gouqizi | Vitamin B1 | TPK1 |
| Gouqizi | Vitamin B1 | THTPA |
| Guiban | Aspartic Acid | GOT2 |
| Guiban | Aspartic Acid | SLC1A1 |
| Guiban | Aspartic Acid | ACY3 |
| Guiban | Aspartic Acid | ASPA |
| Guiban | Aspartic Acid | ADSSL1 |
| Guiban | Aspartic Acid | ASNS |
| Guiban | Aspartic Acid | DARS2 |
| Guiban | Aspartic Acid | GOT1 |
| Guiban | Aspartic Acid | ASPH |
| Guiban | Aspartic Acid | SLC25A12 |
| Guiban | Aspartic Acid | DARS |
| Guiban | Aspartic Acid | ASRGL1 |
| Guiban | Aspartic Acid | RNASE1 |
| Guiban | Aspartic Acid | ASS1 |
| Guiban | Aspartic Acid | ADSS |
| Guiban | Aspartic Acid | CAD |
| Guiban | Aspartic Acid | ACY1 |
| Guiban | Aspartic Acid | SLC25A13 |
| Guiban | Aspartic Acid | LYZ |
| Guiban | Aspartic Acid | PAICS |
| Guiban | Methionine | BHMT2 |
| Guiban | Methionine | MTRR |
| Guiban | Methionine | METAP2 |
| Guiban | Methionine | MTR |
| Guiban | Methionine | BHMT |
| Guiban | Methionine | GSTP1 |
| Guiban | Methionine | TRAF2 |
| Guiban | Methionine | CBS |
| Guiban | Methionine | COMT |
| Guiban | Calcium Carbonate | FDPS |
| Guiban | Phenylalanine | SLC7A8 |
| Guiban | Phenylalanine | TH |
| Guiban | Phenylalanine | TAT |
| Guiban | Phenylalanine | FARS2 |
| Guiban | Phenylalanine | PAH |
| Guiban | Phenylalanine | FARSA |
| Guiban | Phenylalanine | FARSB |
| Guiban | Phenylalanine | YARS |
| Guiban | Phenylalanine | YARS2 |
| Guiban | Phenylalanine | BCO2 |
| Guiban | Phenylalanine | PNMT |
| Guiban | Phenylalanine | DDC |
| Lujiao | Calcium Carbonate | FDPS |
| Shanyao | Isofucosterol | ABAT |
| Shanyao | Stigmasterol | HTR2A |
| Shanyao | Kadsurenone | ACHE |
| Shanyao | Isofucosterol | ADH1A |
| Shanyao | Isofucosterol | ADH1B |
| Shanyao | Isofucosterol | ADH1C |
| Shanyao | Stigmasterol | ADH1C |
| Shanyao | Stigmasterol | AKR1B10 |
| Shanyao | Stigmasterol | ADRA1A |
| Shanyao | piperlonguminine | ADRA1B |
| Shanyao | Stigmasterol | ADRA1B |
| Shanyao | Kadsurenone | ADRA1B |
| Shanyao | hancinone C | ADRA1B |
| Shanyao | Kadsurenone | ADRA1D |
| Shanyao | hancinone C | ADRA1D |
| Shanyao | Stigmasterol | ADRA2A |
| Shanyao | Stigmasterol | MAOA |
| Shanyao | piperlonguminine | MAOB |
| Shanyao | Stigmasterol | MAOB |
| Shanyao | AIDS180907 | AR |
| Shanyao | Stigmasterol | ADRB1 |
| Shanyao | piperlonguminine | ADRB2 |
| Shanyao | Stigmasterol | ADRB2 |
| Shanyao | Kadsurenone | ADRB2 |
| Shanyao | Kadsurenone | BACE1 |
| Shanyao | Kadsurenone | KCNMA1 |
| Shanyao | hancinone C | KCNMA1 |
| Shanyao | AIDS180907 | CALM1 |
| Shanyao | Kadsurenone | CALM1 |
| Shanyao | hancinone C | CALM1 |
| Shanyao | diosgenin | ABCC2 |
| Shanyao | Kadsurenone | CA2 |
| Shanyao | diosgenin | CAT |
| Shanyao | diosgenin | TP53 |
| Shanyao | piperlonguminine | PDE3A |
| Shanyao | Kadsurenone | PDE3A |
| Shanyao | hancinone C | PDE3A |
| Shanyao | Stigmasterol | CTRB1 |
| Shanyao | AIDS180907 | F7 |
| Shanyao | hancinone C | F7 |
| Shanyao | AIDS180907 | F10 |
| Shanyao | Kadsurenone | F10 |
| Shanyao | hancinone C | F10 |
| Shanyao | AIDS180907 | CCNA2 |
| Shanyao | diosgenin | CDKN1A |
| Shanyao | diosgenin | PLA2G4A |
| Shanyao | Kadsurenone | DPP4 |
| Shanyao | hancinone C | DPP4 |
| Shanyao | AIDS180907 | TOP2A |
| Shanyao | Kadsurenone | TOP2A |
| Shanyao | hancinone C | TOP2A |
| Shanyao | AIDS180907 | ESR1 |
| Shanyao | hancinone C | ESR1 |
| Shanyao | hancinone C | ESR2 |
| Shanyao | diosgenin | FASN |
| Shanyao | Isofucosterol | GABRA1 |
| Shanyao | Stigmasterol | GABRA1 |
| Shanyao | Stigmasterol | GABRA3 |
| Shanyao | Dioscoreside C_qt | NR3C1 |
| Shanyao | AIDS180907 | GSK3B |
| Shanyao | Kadsurenone | HSP90AB1 |
| Shanyao | hancinone C | HSP90AB1 |
| Shanyao | (-)-taxifolin | HSP90AB1 |
| Shanyao | diosgenin | HIF1A |
| Shanyao | Stigmasterol | IGHG1 |
| Shanyao | piperlonguminine | LTA4H |
| Shanyao | Stigmasterol | LTA4H |
| Shanyao | Isofucosterol | SPACA3 |
| Shanyao | CLR | NR3C2 |
| Shanyao | Isofucosterol | NR3C2 |
| Shanyao | Stigmasterol | NR3C2 |
| Shanyao | diosgenin | NR3C2 |
| Shanyao | Stigmasterol | PRKACA |
| Shanyao | piperlonguminine | CHRM1 |
| Shanyao | Stigmasterol | CHRM1 |
| Shanyao | Kadsurenone | CHRM1 |
| Shanyao | hancinone C | CHRM1 |
| Shanyao | Stigmasterol | CHRM2 |
| Shanyao | Kadsurenone | CHRM2 |
| Shanyao | Stigmasterol | CHRM3 |
| Shanyao | Kadsurenone | CHRM3 |
| Shanyao | hancinone C | CHRM3 |
| Shanyao | Kadsurenone | CHRM5 |
| Shanyao | Kadsurenone | OPRM1 |
| Shanyao | Stigmasterol | CHRNA7 |
| Shanyao | AIDS180907 | NOS2 |
| Shanyao | hancinone C | NOS2 |
| Shanyao | piperlonguminine | NOS3 |
| Shanyao | Stigmasterol | NCOA1 |
| Shanyao | AIDS180907 | NCOA1 |
| Shanyao | Kadsurenone | NCOA1 |
| Shanyao | hancinone C | NCOA1 |
| Shanyao | Dioscoreside C_qt | NCOA2 |
| Shanyao | CLR | NCOA2 |
| Shanyao | Isofucosterol | NCOA2 |
| Shanyao | Stigmasterol | NCOA2 |
| Shanyao | AIDS180907 | NCOA2 |
| Shanyao | Kadsurenone | NCOA2 |
| Shanyao | hancinone C | NCOA2 |
| Shanyao | diosgenin | NR1I2 |
| Shanyao | (-)-taxifolin | PIK3CG |
| Shanyao | Kadsurenone | KCNH2 |
| Shanyao | hancinone C | KCNH2 |
| Shanyao | 24-Methylcholest-5-enyl-3belta-O-glucopyranoside_qt | PGR |
| Shanyao | campesterol | PGR |
| Shanyao | CLR | PGR |
| Shanyao | Isofucosterol | PGR |
| Shanyao | Stigmasterol | PGR |
| Shanyao | diosgenin | PGR |
| Shanyao | Stigmasterol | PTGS1 |
| Shanyao | Kadsurenone | PTGS1 |
| Shanyao | (-)-taxifolin | PTGS1 |
| Shanyao | Stigmasterol | PTGS2 |
| Shanyao | AIDS180907 | PTGS2 |
| Shanyao | Kadsurenone | PTGS2 |
| Shanyao | hancinone C | PTGS2 |
| Shanyao | (-)-taxifolin | PTGS2 |
| Shanyao | diosgenin | PTGS2 |
| Shanyao | AIDS180907 | PIM1 |
| Shanyao | diosgenin | AKT1 |
| Shanyao | piperlonguminine | RXRA |
| Shanyao | Stigmasterol | RXRA |
| Shanyao | Kadsurenone | RXRA |
| Shanyao | diosgenin | MTOR |
| Shanyao | piperlonguminine | SAA1 |
| Shanyao | Stigmasterol | SCN5A |
| Shanyao | Kadsurenone | SCN5A |
| Shanyao | hancinone C | SCN5A |
| Shanyao | piperlonguminine | SLC6A3 |
| Shanyao | Stigmasterol | SLC6A3 |
| Shanyao | Stigmasterol | SLC6A2 |
| Shanyao | piperlonguminine | SLC6A4 |
| Shanyao | diosgenin | SOD1 |
| Shanyao | Kadsurenone | F2 |
| Shanyao | hancinone C | F2 |
| Shanyao | diosgenin | RELA |
| Shanyao | Kadsurenone | PRSS1 |
| Shanyao | hancinone C | PRSS1 |
| Shanyao | Stigmasterol | PLAU |
| Shanyao | diosgenin | VEGFA |
| Shanyao | Dihydropinosylvin | GABRA2 |
| Shanyao | Dihydropinosylvin | GABRD |
| Shanyao | Dihydropinosylvin | PDE3A |
| Shanyao | Dihydropinosylvin | GABRB1 |
| Shanyao | Dihydropinosylvin | GABRG3 |
| Shanyao | Dihydropinosylvin | TNF |
| Shanyao | Dihydropinosylvin | GABRE |
| Shanyao | Dihydropinosylvin | SCN2A |
| Shanyao | Dihydropinosylvin | GABRA3 |
| Shanyao | Dihydropinosylvin | GABRG1 |
| Shanyao | Dihydropinosylvin | GABRP |
| Shanyao | Dihydropinosylvin | GABRA4 |
| Shanyao | Dihydropinosylvin | GABRB2 |
| Shanyao | Dihydropinosylvin | ACHE |
| Shanyao | Dihydropinosylvin | SCN4A |
| Shanyao | Dihydropinosylvin | GABRQ |
| Shanyao | Dihydropinosylvin | GABRB3 |
| Shanyao | Dihydropinosylvin | GABRA5 |
| Shanyao | Dihydropinosylvin | GABRA6 |
| Shanyao | Dihydropinosylvin | GABRA1 |
| Shanyao | Dihydropinosylvin | PDE4B |
| Shanyao | Dihydropinosylvin | BCHE |
| Shanyao | Dihydropinosylvin | GABRG2 |
| Shanyao | Dihydropinosylvin | PDE3B |
| Shanyao | Dihydropinosylvin | PDE4D |
| Shanyao | Dihydropinosylvin | COLQ |
| Shanyao | Dihydropinosylvin | LTA |
| Shanyao | Dihydropinosylvin | RXRA |
| Shanyao | Allantoin | PDE4A |
| Shanyao | Allantoin | GABRA2 |
| Shanyao | Allantoin | GABRD |
| Shanyao | Allantoin | CHRNA4 |
| Shanyao | Allantoin | GABRB1 |
| Shanyao | Allantoin | ADORA2A |
| Shanyao | Allantoin | GABRG3 |
| Shanyao | Allantoin | GRIN3B |
| Shanyao | Allantoin | CHRNA7 |
| Shanyao | Allantoin | GRIN2A |
| Shanyao | Allantoin | GABRE |
| Shanyao | Allantoin | GABRA3 |
| Shanyao | Allantoin | ADORA2B |
| Shanyao | Allantoin | GABRG1 |
| Shanyao | Allantoin | GABRP |
| Shanyao | Allantoin | GRIA2 |
| Shanyao | Allantoin | GABRA4 |
| Shanyao | Allantoin | GABRB2 |
| Shanyao | Allantoin | GRIN2C |
| Shanyao | Allantoin | GRIN2B |
| Shanyao | Allantoin | GABRQ |
| Shanyao | Allantoin | ADORA3 |
| Shanyao | Allantoin | GABRB3 |
| Shanyao | Allantoin | GRIN3A |
| Shanyao | Allantoin | GABRA5 |
| Shanyao | Allantoin | GABRA6 |
| Shanyao | Allantoin | GABRA1 |
| Shanyao | Allantoin | PDE4B |
| Shanyao | Allantoin | GABRG2 |
| Shanyao | Allantoin | GRIN2D |
| Shanyao | Allantoin | ADORA1 |
| Shanyao | Allantoin | CHRFAM7A |
| Shanyao | Allantoin | GRIK2 |
| Shanyao | Allantoin | GRIN1 |
| Shanyao | Allantoin | RIPK1 |
| Shanyao | Allantoin | PDE4D |
| Shanyao | Allantoin | ADRA1B |
| Shanyao | Allantoin | TACR2 |
| Shanyao | Allantoin | EPHX2 |
| Shanyao | Allantoin | CX3CR1 |
| Shanyao | Allantoin | ADA |
| Shanyao | Allantoin | CHRNA9 |
| Shanyao | Allantoin | HAP1 |
| Shanyao | Phytocassane A | AR |
| Shanyao | Phytocassane A | NR3C1 |
| Shanyao | Phytocassane A | ANXA1 |
| Shanyao | Phytocassane A | ATP1A1 |
| Shanyao | Phytocassane A | NOTCH2 |
| Shanyao | Dopamine | SLC6A3 |
| Shanyao | Dopamine | DRD2 |
| Shanyao | Dopamine | SLC6A4 |
| Shanyao | Dopamine | DRD4 |
| Shanyao | Dopamine | HTR7 |
| Shanyao | Dopamine | HTR1A |
| Shanyao | Dopamine | DBH |
| Shanyao | Dopamine | DRD3 |
| Shanyao | Dopamine | DRD5 |
| Shanyao | Dopamine | DRD1 |
| Shanyao | Dopamine | SLC6A2 |
| Shanyao | Dopamine | ADRA1A |
| Shanyao | Dopamine | ADRB2 |
| Shanyao | Dopamine | ADRA2A |
| Shanyao | Dopamine | ADRB1 |
| Shanyao | Dopamine | SLC18A1 |
| Shanyao | Dopamine | ADRA1D |
| Shanyao | Dopamine | PAH |
| Shanyao | Dopamine | SLC18A2 |
| Shanyao | Dopamine | ADRA1B |
| Shanyao | Dopamine | ADRA2C |
| Shanyao | Dopamine | ADRB3 |
| Shanyao | Dopamine | ADRA2B |
| Shanyao | Dopamine | DNMT3A |
| Shanyao | Dopamine | HTR1B |
| Shanyao | Dopamine | CEND1 |
| Shanyao | Dopamine | CNR1 |
| Shanyao | Dopamine | SLC22A1 |
| Shanyao | Dopamine | MC3R |
| Shanyao | Dopamine | SLC9A3R1 |
| Shanyao | Dopamine | CD34 |
| Shanyao | Batatasin Iii | TYR |
| Shanyao | Batatasin Iii | DCT |
| Shanyao | Batatasin Iii | TYRP1 |
| Shanyao | Dioscoreside C | POLA1 |
| Shanyao | Dihydroquercetin | SOAT1 |
| Shanyao | Dihydroquercetin | MTTP |
| Shanyao | Dihydroquercetin | SOAT2 |
| Shanyao | Batatasin I | TYR |
| Shanyao | Batatasin I | DCT |
| Shanyao | Batatasin I | TYRP1 |
| Shanyao | Cholesterol | VDR |
| Shanyao | Cholesterol | CYP27B1 |
| Shanyao | Cholesterol | GC |
| Shanyao | Cholesterol | SNW1 |
| Shanyao | Campesterol | ESR1 |
| Shanyao | Batatasin Iv | TYR |
| Shanyao | Batatasin Iv | DCT |
| Shanyao | Batatasin Iv | TYRP1 |
| Shanyao | Stigmasterol | KCND1 |
| Shanyao | Stigmasterol | KCNA3 |
| Shanyao | Stigmasterol | PRKAB1 |
| Shanyao | Stigmasterol | ADH1A |
| Shanyao | Stigmasterol | KCNA10 |
| Shanyao | Stigmasterol | GAMT |
| Shanyao | Stigmasterol | KCNC3 |
| Shanyao | Stigmasterol | KCNA1 |
| Shanyao | Stigmasterol | KCNA2 |
| Shanyao | Stigmasterol | TPO |
| Shanyao | Stigmasterol | CAT |
| Shanyao | Stigmasterol | KCNB1 |
| Shanyao | Stigmasterol | ADH1B |
| Shanyao | Stigmasterol | DLG4 |
| Shanyao | Stigmasterol | KCNC2 |
| Shanyao | Stigmasterol | KCNC1 |
| Shanyao | Stigmasterol | KCNB2 |
| Shanyao | Stigmasterol | KCNA5 |
| Shanyao | Stigmasterol | RNASE1 |
| Shanyao | Stigmasterol | KCND2 |
| Shanyao | Stigmasterol | ALDH2 |
| Shanyao | Stigmasterol | KCNA4 |
| Shanyao | Stigmasterol | KCNA7 |
| Shanyao | Stigmasterol | KCNA6 |
| Shanyao | Stigmasterol | KCND3 |
| Shanyao | Stigmasterol | GUCY1B3 |
| Shanyao | Stigmasterol | GATM |
| Shanyao | Stigmasterol | KCNK4 |
| Shanyao | Stigmasterol | IYD |
| Shanyao | Stigmasterol | KCNQ1 |
| Shanzhuyu | Tetrahydroalstonine | HTR2A |
| Shanzhuyu | beta-sitosterol | HTR2A |
| Shanzhuyu | Stigmasterol | HTR2A |
| Shanzhuyu | Tetrahydroalstonine | ACHE |
| Shanzhuyu | Stigmasterol | ADH1C |
| Shanzhuyu | Stigmasterol | AKR1B10 |
| Shanzhuyu | beta-sitosterol | ADRA1A |
| Shanzhuyu | Stigmasterol | ADRA1A |
| Shanzhuyu | Tetrahydroalstonine | ADRA1B |
| Shanzhuyu | beta-sitosterol | ADRA1B |
| Shanzhuyu | Stigmasterol | ADRA1B |
| Shanzhuyu | Tetrahydroalstonine | ADRA1D |
| Shanzhuyu | Stigmasterol | ADRA2A |
| Shanzhuyu | Tetrahydroalstonine | ADRA2C |
| Shanzhuyu | Stigmasterol | MAOA |
| Shanzhuyu | Stigmasterol | MAOB |
| Shanzhuyu | Tetrahydroalstonine | AR |
| Shanzhuyu | beta-sitosterol | BAX |
| Shanzhuyu | beta-sitosterol | BCL2 |
| Shanzhuyu | Stigmasterol | ADRB1 |
| Shanzhuyu | Tetrahydroalstonine | ADRB2 |
| Shanzhuyu | beta-sitosterol | ADRB2 |
| Shanzhuyu | Diop | ADRB2 |
| Shanzhuyu | Stigmasterol | ADRB2 |
| Shanzhuyu | Tetrahydroalstonine | CALM1 |
| Shanzhuyu | Hydroxygenkwanin | CALM1 |
| Shanzhuyu | beta-sitosterol | CASP3 |
| Shanzhuyu | beta-sitosterol | CASP8 |
| Shanzhuyu | beta-sitosterol | CASP9 |
| Shanzhuyu | beta-sitosterol | PDE3A |
| Shanzhuyu | Stigmasterol | CTRB1 |
| Shanzhuyu | Tetrahydroalstonine | F10 |
| Shanzhuyu | Tetrahydroalstonine | OPRD1 |
| Shanzhuyu | Tetrahydroalstonine | DPP4 |
| Shanzhuyu | Hydroxygenkwanin | DPP4 |
| Shanzhuyu | Leucanthoside | TOP2A |
| Shanzhuyu | Tetrahydroalstonine | DRD5 |
| Shanzhuyu | beta-sitosterol | DRD5 |
| Shanzhuyu | beta-sitosterol | GABRA1 |
| Shanzhuyu | Stigmasterol | GABRA1 |
| Shanzhuyu | beta-sitosterol | GABRA2 |
| Shanzhuyu | beta-sitosterol | GABRA3 |
| Shanzhuyu | Stigmasterol | GABRA3 |
| Shanzhuyu | Telocinobufagin | NR3C1 |
| Shanzhuyu | Tetrahydroalstonine | HSP90AB1 |
| Shanzhuyu | Hydroxygenkwanin | HSP90AB1 |
| Shanzhuyu | beta-sitosterol | HSP90AB1 |
| Shanzhuyu | Stigmasterol | IGHG1 |
| Shanzhuyu | Stigmasterol | LTA4H |
| Shanzhuyu | beta-sitosterol | MAP2 |
| Shanzhuyu | sitosterol | NR3C2 |
| Shanzhuyu | Stigmasterol | NR3C2 |
| Shanzhuyu | Telocinobufagin | NR3C2 |
| Shanzhuyu | Tetrahydroalstonine | PRKACA |
| Shanzhuyu | Hydroxygenkwanin | PRKACA |
| Shanzhuyu | beta-sitosterol | PRKACA |
| Shanzhuyu | Stigmasterol | PRKACA |
| Shanzhuyu | Leucanthoside | PTPN1 |
| Shanzhuyu | Tetrahydroalstonine | CHRM1 |
| Shanzhuyu | beta-sitosterol | CHRM1 |
| Shanzhuyu | Stigmasterol | CHRM1 |
| Shanzhuyu | beta-sitosterol | CHRM2 |
| Shanzhuyu | Stigmasterol | CHRM2 |
| Shanzhuyu | Tetrahydroalstonine | CHRM3 |
| Shanzhuyu | beta-sitosterol | CHRM3 |
| Shanzhuyu | Diop | CHRM3 |
| Shanzhuyu | Stigmasterol | CHRM3 |
| Shanzhuyu | Tetrahydroalstonine | CHRM4 |
| Shanzhuyu | beta-sitosterol | CHRM4 |
| Shanzhuyu | Tetrahydroalstonine | CHRM5 |
| Shanzhuyu | Tetrahydroalstonine | OPRM1 |
| Shanzhuyu | beta-sitosterol | OPRM1 |
| Shanzhuyu | beta-sitosterol | CHRNA7 |
| Shanzhuyu | Stigmasterol | CHRNA7 |
| Shanzhuyu | beta-sitosterol | CHRNA2 |
| Shanzhuyu | Tetrahydroalstonine | NOS2 |
| Shanzhuyu | Hydroxygenkwanin | NOS2 |
| Shanzhuyu | Stigmasterol | NCOA1 |
| Shanzhuyu | Ethyl oleate (NF) | NCOA2 |
| Shanzhuyu | Hydroxygenkwanin | NCOA2 |
| Shanzhuyu | sitosterol | NCOA2 |
| Shanzhuyu | poriferast-5-en-3beta-ol | NCOA2 |
| Shanzhuyu | beta-sitosterol | NCOA2 |
| Shanzhuyu | Cornudentanone | NCOA2 |
| Shanzhuyu | Mandenol | NCOA2 |
| Shanzhuyu | Stigmasterol | NCOA2 |
| Shanzhuyu | Ethyl linolenate | NCOA2 |
| Shanzhuyu | Tetrahydroalstonine | PPARG |
| Shanzhuyu | Hydroxygenkwanin | PIK3CG |
| Shanzhuyu | beta-sitosterol | PIK3CG |
| Shanzhuyu | Tetrahydroalstonine | KCNH2 |
| Shanzhuyu | beta-sitosterol | KCNH2 |
| Shanzhuyu | sitosterol | PGR |
| Shanzhuyu | poriferast-5-en-3beta-ol | PGR |
| Shanzhuyu | beta-sitosterol | PGR |
| Shanzhuyu | Stigmasterol | PGR |
| Shanzhuyu | Tetrahydroalstonine | PTGS1 |
| Shanzhuyu | Hydroxygenkwanin | PTGS1 |
| Shanzhuyu | beta-sitosterol | PTGS1 |
| Shanzhuyu | Mandenol | PTGS1 |
| Shanzhuyu | Stigmasterol | PTGS1 |
| Shanzhuyu | Ethyl linolenate | PTGS1 |
| Shanzhuyu | Tetrahydroalstonine | PTGS2 |
| Shanzhuyu | 2,6,10,14,18-pentamethylicosa-2,6,10,14,18-pentaene | PTGS2 |
| Shanzhuyu | Hydroxygenkwanin | PTGS2 |
| Shanzhuyu | beta-sitosterol | PTGS2 |
| Shanzhuyu | Cornudentanone | PTGS2 |
| Shanzhuyu | Mandenol | PTGS2 |
| Shanzhuyu | Stigmasterol | PTGS2 |
| Shanzhuyu | beta-sitosterol | PRKCA |
| Shanzhuyu | Leucanthoside | PIM1 |
| Shanzhuyu | Stigmasterol | RXRA |
| Shanzhuyu | beta-sitosterol | PON1 |
| Shanzhuyu | Tetrahydroalstonine | SCN5A |
| Shanzhuyu | beta-sitosterol | SCN5A |
| Shanzhuyu | Diop | SCN5A |
| Shanzhuyu | Stigmasterol | SCN5A |
| Shanzhuyu | Stigmasterol | SLC6A3 |
| Shanzhuyu | Stigmasterol | SLC6A2 |
| Shanzhuyu | Tetrahydroalstonine | SLC6A4 |
| Shanzhuyu | beta-sitosterol | SLC6A4 |
| Shanzhuyu | Tetrahydroalstonine | F2 |
| Shanzhuyu | Cornudentanone | F2 |
| Shanzhuyu | beta-sitosterol | JUN |
| Shanzhuyu | beta-sitosterol | TGFB1 |
| Shanzhuyu | Tetrahydroalstonine | PRSS1 |
| Shanzhuyu | Hydroxygenkwanin | PRSS1 |
| Shanzhuyu | Stigmasterol | PLAU |
| Shanzhuyu | Elemol | ESR1 |
| Shanzhuyu | Elemol | PGR |
| Shanzhuyu | Elemol | WNT4 |
| Shanzhuyu | 20-Hexadecanoylingenol | PRKCA |
| Shanzhuyu | 20-Hexadecanoylingenol | PRKCD |
| Shanzhuyu | Vitamin B1 | SLC19A2 |
| Shanzhuyu | Vitamin B1 | TPK1 |
| Shanzhuyu | Vitamin B1 | THTPA |
| Shanzhuyu | Benzyl Cinnamate | GABRA2 |
| Shanzhuyu | Benzyl Cinnamate | GABRD |
| Shanzhuyu | Benzyl Cinnamate | GABRB1 |
| Shanzhuyu | Benzyl Cinnamate | GABRG3 |
| Shanzhuyu | Benzyl Cinnamate | GABRE |
| Shanzhuyu | Benzyl Cinnamate | GABRA3 |
| Shanzhuyu | Benzyl Cinnamate | GABRG1 |
| Shanzhuyu | Benzyl Cinnamate | GABRP |
| Shanzhuyu | Benzyl Cinnamate | GABRA4 |
| Shanzhuyu | Benzyl Cinnamate | GABRB2 |
| Shanzhuyu | Benzyl Cinnamate | GABRQ |
| Shanzhuyu | Benzyl Cinnamate | GABRB3 |
| Shanzhuyu | Benzyl Cinnamate | GABRA5 |
| Shanzhuyu | Benzyl Cinnamate | GABRA6 |
| Shanzhuyu | Benzyl Cinnamate | GABRA1 |
| Shanzhuyu | Benzyl Cinnamate | GABRG2 |
| Shanzhuyu | Benzyl Cinnamate | ADRA2B |
| Shanzhuyu | Elemicin | PDE4A |
| Shanzhuyu | Elemicin | PDE3A |
| Shanzhuyu | Elemicin | CACNA1C |
| Shanzhuyu | Elemicin | TRDMT1 |
| Shanzhuyu | Elemicin | DHFRL1 |
| Shanzhuyu | Elemicin | DHFR |
| Shanzhuyu | Elemicin | PDE10A |
| Shanzhuyu | Elemicin | PDE2A |
| Shanzhuyu | Elemicin | TYMS |
| Shanzhuyu | Elemicin | SHMT1 |
| Shanzhuyu | Elemicin | PDE3B |
| Shanzhuyu | Elemicin | DMTN |
| Shanzhuyu | Elemicin | KCNMA1 |
| Shanzhuyu | 3,4-Dehydrolycopen-16-Al | ACHE |
| Shanzhuyu | 3,4-Dehydrolycopen-16-Al | BCHE |
| Shanzhuyu | 3,4-Dehydrolycopen-16-Al | COLQ |
| Shanzhuyu | Alpha-Corocalene | SCNN1A |
| Shanzhuyu | Alpha-Corocalene | KCNJ8 |
| Shanzhuyu | Alpha-Corocalene | ADRA1A |
| Shanzhuyu | Alpha-Corocalene | SCN1A |
| Shanzhuyu | Alpha-Corocalene | SCNN1G |
| Shanzhuyu | Alpha-Corocalene | HTR2A |
| Shanzhuyu | Alpha-Corocalene | ADRB2 |
| Shanzhuyu | Alpha-Corocalene | ACACB |
| Shanzhuyu | Alpha-Corocalene | MTAP |
| Shanzhuyu | Alpha-Corocalene | ADRA2A |
| Shanzhuyu | Alpha-Corocalene | TLR8 |
| Shanzhuyu | Alpha-Corocalene | ADRB1 |
| Shanzhuyu | Alpha-Corocalene | APRT |
| Shanzhuyu | Alpha-Corocalene | ADRA1D |
| Shanzhuyu | Alpha-Corocalene | PECR |
| Shanzhuyu | Alpha-Corocalene | HRH1 |
| Shanzhuyu | Alpha-Corocalene | PRKAA1 |
| Shanzhuyu | Alpha-Corocalene | HTR7 |
| Shanzhuyu | Alpha-Corocalene | HRH2 |
| Shanzhuyu | Alpha-Corocalene | ADRA1B |
| Shanzhuyu | Alpha-Corocalene | SCNN1D |
| Shanzhuyu | Alpha-Corocalene | ADRA2C |
| Shanzhuyu | Alpha-Corocalene | AOC3 |
| Shanzhuyu | Alpha-Corocalene | P4HA1 |
| Shanzhuyu | Alpha-Corocalene | SRPK2 |
| Shanzhuyu | Alpha-Corocalene | TLR7 |
| Shanzhuyu | Alpha-Corocalene | KCNJ1 |
| Shanzhuyu | Alpha-Corocalene | AGTR1 |
| Shanzhuyu | Alpha-Corocalene | ADRB3 |
| Shanzhuyu | Alpha-Corocalene | ACP1 |
| Shanzhuyu | Alpha-Corocalene | SCNN1B |
| Shanzhuyu | Alpha-Corocalene | CYP2B6 |
| Shanzhuyu | Alpha-Corocalene | ADRA2B |
| Shanzhuyu | Alpha-Corocalene | SLC6A2 |
| Shanzhuyu | Alpha-Corocalene | SAXO1 |
| Shanzhuyu | Alpha-Corocalene | SCT |
| Shanzhuyu | Alpha-Corocalene | UGCG |
| Shanzhuyu | Alpha-Corocalene | PHKG2 |
| Shanzhuyu | Alpha-Corocalene | APLP1 |
| Shanzhuyu | Alpha-Corocalene | ADK |
| Shanzhuyu | Alpha-Corocalene | ACACA |
| Shanzhuyu | Alpha-Corocalene | CHRNA7 |
| Shanzhuyu | Alpha-Corocalene | PNP |
| Shanzhuyu | Alpha-Corocalene | ARRB2 |
| Shanzhuyu | Alpha-Corocalene | RAB7A |
| Shanzhuyu | Alpha-Corocalene | PRKAA2 |
| Shanzhuyu | Alpha-Corocalene | HTR2C |
| Shanzhuyu | Alpha-Corocalene | HSP90AB1 |
| Shanzhuyu | Alpha-Corocalene | ALDH3A2 |
| Shanzhuyu | Alpha-Corocalene | INS |
| Shanzhuyu | Alpha-Corocalene | CALCOCO2 |
| Shanzhuyu | Alpha-Corocalene | SLC22A1 |
| Shanzhuyu | Alpha-Corocalene | AOC2 |
| Shanzhuyu | Alpha-Corocalene | ARRDC3 |
| Shanzhuyu | Alpha-Corocalene | CALCA |
| Shanzhuyu | Alpha-Corocalene | HPRT1 |
| Shanzhuyu | Alpha-Corocalene | ACTN2 |
| Shanzhuyu | Aristolone | CYP17A1 |
| Shanzhuyu | Aristolone | ESR1 |
| Shanzhuyu | Aristolone | PGR |
| Shanzhuyu | Aristolone | OPRK1 |
| Shanzhuyu | Aristolone | NR3C2 |
| Shanzhuyu | Aristolone | PDE7B |
| Shanzhuyu | Aristolone | PDE5A |
| Shanzhuyu | Aristolone | PDE9A |
| Shanzhuyu | Aristolone | PDE4A |
| Shanzhuyu | Aristolone | RYR1 |
| Shanzhuyu | Aristolone | PDE3A |
| Shanzhuyu | Aristolone | ADORA2A |
| Shanzhuyu | Aristolone | ITPR1 |
| Shanzhuyu | Aristolone | PDE6A |
| Shanzhuyu | Aristolone | PRKDC |
| Shanzhuyu | Aristolone | PIK3CD |
| Shanzhuyu | Aristolone | PDE3B |
| Shanzhuyu | Aristolone | PGD |
| Shanzhuyu | Aristolone | PIK3CA |
| Shanzhuyu | Aristolone | ADORA2B |
| Shanzhuyu | Aristolone | PDE1A |
| Shanzhuyu | Aristolone | PDE4D |
| Shanzhuyu | Aristolone | PIK3CB |
| Shanzhuyu | Aristolone | ITPR2 |
| Shanzhuyu | Aristolone | PDE1B |
| Shanzhuyu | Aristolone | PDE7A |
| Shanzhuyu | Aristolone | ITPR3 |
| Shanzhuyu | Aristolone | POLA2 |
| Shanzhuyu | Aristolone | PDE4C |
| Shanzhuyu | Aristolone | PDE10A |
| Shanzhuyu | Aristolone | PDE1C |
| Shanzhuyu | Aristolone | ATM |
| Shanzhuyu | Aristolone | PDE6B |
| Shanzhuyu | Aristolone | PDE4B |
| Shanzhuyu | Aristolone | PDE2A |
| Shanzhuyu | Aristolone | ADORA1 |
| Shanzhuyu | Aristolone | CYP19A1 |
| Shanzhuyu | Aristolone | PDE8B |
| Shanzhuyu | Aristolone | NT5E |
| Shanzhuyu | Aristolone | HDAC2 |
| Shanzhuyu | Aristolone | PDE6C |
| Shanzhuyu | Aristolone | PDE11A |
| Shanzhuyu | Aristolone | PDE8A |
| Shanzhuyu | Aristolone | RINT1 |
| Shanzhuyu | Aristolone | RIPK1 |
| Shanzhuyu | Aristolone | PIK3R1 |
| Shanzhuyu | Aristolone | WNT4 |
| Shanzhuyu | Aristolone | TACR2 |
| Shanzhuyu | Aristolone | AMPD3 |
| Shanzhuyu | Aristolone | CX3CR1 |
| Shanzhuyu | Aristolone | ADA |
| Shanzhuyu | Aristolone | HAP1 |
| Shanzhuyu | Aristolone | IDNK |
| Shanzhuyu | Verbenol | VDR |
| Shanzhuyu | Verbenol | CYP27B1 |
| Shanzhuyu | Verbenol | GC |
| Shanzhuyu | Verbenol | SNW1 |
| Shanzhuyu | Gallicacid | SEC14L3 |
| Shanzhuyu | Gallicacid | PPP2CA |
| Shanzhuyu | Gallicacid | PRKCA |
| Shanzhuyu | Gallicacid | NR1I2 |
| Shanzhuyu | Gallicacid | ALOX5 |
| Shanzhuyu | Gallicacid | PPP2CB |
| Shanzhuyu | Gallicacid | SEC14L2 |
| Shanzhuyu | Gallicacid | DGKA |
| Shanzhuyu | Gallicacid | PRKCB |
| Shanzhuyu | Gallicacid | SEC14L4 |
| Shanzhuyu | Eugenone | GGCX |
| Shanzhuyu | Eugenone | PTGS1 |
| Shanzhuyu | Eugenone | PTGS2 |
| Shanzhuyu | Eugenone | SERPINB7 |
| Shanzhuyu | 1-Allyl-2,4,5-Trimethoxy-Benzene | PDE4A |
| Shanzhuyu | 1-Allyl-2,4,5-Trimethoxy-Benzene | PDE3A |
| Shanzhuyu | 1-Allyl-2,4,5-Trimethoxy-Benzene | CACNA1C |
| Shanzhuyu | 1-Allyl-2,4,5-Trimethoxy-Benzene | TRDMT1 |
| Shanzhuyu | 1-Allyl-2,4,5-Trimethoxy-Benzene | DHFRL1 |
| Shanzhuyu | 1-Allyl-2,4,5-Trimethoxy-Benzene | DHFR |
| Shanzhuyu | 1-Allyl-2,4,5-Trimethoxy-Benzene | PDE10A |
| Shanzhuyu | 1-Allyl-2,4,5-Trimethoxy-Benzene | PDE2A |
| Shanzhuyu | 1-Allyl-2,4,5-Trimethoxy-Benzene | TYMS |
| Shanzhuyu | 1-Allyl-2,4,5-Trimethoxy-Benzene | SHMT1 |
| Shanzhuyu | 1-Allyl-2,4,5-Trimethoxy-Benzene | PDE3B |
| Shanzhuyu | 1-Allyl-2,4,5-Trimethoxy-Benzene | DMTN |
| Shanzhuyu | 1-Allyl-2,4,5-Trimethoxy-Benzene | KCNMA1 |
| Shanzhuyu | Isoamyl Alcohol | REN |
| Shanzhuyu | Isoamyl Alcohol | CACNB1 |
| Shanzhuyu | Isoamyl Alcohol | GRIA4 |
| Shanzhuyu | Isoamyl Alcohol | GABRA2 |
| Shanzhuyu | Isoamyl Alcohol | CACNG2 |
| Shanzhuyu | Isoamyl Alcohol | GABRD |
| Shanzhuyu | Isoamyl Alcohol | CHRNA4 |
| Shanzhuyu | Isoamyl Alcohol | CHRNB3 |
| Shanzhuyu | Isoamyl Alcohol | GABRB1 |
| Shanzhuyu | Isoamyl Alcohol | GRIA1 |
| Shanzhuyu | Isoamyl Alcohol | GLRA2 |
| Shanzhuyu | Isoamyl Alcohol | KCNJ3 |
| Shanzhuyu | Isoamyl Alcohol | GABRG3 |
| Shanzhuyu | Isoamyl Alcohol | VCAM1 |
| Shanzhuyu | Isoamyl Alcohol | CHRNA7 |
| Shanzhuyu | Isoamyl Alcohol | KCNJ6 |
| Shanzhuyu | Isoamyl Alcohol | GABRE |
| Shanzhuyu | Isoamyl Alcohol | GABRA3 |
| Shanzhuyu | Isoamyl Alcohol | SLC29A1 |
| Shanzhuyu | Isoamyl Alcohol | CACNA1S |
| Shanzhuyu | Isoamyl Alcohol | GABRG1 |
| Shanzhuyu | Isoamyl Alcohol | GABRP |
| Shanzhuyu | Isoamyl Alcohol | CHRNA10 |
| Shanzhuyu | Isoamyl Alcohol | GRIA2 |
| Shanzhuyu | Isoamyl Alcohol | GABRA4 |
| Shanzhuyu | Isoamyl Alcohol | CHRNA3 |
| Shanzhuyu | Isoamyl Alcohol | GABRB2 |
| Shanzhuyu | Isoamyl Alcohol | CACNA1C |
| Shanzhuyu | Isoamyl Alcohol | CHRNB2 |
| Shanzhuyu | Isoamyl Alcohol | CACNA1D |
| Shanzhuyu | Isoamyl Alcohol | HTR3D |
| Shanzhuyu | Isoamyl Alcohol | GABRQ |
| Shanzhuyu | Isoamyl Alcohol | CHRNA2 |
| Shanzhuyu | Isoamyl Alcohol | CHRNA6 |
| Shanzhuyu | Isoamyl Alcohol | GABRB3 |
| Shanzhuyu | Isoamyl Alcohol | KCNJ5 |
| Shanzhuyu | Isoamyl Alcohol | GRIN3A |
| Shanzhuyu | Isoamyl Alcohol | HTR3B |
| Shanzhuyu | Isoamyl Alcohol | GRIA3 |
| Shanzhuyu | Isoamyl Alcohol | GABRA5 |
| Shanzhuyu | Isoamyl Alcohol | SLC29A2 |
| Shanzhuyu | Isoamyl Alcohol | HTR3E |
| Shanzhuyu | Isoamyl Alcohol | GABRA6 |
| Shanzhuyu | Isoamyl Alcohol | HTR3A |
| Shanzhuyu | Isoamyl Alcohol | GABRA1 |
| Shanzhuyu | Isoamyl Alcohol | CHRNA9 |
| Shanzhuyu | Isoamyl Alcohol | CHRFAM7A |
| Shanzhuyu | Isoamyl Alcohol | CHRNA5 |
| Shanzhuyu | Isoamyl Alcohol | CHRNB4 |
| Shanzhuyu | Isoamyl Alcohol | KCNJ9 |
| Shanzhuyu | Isoamyl Alcohol | HTR3C |
| Shanzhuyu | Isoamyl Alcohol | GLRA1 |
| Shanzhuyu | Isoamyl Alcohol | CACNG1 |
| Shanzhuyu | Isoamyl Alcohol | GLRA3 |
| Shanzhuyu | Isoamyl Alcohol | ADAM8 |
| Shanzhuyu | Isoamyl Alcohol | SLC28A3 |
| Shanzhuyu | Isoamyl Alcohol | ADRA1B |
| Shanzhuyu | Isoamyl Alcohol | FOXL2 |
| Shanzhuyu | Isoamyl Alcohol | DMTN |
| Shanzhuyu | Isoamyl Alcohol | KCNMA1 |
| Shanzhuyu | Isobutyl Alcohol | TRPV3 |
| Shanzhuyu | Isobutyl Alcohol | OPRK1 |
| Shanzhuyu | Isobutyl Alcohol | TRPM8 |
| Shanzhuyu | Isobutyl Alcohol | TRPA1 |
| Shanzhuyu | Isobutyl Alcohol | KCNK4 |
| Shanzhuyu | Isotetrandrine | ADRA1A |
| Shanzhuyu | Isotetrandrine | HTR2A |
| Shanzhuyu | Isotetrandrine | CHRM3 |
| Shanzhuyu | Isotetrandrine | CHRM1 |
| Shanzhuyu | Isotetrandrine | ADRA2A |
| Shanzhuyu | Isotetrandrine | HTR2C |
| Shanzhuyu | Isotetrandrine | ADRA1D |
| Shanzhuyu | Isotetrandrine | DRD2 |
| Shanzhuyu | Isotetrandrine | CHRM5 |
| Shanzhuyu | Isotetrandrine | HRH1 |
| Shanzhuyu | Isotetrandrine | ACE |
| Shanzhuyu | Isotetrandrine | HTR7 |
| Shanzhuyu | Isotetrandrine | ADRA1B |
| Shanzhuyu | Isotetrandrine | ADRA2C |
| Shanzhuyu | Isotetrandrine | CHRM2 |
| Shanzhuyu | Isotetrandrine | CHRM4 |
| Shanzhuyu | Isotetrandrine | ADRA2B |
| Shanzhuyu | Isotetrandrine | SLC6A2 |
| Shanzhuyu | Isotetrandrine | ACHE |
| Shanzhuyu | Isotetrandrine | CHRNA2 |
| Shanzhuyu | Isotetrandrine | HTR3A |
| Shanzhuyu | 2-Furancarboxylic Acid | PTGS1 |
| Shanzhuyu | 2-Furancarboxylic Acid | PTGS2 |
| Shanzhuyu | 2-Furancarboxylic Acid | SERPINB7 |
| Shanzhuyu | Retinol | RDH11 |
| Shanzhuyu | Retinol | RBP3 |
| Shanzhuyu | Retinol | RETSAT |
| Shanzhuyu | Retinol | RDH13 |
| Shanzhuyu | Retinol | DHRS3 |
| Shanzhuyu | Retinol | RDH5 |
| Shanzhuyu | Retinol | ALDH1A3 |
| Shanzhuyu | Retinol | RDH12 |
| Shanzhuyu | Retinol | DHRS4 |
| Shanzhuyu | Retinol | RBP1 |
| Shanzhuyu | Retinol | ALDH1A1 |
| Shanzhuyu | Retinol | RLBP1 |
| Shanzhuyu | Retinol | RDH14 |
| Shanzhuyu | Retinol | RDH8 |
| Shanzhuyu | Retinol | LRAT |
| Shanzhuyu | Retinol | ALDH1A2 |
| Shanzhuyu | Retinol | ALDH3A1 |
| Shanzhuyu | Retinol | RHO |
| Shanzhuyu | Retinol | RS1 |
| Shanzhuyu | Isoamylamine | GRIN2A |
| Shanzhuyu | Isoamylamine | DRD2 |
| Shanzhuyu | Isoamylamine | GRIN2B |
| Shanzhuyu | Isoamylamine | GRIN3A |
| Shanzhuyu | Isoamylamine | HTR3A |
| Shanzhuyu | Isoamylamine | GRIN1 |
| Shanzhuyu | Isoamylamine | CNR1 |
| Shanzhuyu | Isoamylamine | EPHX2 |
| Shanzhuyu | Isoamylamine | CD34 |
| Tusizi | sesamin | DECR1 |
| Tusizi | kaempferol | PSMD3 |
| Tusizi | quercetin | PSMD3 |
| Tusizi | sesamin | ECI1 |
| Tusizi | Isofucosterol | ABAT |
| Tusizi | beta-sitosterol | HTR2A |
| Tusizi | matrine | MMP2 |
| Tusizi | quercetin | MMP2 |
| Tusizi | quercetin | HSPA5 |
| Tusizi | isorhamnetin | ACHE |
| Tusizi | kaempferol | ACHE |
| Tusizi | quercetin | ACHE |
| Tusizi | sesamin | ACACA |
| Tusizi | quercetin | ACACA |
| Tusizi | kaempferol | AHSA1 |
| Tusizi | quercetin | AHSA1 |
| Tusizi | Isofucosterol | ADH1A |
| Tusizi | Isofucosterol | ADH1B |
| Tusizi | Isofucosterol | ADH1C |
| Tusizi | kaempferol | AKR1C3 |
| Tusizi | isorhamnetin | AKR1B10 |
| Tusizi | quercetin | AKR1B10 |
| Tusizi | beta-sitosterol | ADRA1A |
| Tusizi | beta-sitosterol | ADRA1B |
| Tusizi | kaempferol | ADRA1B |
| Tusizi | isorhamnetin | MAOB |
| Tusizi | quercetin | MAOB |
| Tusizi | isorhamnetin | AR |
| Tusizi | kaempferol | AR |
| Tusizi | quercetin | AR |
| Tusizi | kaempferol | SLPI |
| Tusizi | beta-sitosterol | BAX |
| Tusizi | kaempferol | BAX |
| Tusizi | quercetin | BAX |
| Tusizi | beta-sitosterol | BCL2 |
| Tusizi | kaempferol | BCL2 |
| Tusizi | quercetin | BCL2 |
| Tusizi | kaempferol | ALOX5 |
| Tusizi | quercetin | ALOX5 |
| Tusizi | kaempferol | AHR |
| Tusizi | quercetin | AHR |
| Tusizi | quercetin | ABCG1 |
| Tusizi | sesamin | ACLY |
| Tusizi | quercetin | BIRC5 |
| Tusizi | quercetin | BCL2L1 |
| Tusizi | beta-sitosterol | ADRB2 |
| Tusizi | quercetin | ADRB2 |
| Tusizi | isorhamnetin | CALM1 |
| Tusizi | kaempferol | CALM1 |
| Tusizi | beta-sitosterol | CASP3 |
| Tusizi | kaempferol | CASP3 |
| Tusizi | matrine | CASP3 |
| Tusizi | quercetin | CASP3 |
| Tusizi | beta-sitosterol | CASP8 |
| Tusizi | quercetin | CASP8 |
| Tusizi | beta-sitosterol | CASP9 |
| Tusizi | quercetin | CASP9 |
| Tusizi | quercetin | CTSD |
| Tusizi | quercetin | CAV1 |
| Tusizi | quercetin | CCL2 |
| Tusizi | quercetin | CD40LG |
| Tusizi | matrine | CD44 |
| Tusizi | kaempferol | CDK1 |
| Tusizi | quercetin | CDK1 |
| Tusizi | isorhamnetin | CDK2 |
| Tusizi | quercetin | TP53 |
| Tusizi | beta-sitosterol | PDE3A |
| Tusizi | quercetin | CLDN4 |
| Tusizi | isorhamnetin | F7 |
| Tusizi | kaempferol | F7 |
| Tusizi | quercetin | F7 |
| Tusizi | sesamin | F10 |
| Tusizi | quercetin | F10 |
| Tusizi | quercetin | COL19A1 |
| Tusizi | quercetin | COL3A1 |
| Tusizi | quercetin | CRP |
| Tusizi | quercetin | CXCL10 |
| Tusizi | quercetin | CXCL11 |
| Tusizi | quercetin | CXCL2 |
| Tusizi | isorhamnetin | CCNA2 |
| Tusizi | quercetin | CDKN1A |
| Tusizi | kaempferol | CYP1A1 |
| Tusizi | quercetin | CYP1A1 |
| Tusizi | kaempferol | CYP1A2 |
| Tusizi | quercetin | CYP1A2 |
| Tusizi | kaempferol | CYP1B1 |
| Tusizi | quercetin | CYP1B1 |
| Tusizi | sesamin | CYP2B6 |
| Tusizi | kaempferol | CYP3A4 |
| Tusizi | quercetin | CYP3A4 |
| Tusizi | quercetin | DCAF5 |
| Tusizi | isorhamnetin | DPP4 |
| Tusizi | kaempferol | DPP4 |
| Tusizi | quercetin | DPP4 |
| Tusizi | quercetin | TOP2A |
| Tusizi | quercetin | TOP1 |
| Tusizi | kaempferol | TOP2A |
| Tusizi | beta-sitosterol | DRD5 |
| Tusizi | quercetin | DUOX2 |
| Tusizi | sesamin | ECE1 |
| Tusizi | quercetin | EGFR |
| Tusizi | kaempferol | SELE |
| Tusizi | quercetin | SELE |
| Tusizi | isorhamnetin | ESR1 |
| Tusizi | isorhamnetin | ESR2 |
| Tusizi | quercetin | SULT1E1 |
| Tusizi | quercetin | ELK1 |
| Tusizi | quercetin | EIF6 |
| Tusizi | sesamin | FASN |
| Tusizi | sesamin | CCND1 |
| Tusizi | quercetin | CCND1 |
| Tusizi | quercetin | CCNB1 |
| Tusizi | isorhamnetin | GABRA1 |
| Tusizi | beta-sitosterol | GABRA1 |
| Tusizi | kaempferol | GABRA1 |
| Tusizi | Isofucosterol | GABRA1 |
| Tusizi | quercetin | GABRA1 |
| Tusizi | beta-sitosterol | GABRA2 |
| Tusizi | kaempferol | GABRA2 |
| Tusizi | beta-sitosterol | GABRA3 |
| Tusizi | quercetin | GJA1 |
| Tusizi | sesamin | G6PD |
| Tusizi | isorhamnetin | GRIA2 |
| Tusizi | kaempferol | GSTM1 |
| Tusizi | quercetin | GSTM1 |
| Tusizi | kaempferol | GSTM2 |
| Tusizi | quercetin | GSTM2 |
| Tusizi | kaempferol | GSTP1 |
| Tusizi | quercetin | GSTP1 |
| Tusizi | isorhamnetin | PYGM |
| Tusizi | isorhamnetin | GSK3B |
| Tusizi | quercetin | HSF1 |
| Tusizi | quercetin | HSPB1 |
| Tusizi | isorhamnetin | HSP90AB1 |
| Tusizi | beta-sitosterol | HSP90AB1 |
| Tusizi | kaempferol | HSP90AB1 |
| Tusizi | quercetin | HSP90AB1 |
| Tusizi | kaempferol | HMOX1 |
| Tusizi | quercetin | HMOX1 |
| Tusizi | matrine | HPSE |
| Tusizi | quercetin | HK2 |
| Tusizi | quercetin | NKX3-1 |
| Tusizi | kaempferol | HAS2 |
| Tusizi | quercetin | HAS2 |
| Tusizi | quercetin | HIF1A |
| Tusizi | matrine | IER3IP1 |
| Tusizi | quercetin | CHUK |
| Tusizi | kaempferol | IKBKB |
| Tusizi | kaempferol | INSR |
| Tusizi | quercetin | INSR |
| Tusizi | quercetin | IGF2 |
| Tusizi | quercetin | IGFBP3 |
| Tusizi | kaempferol | ICAM1 |
| Tusizi | matrine | ICAM1 |
| Tusizi | quercetin | ICAM1 |
| Tusizi | quercetin | IFNG |
| Tusizi | quercetin | IRF1 |
| Tusizi | quercetin | IL1A |
| Tusizi | quercetin | IL1B |
| Tusizi | sesamin | IL10 |
| Tusizi | quercetin | IL10 |
| Tusizi | quercetin | IL2 |
| Tusizi | matrine | IL6 |
| Tusizi | quercetin | IL6 |
| Tusizi | quercetin | CXCL8 |
| Tusizi | kaempferol | MMP1 |
| Tusizi | quercetin | MMP1 |
| Tusizi | quercetin | MGAM |
| Tusizi | quercetin | MMP9 |
| Tusizi | sesamin | ACADM |
| Tusizi | sesamin | AUH |
| Tusizi | beta-sitosterol | MAP2 |
| Tusizi | Isofucosterol | NR3C2 |
| Tusizi | CLR | NR3C2 |
| Tusizi | quercetin | MAPK1 |
| Tusizi | isorhamnetin | MAPK14 |
| Tusizi | kaempferol | MAPK8 |
| Tusizi | isorhamnetin | PRKACA |
| Tusizi | beta-sitosterol | PRKACA |
| Tusizi | kaempferol | PRKACA |
| Tusizi | quercetin | PRKACA |
| Tusizi | isorhamnetin | PTPN1 |
| Tusizi | beta-sitosterol | CHRM1 |
| Tusizi | kaempferol | CHRM1 |
| Tusizi | beta-sitosterol | CHRM2 |
| Tusizi | kaempferol | CHRM2 |
| Tusizi | beta-sitosterol | CHRM3 |
| Tusizi | beta-sitosterol | CHRM4 |
| Tusizi | beta-sitosterol | OPRM1 |
| Tusizi | matrine | MYC |
| Tusizi | quercetin | MYC |
| Tusizi | quercetin | MPO |
| Tusizi | quercetin | NQO1 |
| Tusizi | sesamin | NOX1 |
| Tusizi | sesamin | NOX3 |
| Tusizi | quercetin | POR |
| Tusizi | beta-sitosterol | CHRNA7 |
| Tusizi | beta-sitosterol | CHRNA2 |
| Tusizi | isorhamnetin | NCF1 |
| Tusizi | quercetin | NCF1 |
| Tusizi | quercetin | NFKBIA |
| Tusizi | sesamin | NOS3 |
| Tusizi | quercetin | NOS3 |
| Tusizi | isorhamnetin | NOS2 |
| Tusizi | kaempferol | NOS2 |
| Tusizi | isorhamnetin | NOS3 |
| Tusizi | kaempferol | NOS3 |
| Tusizi | quercetin | NFE2L2 |
| Tusizi | isorhamnetin | NCOA1 |
| Tusizi | isorhamnetin | NCOA2 |
| Tusizi | beta-sitosterol | NCOA2 |
| Tusizi | kaempferol | NCOA2 |
| Tusizi | Isofucosterol | NCOA2 |
| Tusizi | CLR | NCOA2 |
| Tusizi | quercetin | NCOA2 |
| Tusizi | kaempferol | NR1I2 |
| Tusizi | quercetin | NR1I2 |
| Tusizi | kaempferol | NR1I3 |
| Tusizi | quercetin | NR1I3 |
| Tusizi | quercetin | ODC1 |
| Tusizi | quercetin | SPP1 |
| Tusizi | isorhamnetin | OLR1 |
| Tusizi | sesamin | ACOX1 |
| Tusizi | sesamin | EHHADH |
| Tusizi | isorhamnetin | PPARD |
| Tusizi | isorhamnetin | PPARG |
| Tusizi | kaempferol | PPARG |
| Tusizi | quercetin | PPARG |
| Tusizi | quercetin | PPARA |
| Tusizi | quercetin | PPARD |
| Tusizi | quercetin | PTEN |
| Tusizi | isorhamnetin | PIK3CG |
| Tusizi | beta-sitosterol | PIK3CG |
| Tusizi | kaempferol | PIK3CG |
| Tusizi | quercetin | PIK3CG |
| Tusizi | quercetin | SERPINE1 |
| Tusizi | quercetin | TNKS |
| Tusizi | beta-sitosterol | KCNH2 |
| Tusizi | quercetin | KCNH2 |
| Tusizi | quercetin | PCOLCE |
| Tusizi | quercetin | EGF |
| Tusizi | NSC63551 | PGR |
| Tusizi | beta-sitosterol | PGR |
| Tusizi | kaempferol | PGR |
| Tusizi | campest-5-en-3beta-ol | PGR |
| Tusizi | Isofucosterol | PGR |
| Tusizi | CLR | PGR |
| Tusizi | quercetin | PTGER3 |
| Tusizi | isorhamnetin | PTGS1 |
| Tusizi | beta-sitosterol | PTGS1 |
| Tusizi | kaempferol | PTGS1 |
| Tusizi | quercetin | PTGS1 |
| Tusizi | sesamin | PTGS2 |
| Tusizi | isorhamnetin | PTGS2 |
| Tusizi | beta-sitosterol | PTGS2 |
| Tusizi | kaempferol | PTGS2 |
| Tusizi | quercetin | PTGS2 |
| Tusizi | quercetin | ACP3 |
| Tusizi | quercetin | RUNX1T1 |
| Tusizi | beta-sitosterol | PRKCA |
| Tusizi | quercetin | PRKCA |
| Tusizi | quercetin | PRKCB |
| Tusizi | quercetin | FOS |
| Tusizi | isorhamnetin | PIM1 |
| Tusizi | quercetin | NPEPPS |
| Tusizi | kaempferol | AKT1 |
| Tusizi | quercetin | AKT1 |
| Tusizi | quercetin | RAF1 |
| Tusizi | quercetin | RASSF1 |
| Tusizi | quercetin | RASA1 |
| Tusizi | quercetin | ERBB2 |
| Tusizi | quercetin | ERBB3 |
| Tusizi | quercetin | RB1 |
| Tusizi | quercetin | RXRA |
| Tusizi | quercetin | RUNX2 |
| Tusizi | isorhamnetin | CHEK1 |
| Tusizi | quercetin | CHEK2 |
| Tusizi | kaempferol | PPP3CA |
| Tusizi | beta-sitosterol | PON1 |
| Tusizi | quercetin | PON1 |
| Tusizi | kaempferol | STAT1 |
| Tusizi | quercetin | STAT1 |
| Tusizi | sesamin | SCN5A |
| Tusizi | beta-sitosterol | SCN5A |
| Tusizi | quercetin | SCN5A |
| Tusizi | kaempferol | SLC6A2 |
| Tusizi | beta-sitosterol | SLC6A4 |
| Tusizi | kaempferol | SLC2A4 |
| Tusizi | quercetin | SLC2A4 |
| Tusizi | sesamin | SREBF1 |
| Tusizi | quercetin | MMP3 |
| Tusizi | quercetin | SOD1 |
| Tusizi | isorhamnetin | F2 |
| Tusizi | kaempferol | F2 |
| Tusizi | quercetin | F2 |
| Tusizi | quercetin | THBD |
| Tusizi | quercetin | F3 |
| Tusizi | quercetin | PLAT |
| Tusizi | beta-sitosterol | JUN |
| Tusizi | kaempferol | JUN |
| Tusizi | quercetin | JUN |
| Tusizi | quercetin | E2F1 |
| Tusizi | quercetin | E2F2 |
| Tusizi | isorhamnetin | RELA |
| Tusizi | kaempferol | RELA |
| Tusizi | matrine | RELA |
| Tusizi | quercetin | RELA |
| Tusizi | beta-sitosterol | TGFB1 |
| Tusizi | quercetin | TGFB1 |
| Tusizi | sesamin | HADHB |
| Tusizi | isorhamnetin | PRSS1 |
| Tusizi | kaempferol | PRSS1 |
| Tusizi | quercetin | PRSS1 |
| Tusizi | kaempferol | TNF |
| Tusizi | matrine | TNF |
| Tusizi | quercetin | TNF |
| Tusizi | kaempferol | DIO1 |
| Tusizi | quercetin | DIO1 |
| Tusizi | sesamin | UGT1A1 |
| Tusizi | quercetin | PLAU |
| Tusizi | kaempferol | VCAM1 |
| Tusizi | quercetin | VCAM1 |
| Tusizi | quercetin | VEGFA |
| Tusizi | isorhamnetin | XDH |
| Tusizi | kaempferol | XDH |
| Tusizi | quercetin | XDH |
| Tusizi | Campesterol | ESR1 |
| Tusizi | Campesterol | PGR |
