# Supplementary material for: Zuogui Pill Ameliorates Glucocorticoid-Induced Osteoporosis through ZNF702P-Based ceRNA Network: Bioinformatics Analysis and Experimental Validation
Source: Evid Based Complement Alternat Med. 2022 Aug 29;2022:8020182. doi: 10.1155/2022/8020182 (PMC11401717; doi:10.1155/2022/8020182)
Supplement: Supplementary Materials — Supplementary Table 1. LncRNA-miRNA-mRNA interactions. Supplementary Table 2. The drugs, active compounds, and targets of ZGP. Supplementary Table 3. Detailed network topology information of intersection targets. [file 8020182.f1.zip › 8020182.f1/Supplementary Table 3 Detailed network topology information of intersection targets.doc]

Supplementary Table 3. Detailed network topology information of intersection targets

| Targets | Degree | BetweennessCentrality |
| --- | --- | --- |
| JUN | 7 | 0.190476 |
| CCND1 | 6 | 0.095238 |
| MAPK1 | 6 | 0.047619 |
| MAPK14 | 6 | 0.047619 |
| RB1 | 5 | 0 |
| CDKN1A | 5 | 0 |
| IFNG | 3 | 0 |
| CHUK | 2 | 0 |
| Average | 5 | 0.047619 |
